# Chemical Recycling of Polyesters and Polycarbonates: Why Is Zinc(II) Such an Effective Depolymerization Catalyst?

**DOI:** 10.1021/jacs.5c16346

**Published:** 2025-11-10

**Authors:** Thomas M. McGuire, Antoine Buchard, Charlotte K. Williams

**Affiliations:** † Department of Chemistry, Chemistry Research Laboratory, 12 Mansfield Road, 150602 University of Oxford, Oxford OX1 3TA, United Kingdom; ‡ Department of Chemistry, Green Chemistry Centre of Excellence, 8748 University of York, York YO10 5DD, United Kingdom

## Abstract

Effective polymer recycling is essential to reduce plastic pollution; catalytic polymer recycling to monomer is particularly attractive, as it could operate over multiple closed-loop cycles. Aliphatic polyesters and carbonates show properties that compete with current plastics and can be depolymerized to 6- and 7-membered cyclic ester or carbonate monomers. Nonetheless, the rules governing recycling catalyst selection are unclear. Here, Zn­(II), Co­(II), Mg­(II), Sn­(II), Ca­(II), Ba­(II), Y­(III) and Bi­(III) 2-ethyl hexanoate catalysts are compared for the chemical recycling of 6 different oxygenated polymers, in bulk, at low catalyst loadings (1:100 to 1:1000) and temperatures (90–170 °C). All metals are selective for recycling to monomer but show clear differences in rates; the Zn­(II) catalyst is always the most active. Using linear free energy analysis, the depolymerization rate constant directly correlates with the metal’s Lewis acidity, as assessed by its hydrolysis constant. The best catalysts comprise metals with intermediate acidity, i.e., Zn­(II), Co­(II) and Mg­(II). The structure–activity correlation applies to polymers that have primary or secondary chain-end group alcohols, 6- or 7-atom repeat units, and those featuring ester or carbonate linkages. Eyring analysis using Zn­(II), Co­(II), Mg­(II) and Sn­(II) catalysts shows that the Zn­(II) catalysts balance competing transition-state enthalpy (Δ*H* ^‡^_d_) and entropy (Δ*S* ^‡^_d_) demands. Density functional theory calculations of key transition states suggest that Zn­(II) is particularly effective because it both activates the polymer carbonyl group and labilizes the alkoxide nucleophile. These generally applicable linear free energy relationships are important tools to minimize energy input and maximize performances in future recycling processes.

## Graphical abstract

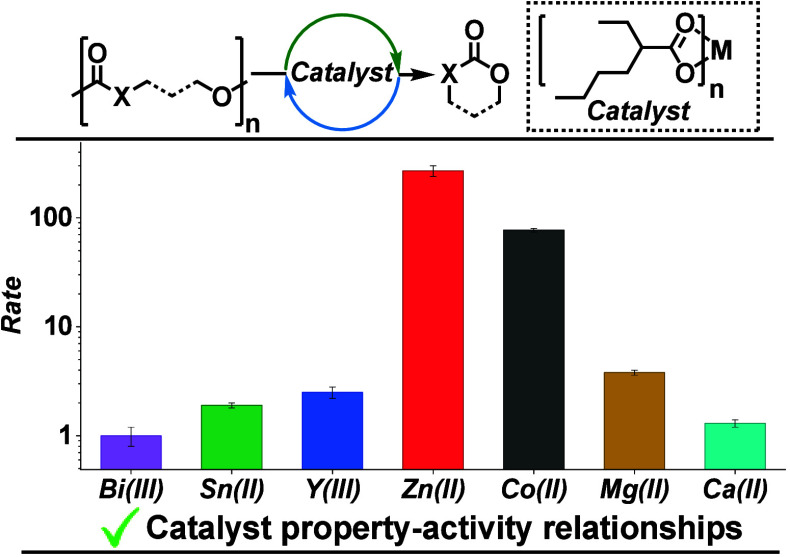

## Introduction

The recovery of energy and materials via polymer recycling is essential to mitigate pollution and deliver a circular economy for polymers.^1,2^ While many different recycling approaches will be needed, chemical recycling to monomer is particularly attractive since it obviates losses in material function hampering mechanical recycling and helps preserve some of the embedded energy and emissions deriving from virgin monomer production.^3^ Such chemical recycling to monomer can become thermodynamically feasible by manipulating polymer-monomer equilibria, i.e. by selecting conditions which favor depolymerization.^4^ In most cases, chain depolymerization is enthalpically disfavored (ΔH_p_ > 0) but entropically favored (ΔS_p_ > 0) (i.e., the reverse constraints to polymerization). It is, therefore, essential to select appropriate conditions for thermodynamically feasible recycling. Applying conditions to ensure the depolymerization kinetics are optimal is equally essential, particularly where high temperatures may result in decomposition side processes. Aliphatic polyesters and carbonates, especially those derived from cyclic ester or carbonate ring-opening polymerization (ROP), have depolymerization thermodynamics which enable selective monomer formation at 100–200 °C.^5^ Many of these materials are already commercialized and widely studied in academic laboratories due to their excellent properties and, in some cases, compatibility with preparations starting from renewable feedstocks.^6^ For example, polyvalerolactone (PVL), which can be accessed from bioderived 5-hydroxypentanoic acid,^7^ shows tensile strengths and ductility similar to polyethylene and with reasonable high temperature stability (*T*_d_ > 300 °C). When PVL is combined with the right catalysts it can be efficiently recycled at just 100 °C to valerolactone (which is the monomer used to make PVL).^8^

The synthesis and properties of these aliphatic polyesters and polycarbonates are now sufficiently advanced that they can compete as plastics,^9,10^ elastomers^11^ and adhesives.^12^ The advances in polymer property and application development motivate research into understanding their recycling catalysis. Recycling processes should be designed to show high monomer conversion, rates and selectivity, with minimal energy inputs, i.e., at low catalyst loadings and temperatures. These catalyzed chain depolymerizations should apply neat polymer melts since these are likely compatible with larger-scale recycling infrastructure and may help to minimize impacts associated with solvents and separations.^13–15^ However, so far most polyester/carbonate recycling studies have been proof-of-concept investigations conducted in organic solvents, often at high polymer dilution, and often applying undesirably high catalyst loadings.^16–20^ Consequently, there is a need for a clear understanding of the factors that affect the kinetics of depolymerization, including direct measurements and comparisons of recycling rate constants for various catalysts and polymers.

Prior research suggests Zn­(II), Mg­(II) or Sn­(II) halide, acetate and 2-ethylhexanoate catalysts all show fast rates in these types of oxygenated polymer depolymerizations,^16,21–23^ although how to match the right metal catalyst to a target polymer remains unclear. For example, depolymerizations of polyvalerolactone (PVL) and polycaprolactone (PCL), conducted under comparable conditions using neat polymer, 100–180 °C, 1–2 mol % catalyst, resulted in conflicting conclusions with some authors reporting Zn­(II) is the best catalyst,^8,22,24^ while others find Mg­(II) to be more efficient.^21,25^ One solution would be to establish catalyst structure-performance correlations.^26–28^

Very recently, we showed a generally applicable linear free energy relationship between polymer-monomer thermodynamic parameters (depolymerization equilibrium constant, K_d_ or free energy change, ΔG_d_) and recycling rates (*k*_*d*_ or ΔG^‡^_d_).^29^ Accordingly, the depolymerization equilibrium constant, K_eq_, correlates exponentially to recycling rate constant, *k*_*d*_, for a series of different polyesters and polycarbonates, all analyzed using Zn­(Oct)_2_ as the catalyst. The investigation revealed that oxygenated polymers that have more favorable depolymerization equilibria also show faster depolymerization rates. For example, under the same recycling conditions, PVL, which has an ∼ 10x fold higher equilibrium conversion to lactone than PCL, was recycled to its monomer around 100x faster than PCL. The study also correlated the polymer structural features, including repeat unit length, chemistry, and end-group, with the depolymerization rate. The prior research was only conducted using a single catalyst, Zn­(II)­(Oct)_2_, and did not address catalyst structure-performance relationships.

Here, we investigate the depolymerization catalyst structure-performance relationships. A series of recycling catalysts are selected, all are metal­(2-ethylhexanoate) complexes, where metal = Zn­(II), Mg­(II), Co­(II), Sn­(II), Ca­(II), Ba­(II), Bi­(III), and Y­(III). The metals are selected so as to cover a systematic range of values for metallic ionic radii, oxophilicity and acidity values – these quantified descriptors will be used in constructing structure–activity plots (Figure 1a and b). As a further benefit, all catalysts are commercial and show good solubility (in both solvents and the polymers/monomers targeted). A systematic series of different polyesters and carbonates are selected for testing using these catalysts, with experiments designed to enable continual measurements of conversion vs time so as to quantify depolymerization rates (Figure 1 c and d). The rate data will be used to identify structure-performance relationships by plotting recycling kinetic parameters against metal catalyst descriptors.

**Figure 1:**
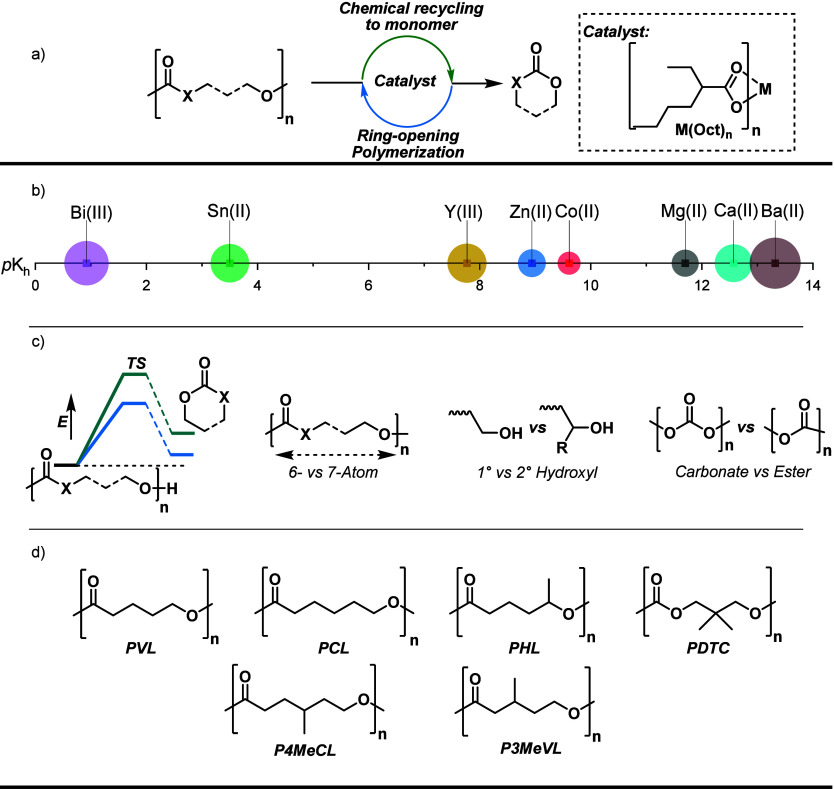
(a) Depolymerization of oxygenated polymers to monomer using metal­(2-ethylhexanoate) catalysts. (b) Systematic variation of metal properties to delineate structure-rate relationships; squares indicate the values for the metal hydrolysis constant, while the circles approximate the metal’s ionic radius (6-coordinate). All sizes are shown to scale. (c) Key polymer properties and structural modifications that influence depolymerization rate: linker length, chain-end group chemistry, and polymer carbonyl group. (d) Illustrates the polymers used in this study. Structures allow for investigation into effects of linker length (6-atom vs 7-atom), end-group (primary hydroxyl vs secondary hydroxyl) and carbonyl electrophile (ester vs carbonate linkage) on depolymerization catalysis. All polymers are dihydroxy telechelic with a degree of polymerization of *ca.* 100.

## Results and Discussion

First, each of the metal catalysts was tested, under comparable conditions, for the depolymerization of polyvalerolactone (PVL) to its monomer valerolactone (VL). PVL was chosen as a leading polymer substrate since it is already a commercial product, can be bioderived, and shows tensile mechanical properties akin to those of polypropylene and polyethylene.^8^ To ensure consistent PVL features in the recycling experiments, a large batch of dihydroxy telechelic PVL was prepared with a degree of polymerization of *ca.* 100 (Table S3, *DP*_NMR_ = 104; *M*_n,SEC_ = 17,500 g mol^–1,^ *Đ*_M_ = 1.09). In the absence of any recycling catalyst, the pure PVL is stable up to a temperature of 324 °C.

To investigate PVL recycling catalysis, films containing catalyst: PVL loadings of 1:1000 (where 1000 is the number of polymer repeat units) were prepared from THF solutions and thoroughly dried to remove the residual solvent (see SI for full details). The PVL recycling rate was determined at 130 °C using thermogravimetric analysis, under N_2_ flows of 25 mL/min. In all cases, the complete depolymerization reactions occurred at temperatures significantly below the degradation onset of the pure polymer, indicative of catalyzed polymer recycling (Figure 2a, Table S4, Figure S2). For all catalysts, the recycling conversion, i.e. (1– fraction mass loss), increased with respect to time. The depolymerization rate constant, *k*_obs_, was determined using a sigmoidal fit to the mass loss vs time data over the full extent of reaction.^29^ All reactions were repeated at least twice to determine the rate error ranges. The *k*_obs_ and turnover frequency (TOF, all reported at 30% conversion) values are both used to evaluate catalyst performances. The two best catalysts are Zn­(Oct)_2_ and Co­(Oct)_2_ which both showed remarkable rates, with TOF values of 6600 and 2300 h^–1^, respectively. These catalysts show *k*_obs_ values of 2.7 s^–1^ for Zn­(II) and 0.77 × 10^–3^ s^–1^ for Co­(II), respectively (Figure 2b). The analogous Mg­(II) catalyst was slower but still showed impressive activity, with a TOF of 100 h^–1^ and *k*_obs_ = 0.038 × 10^–3^ s^–1^. The other catalysts, Ca­(II), Y­(III), Sn­(II), Ba­(II) and Bi­(III), were all considerably slower. The depolymerization products were continually analyzed by FTIR spectroscopy: all the catalysts showed quantitative selectivity for valerolactone formation (Figure S3). Using the lead Zn­(II) catalyst, a larger-scale polymer catalyst film (*ca* 1g, 1:1000) was heated to 130 °C, under dynamic vacuum, and the monomer, VL was isolated in in high yield and purity as determined by ^1^H NMR spectroscopy and GC-MS. The lab-scale recycling experiment confirmed the utility of the smaller-scale TGA-IR experiments for measurement of depolymerization rates (99% yield, > 99% selectivity, Figure S4–5).^29^ Overall, these results reveal that the catalyst metal center has a significant influence over the rate of depolymerization. The order of rates for the catalysts is (from fastest to slowest):

Zn(II)>Co(II)>Mg(II)>Ca(II)>Y(III)>Sn(II)>Ba(II)>Bi(III)

**Figure 2:**
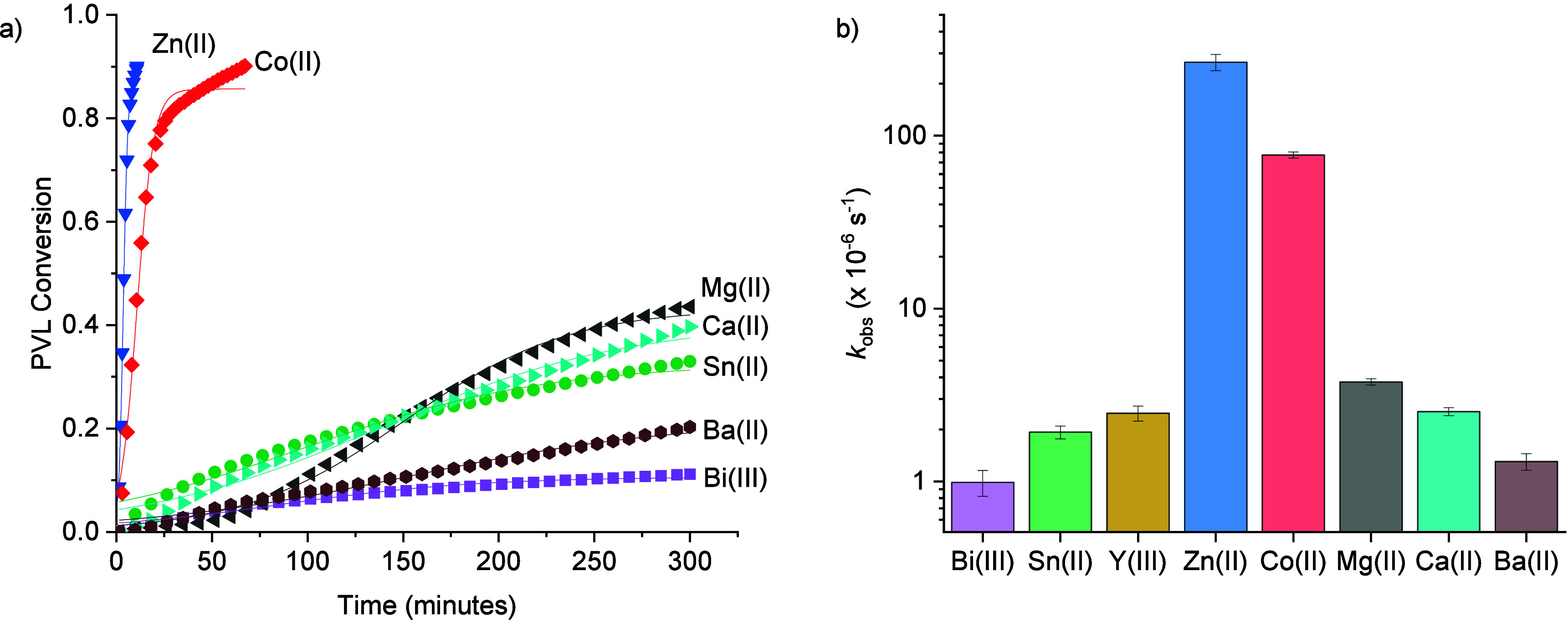
Depolymerization of polyvalerolactone (PVL, structure shown in Figure 1d) using different metal catalysts, where [catalyst]_0_:[PVL]_0_ = 1:1000, 130 °C. (a) Plots of PVL conversion vs time, for the different metal catalysts, with the sigmoidal fits (the data for Y­(III) is shown in Figure S2). (b) Bar chart showing the depolymerization rate constant, *k*_*obs*_, vs metal catalyst (note the logarithmic scale on the *y*-axis).

Next, each of these catalysts was tested in the depolymerization of a series of 6- and 7-atom linker polyesters and polycarbonates (Figure 3). The polymers were selected to systematically investigate and compare against the results obtained for PVL, targeting information on effects of changes to the chain linker length, hydroxyl chain-end group chemistry, and the carbonyl group electrophilicity on the recycling rates (Figure 1 c). These polymers include polycaprolactone PCL, with its increased linker length to 7-atoms (PVL = 6-atom linker), polyhexalactone PHL, with the same 6-atom linker but a secondary alcohol end group (PVL = primary hydroxyl end-group) and poly­(3,3-dimethyltrimethylenecarbonate) PDTC, with the same linker length and end-group but featuring a carbonate electrophile (PVL = ester group). Each of the test polymers was prepared by ring-opening polymerization of the respective lactone or cyclic carbonate (Table S3). To standardize the experiments, all of the polymer samples are dihydroxy-telechelic with degrees of polymerization of *ca.* 100. In the absence of catalyst, all the polymers are stable to temperatures >200 °C. Films of the appropriate polymer and catalyst were prepared, and the recycling rates were evaluated using the same TGA-FTIR spectroscopy methodology (Figure 3). To account for the expected slower rates using PCL and PHL, a higher catalyst:polymer loading was applied (1:100), while the same low catalyst loading (1:1000) used for PVL was also used in PDTC recycling experiments (Figures S6–11, Tables S5–7). The depolymerizations of PHL and PDTC were performed at 150 °C, while those using PCL were performed at 160 °C.

**Figure 3:**
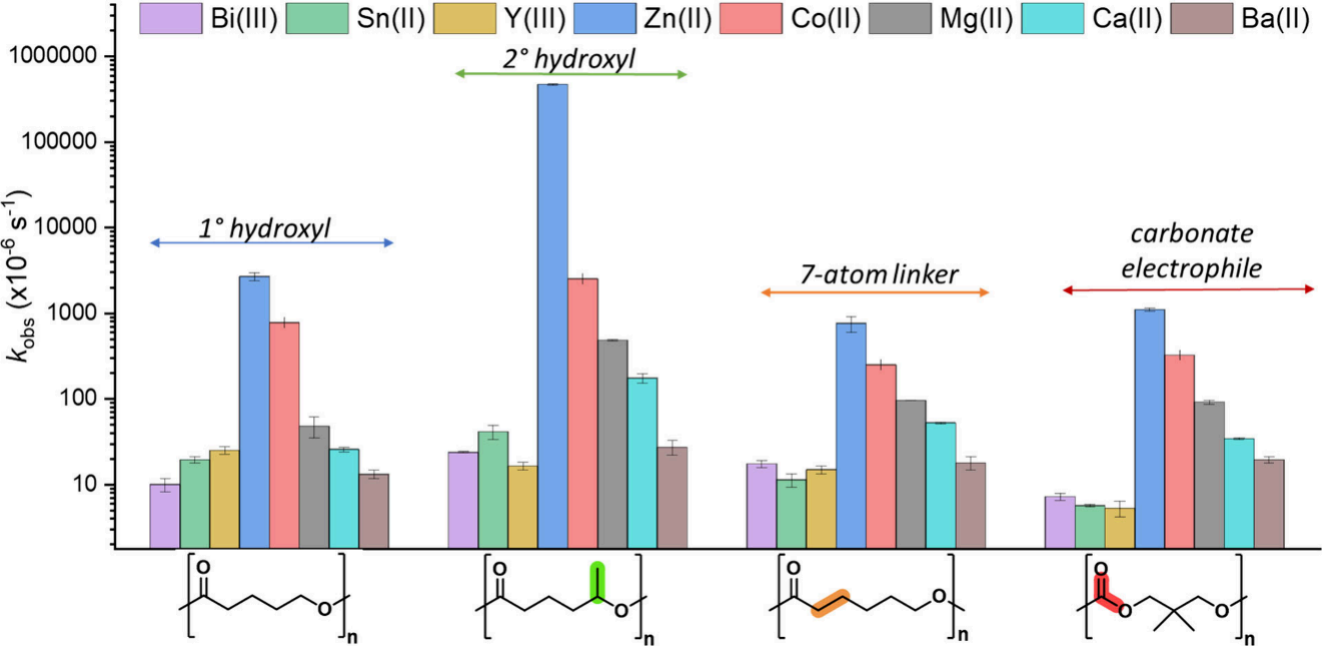
Plot showing rate constant taken from kinetic fit of conversion vs time data for the depolymerization of PVL, PHL, PCL and PDTC. To account for differences in rate of depolymerization for the different polymers, reactions were conducted at [catalyst]_0_:[polymer] loadings of 1:1000 and 130 °C for PVL, 1:100 and 150 °C for PHL, 1:100 and 160 °C for PCL and 1:1000 and 150 °C for PDTC. All reactions were conducted under an N_2_ flow of 25 mL min^–1^.

All the catalysts were highly selective for the formation of desired 6- or 7-lactone or cyclic carbonate product, i.e. all the chemical recycling processes formed the monomers in very high conversion selectivity. Lab-scale reactions conducted using each of the four polymers with the Zn­(II) catalyst (*ca* 1 g, 1:100 – 1000, 130 – 160 °C, under dynamic vacuum) resulted in monomer recovery in high yield and excellent purity, as shown by ^1^H NMR spectroscopy and GC-MS (82 – 99% yield, 99% selectivity, Figures S12–17). In every case, the results were fully consistent with the TGA-IR experiments, giving confidence in the predictive capability of the small-scale kinetics measurements. Across all four polymer samples, the Zn­(II) catalyst consistently and significantly outperformed all the other metals (Figure 3). For example, for polycaprolactone (PCL) depolymerization, the Zn­(II) catalyst exhibited a TOF of 200 h^–1^ (*k*_obs_ ≈ 0.75 × 10^–3^ s^–1^), which is double the rate of the next fastest Co­(II) catalyst (TOF = 90 h^–1^, *k*_obs_ = 0.025 h^–1^).

The polymer structure certainly influences the absolute depolymerization rate, but it does not influence the relative order of catalyst performances. For all polymers, the depolymerization rate order is (from fastest to slowest):

Zn(II)>Co(II)>Mg(II)>Ca(II)>Ba(II)

For the slower catalysts, rates are approximately the same for Sn­(II), Bi­(III) and Y­(III).

### Depolymerization Mechanism

The chemical recycling may occur either by a chain-end depolymerization mechanism, i.e., monomer release occurs sequentially from the polymer chain-end, or by random chain-scission reactions, i.e., monomer release occurs from any polymer ester or carbonate linkage. To investigate the possible mechanism, the chemical recycling of acetyl end-capped PVL (PVL-OAc) was tested using the Zn­(II), Co­(II) Mg­(II) and Sn­(II) catalysts (1:1000 M­(Oct)_2_, 130 °C, Figures S18–21). In all cases, the chemical recycling of PVL-OAc was significantly slower than PVL–OH, indeed it was always at least 50x slower. These results suggest that recycling occurs predominantly via a chain-end mechanism.

As mentioned, the Zn­(Oct)_2_, catalyst was previously used to determine a relationship between the polymer-to-monomer depolymerization equilibrium constant and the recycling rate.^29^ To understand whether such correlations are generally applicable to other catalysts, the depolymerization of a series of polyesters and carbonates was conducted using the Co­(II), Mg­(II) and Sn­(II) catalysts. In these experiments, each polymer was tested with the target catalyst under the same conditions: 130 °C, catalyst: polymer repeat unit = 1:1000. The polymers are poly­(3-methylvalerolactone) (P3 MeVL), polyvalerolactone (PVL), poly­(3,3-dimethyltrimethylenecarbonate) (PDTC) and poly­(4-methyl caprolactone) (P4MeCL) and polycaprolactone (PCL) (Figure 4). All polymers in this series are terminated with primary alcohol groups. Testing the entire series of polymers and catalysts reveals a clear exponential relationship between the depolymerization equilibrium and the catalytic rate of depolymerization (Figure 4a and 4b, Tables S8–10, Figures S22–24). Each catalyst shows the characteristic linear free energy relationship between the thermodynamic and kinetic parameters (Figure 4a). This means that the more favorable the depolymerization thermodynamics, i.e., the higher the K_d_ value, the faster the recycling rate. It is notable that these relationships hold in all cases even though the values for the recycling thermodynamic equilibrium constants span 3 orders of magnitude and rate constants span 3–4 orders of magnitude. Although the absolute rates are, of course, catalyst dependent, the relative depolymerization rates are not; all catalysts follow the order (from fastest to slowest):

P3MeVL>PVL>PDTC>P4MeCL>PCL

**Figure 4:**
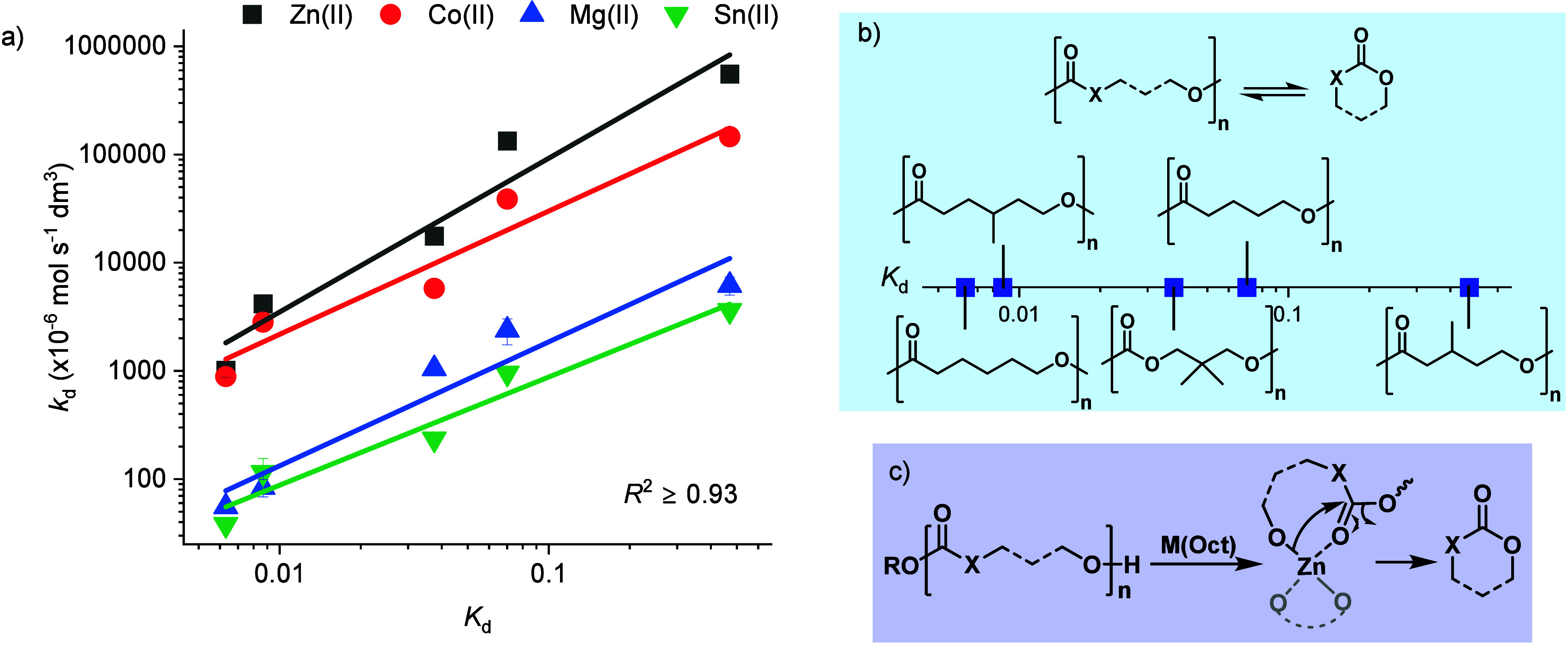
(a) Plots showing the depolymerization rate constant, *k*_d_ vs depolymerization equilibrium constant, *K*_d_ for the five different polymer samples, with distinctive exponential correlations for the Zn­(II), Co­(II), Mg­(II) and Sn­(II) catalysts (note both x- and *y*-axes are plotted on logarithmic scales). Depolymerization rate coefficient, *k*_d_ = *k*_obs_/(2­[cat]_0_), assuming 2 active chains per metal center. Recycling catalysis conditions: [catalyst]_0_:[polymer]_0_ 1:1000, 130 °C, N_2_ flow = 25 mL min^–1^. (b) Scheme showing the depolymerization equilibrium constant, *K*_d_, with the structures for P3MeVL, PVL, PDTC, P4MeCL and PCL. (c) Proposed chain-end mechanism for the depolymerization of the polymers to their cyclic monomers.

Thus, for all the catalysts at 130 °C, PCL, for which depolymerization thermodynamics are disfavored (equilibrium conversion in a closed system would be <1%), exhibits ∼ 25–100 fold slower rates of recycling, under non-equilibrium conditions, than for PVL. It is worth comment that linear free energy relationships apply even to reactions which are driven, i.e. the depolymerizations are not conducted under equilibrium but rather with continual product removal so as to ensure very high conversion efficiency. The catalyst structure-performance data and the end-capping experiments suggest that all these catalysts operate by chain-end depolymerization mechanisms.

The proposed depolymerization mechanism involves a number of steps. It is initiated by the M­(Oct)_2_ catalyst reacting with the hydroxyl polymer chain end groups to form the active metal-alkoxide catalyst species (Figure 4c). This type of initiation is directly analogous to that which has been extensively studied for Sn­(Oct)_2_-catalyzed lactone and lactide polymerizations (and PLLA recycling catalysis).^13,30^ Next, the metal-alkoxide species coordinates an adjacent in-chain carbonyl group, activating it to nucleophilic attack. The metal alkoxide reacts with the activated ester or carbonate carbonyl functionality, by intramolecular transesterification, to form the lactone (or cyclic carbonate) and reform a new chain-shortened metal alkoxide intermediate (Figure 4c).

### Recycling Catalysis: Linear Free Energy Relationships

The proposed recycling mechanism suggests that the catalytic activity should be controlled by two factors: 1) The catalyst metal-alkoxide nucleophilicity and 2) The catalyst metal activation of the in-chain carbonyl group for the intramolecular transesterification. These factors, and the proposed mechanism for polymerization catalysis, suggest that the metal center Lewis acidity may be important in controlling activity. Therefore, quantified catalyst structure-performance relationships should test the appropriate descriptors of metal acidity. However, although there are a range of methods to indirectly measure metal Lewis acidity, there are not any common Lewis acidity scales applicable to all of the metals investigated in this work. Bronsted acidity values, of course, follow a common scale. These polymerizations are conducted in nonaqueous environments but others have shown a linear correlation between Bronsted and Lewis acidity values, with the latter determined in organic solvents by NMR titration methods.^31^ Further, others have suggested that metal­(aqua complex) hydrolysis constants, *p*K_h_ (see eqs S2 and S4 for full definition) can serve as indirect measures for Lewis acidity values.^32,33^ Indeed, it has been shown that catalytic activity correlates with metal center Lewis acidity (as assessed by *pK*_h_ values) in reactions as diverse as electrocatalysis,^32,34–37^ organic transformations, such as the Mukaiyama aldol reaction,^38^ and, for various oxygenated monomer polymerizations.^39,40^ Thus, for every polymer, plots were constructed of the depolymerization rate constant (*k*_obs_) vs the metal Lewis acidity (*p*K_h_, assuming monomeric metal catalyst speciation under high dilution conditions). These plots all showed very clear exponential correlations with volcano-type relationships (Figures 5, S27–S29). The key finding is that the best metals are those with intermediate Lewis acidity (*p*K_h_) values, such as Zn­(II), Co­(II) and Mg­(II) (Figure 5a). Plots of catalytic activity against other descriptors, including metal ionic radius or metal center oxophilicity, did not show any correlations (Figures S25 and S26). Thus, in this recycling chemistry, metal acidity values are effective descriptors of catalytic performance. All the polymers investigated show the same types of linear free energy relationships, with rates of depolymerization correlating exponentially with metal acidity values (Figure 5b, S27–S29). It is very interesting that the rate-structure correlation is so general: it applies over a very wide range of magnitudes in reaction rate, over a range of catalyst loadings and to all polymer chain linker lengths, to different polymer backbone chemistries, and to substituted/unsubstituted polymer backbones (Figures 5b, S27–S29). One benefit of these plots is their utility in understanding the most appropriate conditions for recycling of a particular polymer. Another benefit lies in the insight they provide into the recycling mechanism. A third attraction is to use them to guide future catalyst design for even better performances. For instance, to improve Mg­(II) recycling catalysts, ligand design should focus on increasing the Mg­(II) Lewis acidity (*p*K_h_) compared to that obtained for the 2-ethylhexanoate complex.

**Figure 5:**
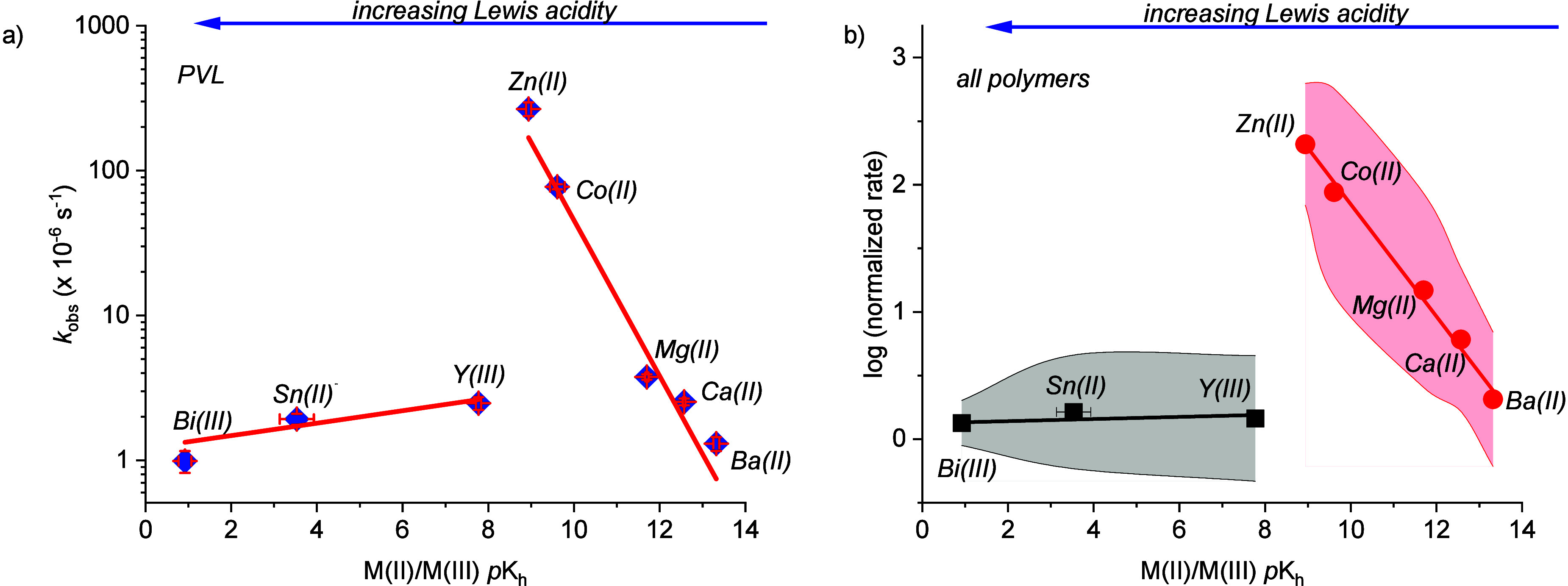
(a) Plot of the recycling rate constant, *k*_*obs*_, vs metal center acidity, p*K*_h_, for the depolymerization of PVL. Recycling conditions: [catalyst]_0_:[PVL]_0_ 1:1000, 130 °C, N_2_ flow= 25 mL min^–1^. (b) Plot of log (normalized rate) vs metal cenre acidity, p*K*_h_, for the series of catalysts and for all polymers (for individual plots, see Figures S27–29). To account for differences in the rate of depolymerization, conditions were varied: For PVL, [catalyst]_0_:[polymer] 1:1000, 130 °C; PHL = 1:100, 150 °C; PCL = 1:100, 160 °C; PDTC = 1:1000, 150 °C. All reactions were conducted under an N_2_ flow of 25 mL min^–1^. The rate was normalized by dividing the absolute rate of the catalyst by the minimum rate obtained for that polymer series.

### Recycling Catalysis Eyring Analysis

To further understand how varying the metal Lewis acidity (*p*K_h_) influences the depolymerization rate, Eyring analysis was performed. The analysis was conducted for the recycling of polyvalerolactone, PVL, with the three fastest metal catalysts, Zn­(II), Co­(II) and Mg­(II), and also with Sn­(II), which represents a metal­(II) center on the other side of the ‘peak’ in the plot (Figure 5a). The recycling conditions were catalyst:polymer loadings of 1:1000 and rates were measured at systematically varied temperatures from 90 to 170 °C. As expected, PVL depolymerization rates increased with temperature for all catalysts (Tables S11–14, Figures S30–35). Plots of the ln (*k*_d_/T) vs reciprocal temperature (1/T) allowed for determination of the depolymerization transition-state enthalpy (gradient, Δ*H*_d_^‡^), entropy (intercept, Δ*S*_d_^‡^) and Gibbs Free Energy (Δ*G*_d_^‡^). In all cases, the recycling transition state enthalpy barriers, Δ*H*_d_^‡^, are positive, and the entropy barriers, Δ*S*_d_^‡^, are negative. This aligns with the mechanism where the addition of the metal-alkoxide intermediate to the activated carbonyl group requires bond breaking and formation, i.e., incurring an enthalpy penalty. In accessing the cyclic transition state, the polymer chain must adopt a specific conformation which will result in an entropy cost (Δ*S*_d_^‡^ < 0). Careful analysis of the transition state enthalpy and entropy values reveals that metal Lewis acidity values influence the relative weighting of the enthalpy and entropy barriers (Figure 6). It is instructive to consider how decreasing the metal Lewis acidity (i.e., increasing metal p*K*_h_ values) from the most Lewis acidic Sn­(II) catalyst to the least acidic Mg­(II) affects the relative transition state parameters. Moving from the most Lewis-acidic Sn­(II) to the least acidic Mg­(II) (i.e., increasing p*K*_h_) led to a progressive rise in the activation enthalpy, Δ*H*_d_^‡^, and reduction in the activation entropy, Δ*S*_d_^‡^. Remarkably, the transition state activation parameters, Δ*H*_d_^‡^ and Δ*S*_d_^‡^, both exhibit a strong, inverse linear correlation with metal *p*K_h_ values. This means that increasing the metal p*K*_h_ value results in a linear progressive increase in Δ*H*_d_^‡^ and decrease in Δ*S*_d_^‡^ (Figure 6a–c).

**Figure 6:**
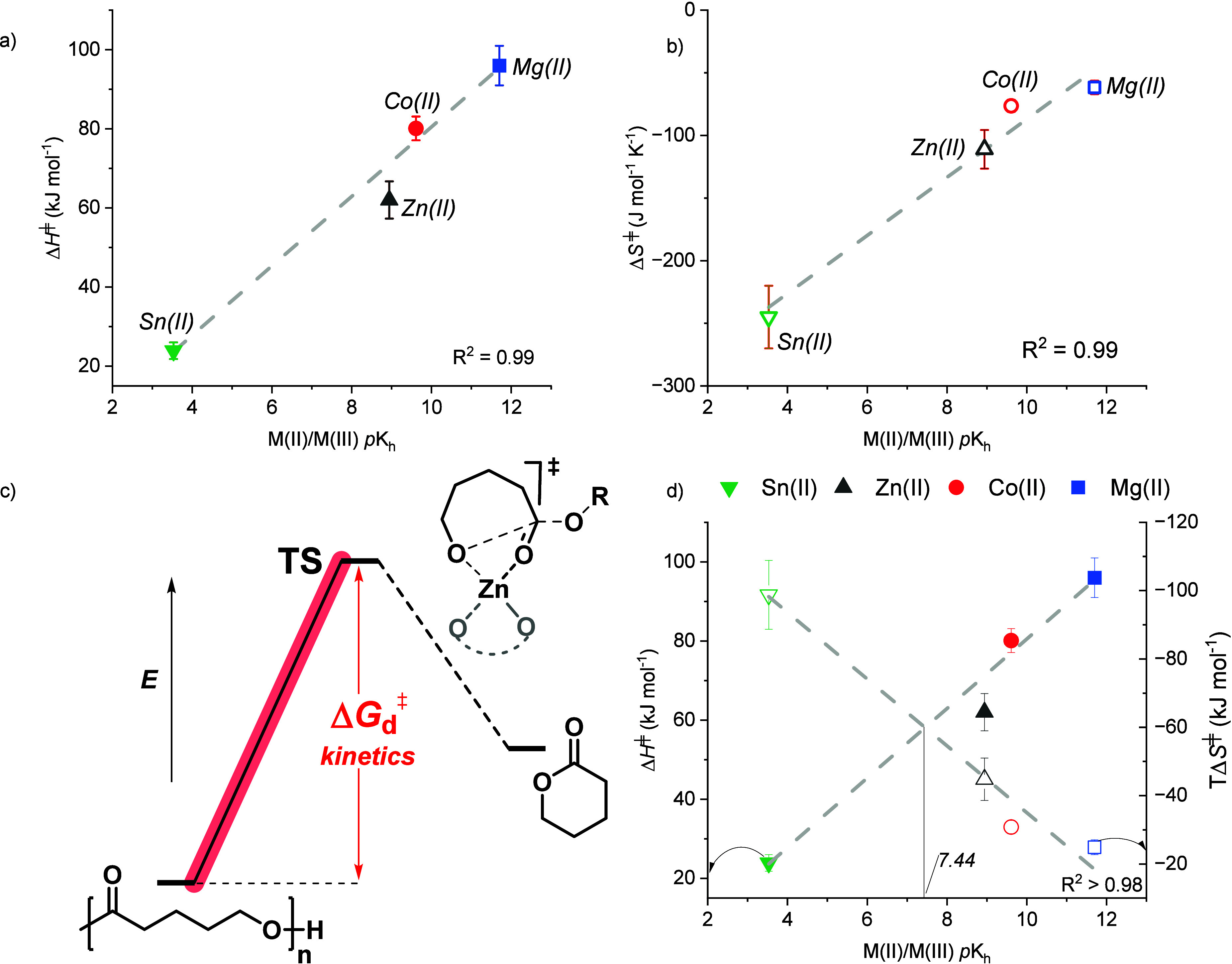
Transition-state activation enthalpy (Δ*H* ^‡^), entropy (Δ*S* ^‡^) and illustrated transition state for the depolymerization of PVL. Reaction conditions:[catalyst]_0_:[polymer]_0_ loadings of 1:1000, at temperatures between 90 and 170 °C, for Zn­(II), Co­(II), Mg­(II) and Sn­(II), with N_2_ flow rates of 25 mL min^–1^. (a) Plot of Δ*H* ^‡^ vs metal ion *p*K_h_ for the depolymerization of PVL. (b) Plot of Δ*S* ^‡^ vs metal *p*K_h_ for the depolymerization of PVL. (c) Schematic illustrating proposed transition state. (d) Plots of Δ*H* ^‡^ and TΔ*S* ^‡^ for the depolymerization of PVL vs *p*K_h_ of the metal. Filled symbols represent Δ*H* ^‡^ values (left *y* axis), and hollow symbols represent TΔ*S* ^‡^ values (right *y* axis).

Thus, the outstanding performance of Zn­(II) can be rationalized: it balances the competing enthalpy and entropy contributions to the depolymerization transition state. In many respects, Zn­(II) is an excellent metal for depolymerization catalysis given its high abundance (24th most abundant element in the Earth’s crust),^41^ generally moderate-to-low toxicity,^42^ lightweight, and the tendency for its complexes to show thermal and redox stability. Nevertheless, the intersection of the Δ*H*_d_^‡^ and TΔ*S*_d_^‡^ vs *p*K_h_ plot (*p*K_h_ = 7.44, Figure 6d) provides insight into how, in future, Zn­(II) catalysts could be further optimized, for instance by exploiting ligand and complex design strategies to slightly increase its Lewis acidity.

### Computational Modeling

To gain further insight into the role of the metal in the depolymerization transition state, density functional theory (DFT) modeling of the intramolecular transesterification reaction to form the monomer was carried out. Both the Mg­(II) and Zn­(II) catalyzed reactions were calculated and compared. Based on the end-capping experiments, which show that the metal alkoxide is the key reactive intermediate in depolymerization, and the solid-state structure of the metal 2-ethylhexanoate,^43^ the active catalyst species was modeled as a tetrahedral metal alkoxide. The intramolecular transesterification mechanism was modeled as a two-step addition elimination process (Figure 7, Table S15). First, the metal-alkoxide undergoes an addition at the carbonyl group coordinated to the metal center (TS1) to form an acetal intermediate. Next, the acetal intermediate rearranges by M-O acyl bond cleavage (TS2) to form the monomer-bound catalyst. In agreement with experiments, where Zn­(II) is the fastest catalyst, the computational modeling shows that the Zn­(II) alkoxide has an overall lower barrier than the Mg­(II) complex. The lower overall transition state energy results from the lower barrier for TS1 for Zn­(II) as compared with that for Mg­(II). For Zn­(II), the transition states (TS1 and TS2) are almost the same within error, suggesting that the rate-determining step has character of both the addition and elimination steps.

**Figure 7:**
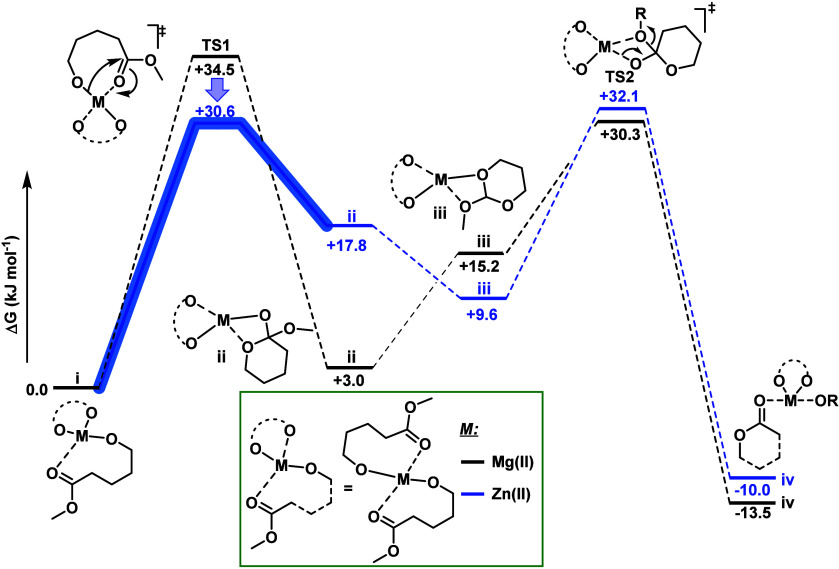
Computed pathway for the ring-closing of 5-hydroxypentanone catalyzed by Zn­(II) and Mg­(II) alkoxide complexes. Calculations were performed using the PBE0 functional (with empirical dispersion correction factor, GD3, applied), basis set 6–31+G­(d,p) for O atoms, 6–31G­(d,p) for C and H atoms, and basis set and pseudo potential SDD for Zn and Mg atoms. Solvent effects were accounted for using the SMD continuum model for ethyl acetate. Temperature and concentration were corrected by using *Goodvibes* software.

On the other hand, for the Mg­(II) catalyst, TS1, the addition of the alkoxide to the carbonyl, is likely to be the rate-determining step. This notion is consistent with the Eyring analysis and experimental data, which suggests that the Mg­(II) catalyst incurs a lower entropy penalty in accessing its transition state than the Zn­(II) catalyst. The computational modeling suggests that for Mg­(II) there are no conformational changes (intermediates) between the initial state of the catalyst (i) and the rate determining transition state (TS1). On the other hand, for Zn­(II) there are two intermediates (ii), (iii) between the initial state (i) and the highest-energy transition state (TS2). Overall, the computational modeling suggests that the Zn­(II) catalyst is more effective because it activates the carbonyl group to intramolecular cyclization, reducing the cyclization enthalpy penalty and enabling faster depolymerization rates. Overall, the computational modeling results are consistent with the experimental data.

## Conclusion

In summary, a series of Lewis acidic metal catalysts showed very high selectivity and variable rates for chemical recycling by depolymerization of polyesters and polycarbonates to cyclic monomers. The recycling reactions were all conducted using neat polymer, at low catalyst loadings (1:100–1:1000), and at low temperatures (90–170 °C). The Zn­(II) catalyst showed the fastest rates and was more than 1000 times faster than the slowest catalysts in the series. After Zn­(II), the next best catalysts were based Co­(II) and Mg­(II). A useful catalyst structure-performance correlation was revealed between the metal Lewis acidity, as assessed by its hydrolysis constant, and the rate of depolymerization. The linear free energy relationship applies to a range of different polymers and to all of the catalysts. Eyring analysis, conducted using the lead catalysts Zn­(II), Co­(II), Mg­(II) and Sn­(II), suggested that Zn­(II) is the most effective metal in being able to balance competing entropy and enthalpy transition-state barriers to depolymerization. Overall, the catalyst structure-performance relationship, or linear free energy relationship, provides a useful conceptual framework for future investigations into both catalyst and process design for recycling. It also allows for rational selection of both the polymer and catalyst to deliver the fastest and lowest energy recycling.

## Supplementary Material



## Notes

The experimental data and computational outputs can be found online at Oxford University Research Archive, ORA, DOI: 10.5287/ora-aqa4e6xvr, https://dx.doi.org/10.5287/ora-aqa4e6xvr. The Supporting Information is available free of charge at https://pubs.acs.org/doi/10.1021/jacs.5c16346.

Experimental Section including Materials and Methods, Supplementary Text, TGA kinetic data for depolymerization, polymer purity evaluation by ^1^H NMR spectroscopy, SEC, DSC and TGA, monomer purity evaluation by ^1^H NMR spectroscopy and GC-MS, computational details and Gibbs free energies (PDF)
