# Supplementary material for: Chemical Recycling of Polyesters and Polycarbonates: Why Is Zinc(II) Such an Effective Depolymerization Catalyst?
Source: J Am Chem Soc. 2025 Nov 10;147(46):43077–85. doi: 10.1021/jacs.5c16346 (PMC12636022; doi:10.1021/jacs.5c16346)
Supplement: Supplementary file 1 [file ja5c16346_si_001.pdf]

Supporting information: Chemical Recycling of Polyesters and Polycarbonates: Why is Zinc(II) such an Effective Depolymerization Catalyst?

Thomas M. McGuire,*[a] Antoine Buchard*[b] and Charlotte K. Williams*[a]

[a] Department of Chemistry, Chemistry Research Laboratory, University of Oxford, 12 Mansfield Rd, Oxford, OX1 3TA, U.K

[b] Department of Chemistry, Green Chemistry Centre of Excellence, University of York YO10 5DD, U. K.

Corresponding Authors

Charlotte K. Williams: Email: charlotte.williams@chem.ox.ac.uk

Thomas M. McGuire: Email: thomas.mcguire@chem.ox.ac.uk

Antoine Buchard: Email: antoine.buchard@york.ac.uk

Table of Contents

Supporting information: Chemical Recycling of Polyesters and Polycarbonates: Why is Zinc(II) such an Effective Depolymerization Catalyst?.. 1

1. Materials.. 7

2. Methods 8

2.1 Polymerization of monomers using diphenyl phosphate catalyst 9

2.2 Polymerization of monomers using 1,5,7-Triazabicyclo[4.4.0]dec-5-ene catalyst 9

2.3 Kinetics of Solid-State Polymer Chemical Recycling to Monomer 9

Figure S1. Example data for recycling of polyesters and polycarbonates showing conversion vs time (black), measured rate (blue), kinetic fit ($y = a1 + e - kx - xc$, red curve), a and x_c parameters. 10

2.4 Procedure for end-capping PVL 11

3. Monomer, polymer and catalyst properties 12

Table S1. Catalyst properties... 12

Table S2. Polymerization and Depolymerization Thermodynamic Parameters for Bulk Polymerization 12

Table S3. Polymer Synthesis Data 13

4. Additional information 14

4.1 Recycling Catalyst Data.. 14

Table S4. Depolymerization Kinetic Data for PVL at 130 °C with 1:1000 $[M(Oct)_n]_0$:$[PVL]_0$ loading... 14

Figure S2. Plots of PVL conversion *vs* time and kinetic fits. Experimental conditions: 1: 1000 $M(Oct)_n$:PVL, 130 °C, for 5 h or until >95% conversion.. 15

Figure S3. IR spectra showing formation of VL in TGA-IR experiments of $M(Oct)_n$:PVL mixtures. Experimental conditions: 1: 1000 $M(Oct)_n$:PVL, heating rate of 10 °C min^{-1}........... 15

Figure S4. ^{1}H NMR spectrum (400 MHz, $CDCl_3$, 298K) of VL isolated from recycling of PVL ($[Zn(Oct)_2]_0$:$[PVL]_0$ 1:1000, 130 °C). Reproduced from[5] 16

Figure S5. GC of of VL isolated from recycling of PVL ($[Zn(Oct)_2]_0$:$[PVL]_0$ 1:1000, 130 °C); pure sample of VL elutes at 7.87 minutes. Reproduced from [14] 17

Table S5. Depolymerization Kinetic Data for PCL at 160 °C with 1:100 $[M(Oct)_n]_0$: $[PCL]_0$ loading... 18

Figure S6. Plots of PCL conversion *vs* time and kinetic fits. Experimental conditions: 1: 100 $M(Oct)_n$:PCL, 160 °C, for 5 h or until >95% conversion.. 19

Figure S7. IR spectra showing formation of CL in TGA-IR experiments of $M(Oct)_n$:PCL mixtures. Experimental conditions: 1: 100 $M(Oct)_n$:PCL, heating rate of 10 °C min^{-1}........................... 19

Table S6. Depolymerization Kinetic Data for PHL at 150 °C with 1:100 $[M(Oct)_n]_0$: $[PHL]_0$ loading... 20

Figure S8. Plots of PHL conversion *vs* time and kinetic fits. Experimental conditions: 1: 100 $M(Oct)_n$:PHL, 150 °C, for 5 h or until >95% conversion 20

Figure S9. IR spectra showing formation of HL in TGA-IR experiments of $M(Oct)_n$:PHL mixtures (1:100). Experimental conditions: 1: 100 $M(Oct)_n$:PHL, heating rate of 10 °C min^{-1}..... 21

Table S7. Depolymerization Kinetic Data for PDTC at 150 °C with 1:1000 $[M(Oct)_n]_0$: $[PDTC]_0$ loading..... 21

Figure S10. Plots of PDTC conversion *vs* time and kinetic fits. Experimental conditions: 1: 1000 $M(Oct)_n$:PDTC, 150 °C, for 5 h or until >95% conversion 22

Figure S11. IR spectra showing formation of DTC in TGA-IR experiments of $M(Oct)_n$:PDTC mixtures. Experimental conditions: 1: 1000 $M(Oct)_n$:PDTC, heating rate of 10 °C min^{-1} 22

Figure S12. ^1H NMR spectrum (400 MHz, $CDCl_3$, 298K) of CL isolated from recycling of PCL ($[Zn(Oct)_2]_0$:$[PCL]_0$ 1:100, 160 °C). Reproduced from[5] 23

Figure S13. GC of CL isolated from recycling of PCL ($[Zn(Oct)_2]_0$:$[PCL]_0$ 1:100, 160 °C). Reproduced from[5] 24

Figure S14. ^1H NMR spectrum (400 MHz, $CDCl_3$, 298K) of HL isolated from recycling of PHL ($[Zn(Oct)_2]_0$:$[PHL]_0$ 1:100, 160 °C). Reproduced from[5] 25

Figure S15. GC of HL isolated from recycling of of PHL ($[Zn(Oct)_2]_0$:$[PHL]_0$ 1:100, 160 °C). Pure sample of HL elutes at 8.48 minutes. Reproduced from[5] 26

Figure S16. ^1H NMR spectrum (400 MHz, $CDCl_3$, 298K) of DTC isolated from recycling of PDTC ($[Zn(Oct)_2]_0$:$[PDTC]_0$ 1:100, 160 °C). Reproduced from[5] 27

Figure S17. GC of DTC isolated from recycling of PDTC ($[Zn(Oct)_2]_0$:$[DTC]_0$ 1:100, 160 °C). Reproduced from[5] 28

4.2 Investigation of the PVL Recycling Mechanism using $Zn(Oct)_2$, $Co(Oct)_2$, $Mg(Oct)_2$ and $Sn(Oct)_2$ Catalysts at 130 °C 29

Figure S18. Plots of PVL conversion *vs* time for hydroxyl-end capped PVL (PVL-OH) and acetyl-end capped PVL (PVL-OAc) catalysed by $Zn(Oct)_2$. Experimental conditions: 1: 1000 $Zn(Oct)_2$: PVL, 130 °C, for 5 h or until >95% conversion 29

Figure S19. Plots of PVL conversion *vs* time for hydroxyl-end capped PVL (PVL-OH) and acetyl-end capped PVL (PVL-OAc) catalysed by $Co(Oct)_2$. Experimental conditions: 1: 1000 $Co(Oct)_2$: PVL, 130 °C, for 5 h or until >95% conversion 30

Figure S20. Plots of PVL conversion *vs* time for hydroxyl-end capped PVL (PVL-OH) and acetyl-end capped PVL (PVL-OAc) catalysed by $Mg(Oct)_2$.. Experimental conditions: 1: 1000 $Mg(Oct)_2$: PVL, 150 °C, for 5 h or until >95% conversion 30

Figure S21. Plots of PVL conversion *vs* time for hydroxyl-end capped PVL (PVL-OH) and acetyl-end capped PVL (PVL-OAc) catalysed by $Sn(Oct)_2$. Experimental conditions: 1: 1000 $Sn(Oct)_2$: PVL, 150 °C, for 5 h or until >95% conversion 31

Table S8. Depolymerization Kinetic Data for P3MeVL, PVL, PDTC, P4MeCl and PCL at 130 °C with 1:1000 $[Co(Oct)_2]_0$: [polymer repeat unit]$_0$ loading 32

Figure S22. Plots of polymer conversion *vs* time for P3MeVL, PVL, PDTC, P4MeCL and PCL depolymerization catalysed by $Co(Oct)_2$. Experimental conditions: 1: 1000 $Co(Oct)_2$:PVL, 130 °C, for 10 h or until >95% conversion 32

Table S9. Depolymerization Kinetic Data for P3MeVL, PVL, PDTC, P4MeCL and PCL at 130 °C with 1:1000 [Mg(Oct)$_2$]$_0$: [polymer repeat unit]$_0$ loading 33

Figure S23. Plots of polymer conversion *vs* time for P3MeVL, PVL, PDTC, P4MeCL and PCL depolymerization catalysed by Mg(Oct)$_2$. Experimental conditions: 1: 1000 Mg(Oct)$_2$: PVL, 130 °C, for 5 h or until >95% conversion 33

Table S10. Depolymerization Kinetic Data for P3MeVL, PVL, PDTC, P4MeCl and PCL at 130 °C with 1:1000 [Sn(Oct)$_2$]$_0$: [polymer repeat unit]$_0$ loading.. 34

Figure S24. Plots of polymer conversion *vs* time for P3MeVL, PVL, PDTC, P4MeCL and PCL depolymerization catalysed by Sn(Oct)$_2$. Experimental conditions: 1: 1000 Sn(Oct)$_2$: PVL, 130 °C, for 5 h or until >95% conversion 34

Figure S25. Plot showing k_{obs} vs metal ionic radius (coordination number 6) for the depolymerization of PVL catalysed by M(Oct)$_n$. Experimental conditions: M(Oct)$_n$: PVL, 1:1000, 130 °C for 5 h or until >95% conversion. Ionic radii were taken from[7, 8] 35

Figure S26. Plot showing k_{obs} vs metal oxophilicity for the depolymerization of PVL catalysed by M(Oct)$_n$. Experimental conditions: M(Oct)$_n$: PVL, 1:1000, 130 °C for 5 h or until >95% conversion. Oxophilicity values taken from[9] 35

Figure S27. Plot showing k_{obs} vs metal ion pK_h for the depolymerization of PHL catalysed by M(Oct)$_n$. Experimental conditions: M(Oct)$_n$: PHL, 1:100, 150 °C for 5 h or until >95% conversion. pK_h values taken from[4] 36

Figure S28. Plot showing k_{obs} vs metal ion pK_h for the depolymerization of PCL catalysed by M(Oct)$_n$. Experimental conditions: M(Oct)$_n$: PCL, 1:100, 160 °C for 5 h or until >95% conversion. pK_h values taken from[4] 36

Figure S29. Plot showing k_{obs} vs metal ion pK_h for the depolymerization of PDTC catalysed by M(Oct)$_n$. Experimental conditions: M(Oct)$_n$: PDTC, 1:100, 150 °C for 5 h or until >95% conversion. pK_h values taken from[4] 37

4.3 Eyring analysis 38

Table S11. Depolymerization Kinetic Data for PVL at 100 – 140 °C with 1:1000 [Co(Oct)$_2$]$_0$: [PVL]$_0$ loading.. 38

Figure S30. Plots of polymer conversion *vs* time and kinetic fits for PVL depolymerization catalysed by Co(Oct)$_2$ at 100, 110, 120, 130 and 140 °C. Experimental conditions: 1: 1000 Co(Oct)$_2$:PVL, for 5 h or until >95% conversion 38

Figure S31. Plot of ln (k_d/T) *vs* 1/T for the recycling of PVL. Reactions were performed at 100, 110, 120, 130 and 140 °C with loadings of [Co(Oct)$_2$]$_0$: [PVL]$_0$ of 1:1000. [Co(Oct)$_2$]$_0$ = 9.98 x10^{-3}M 39

Table S12. Depolymerization Kinetic Data for PVL at 130 – 170 °C with 1:1000 [Mg(Oct)$_2$]$_0$: [PVL]$_0$ loading.. 40

Figure S32. Plots of polymer conversion *vs* time and kinetic fits for PVL depolymerization catalysed by Mg(Oct)$_2$ at 100, 110, 120, 130 and 140 °C. Experimental conditions: 1: 1000 Mg(Oct)$_2$:PVL, for 5 h or until >95% conversion. 41

Figure S33. Plot of ln (k_d/T) *vs* 1/T for the recycling of PVL. Reactions were performed at 130, 140, 150, 160 and 170 °C with loadings of [Mg(Oct)$_2$]$_0$: [PVL]$_0$ of 1:1000. [Mg(Oct)$_2$]$_0$ = 9.98 x10^{-3} M.. 41

Table S13. Depolymerization Kinetic Data for PVL at 130 – 170 °C with 1:1000 $[Sn(Oct)_2]_0$: $[PVL]_0$ loading 42

Figure S34. Plots of polymer conversion *vs* time and kinetic fits for PVL depolymerization catalysed by $Sn(Oct)_2$ at 100, 110, 120, 130 and 140 °C. Experimental conditions: 1: 1000 $Sn(Oct)_2$:PVL, for 5 h or until >95% conversion 43

Figure S35. Plot of ln (k_d/T) *vs* 1/T for the recycling of PVL. Reactions were performed at 130, 140, 150, 160 and 170 °C with loadings of $[Sn(Oct)_2]_0$: $[PVL]_0$ of 1:1000. $[Sn(Oct)_2]_0 = 9.98 \times 10^{-3}$ M 43

Table S14. Summary of Eyring data for depolymerization of PVL using loadings of 1:1000 $[M(Oct)_n]_0$: $[PVL]_0$ 44

4.4 DFT 45

Table S15. Computed intermediates and transition states for the backbiting of methyl 5-hydroxypentanoate and methyl 6-hydroxyhexanoate 45

5. Polymer and large-scale recycled monomer characterisation 46

Figure S36. SEC trace of PVL. $M_{n,SEC}$ = 17,500 g mol $^{-1}$, $Đ_M$ = 1.09 47

Figure S37. SEC trace of P3MeVL. $M_{n,SEC}$ = 23,100 g mol $^{-1}$, $Đ_M$ = 1.10 47

Figure S38. SEC trace of PHL. $M_{n,SEC}$ = 21,000 g mol $^{-1}$, $Đ_M$ = 1.10 48

Figure S39. SEC trace of PCL. $M_{n,SEC}$ = 24,900 g mol $^{-1}$, $Đ_M$ = 1.39 48

Figure S40. SEC trace of P4MeCL. $M_{n,SEC}$ = 22,800 g mol $^{-1}$, $Đ_M$ = 1.39 49

Figure S41. SEC trace of PDTC. $M_{n,SEC}$ = 22,200 g mol^{-1}, $Đ_M$ = 1.20 49

Figure S42. ^{1}H NMR spectrum (400 MHz, $CDCl_3$, 298K) of PVL. DP_{NMR} =104 determined from relative integration of H_d and H_e 50

Figure S43. ^{1}H NMR spectrum (400 MHz, $CDCl_3$, 298K) of P3MeVL. DP_{NMR} =125 determined from relative integration of H_d and H_f 51

Figure S44. ^{1}H NMR spectrum (400 MHz, $CDCl_3$, 298K) of PHL. DP_{NMR} =106 determined from relative integration of H_d and H_f 52

Figure S45. ^{1}H NMR spectrum (400 MHz, $CDCl_3$, 298K) of PCL. DP_{NMR} =112 determined from relative integration of H_f and H_e 53

Figure S46. ^{1}H NMR spectrum (400 MHz, $CDCl_3$, 298K) of P4MeCL. DP_{NMR} =111 determined from the relative integral of H_e and H_g 54

Figure S47. ^{1}H NMR spectrum (400 MHz, $CDCl_3$, 298K) of PDTC. DP_{NMR} =108 determined from relative integration of H_c and H_a 55

Figure S48. DSC thermograms (2nd heating cycle) of PVL T_g = -55 °C, T_m = 53 °C 56

Figure S49. DSC thermogram (2nd heating cycle) of P3MeVL, T_g = -54 °C, T_m = *not observed* . 57

Figure S50. DSC thermograms of PHL, T_g = -61 °C, T_m (2nd heating cycle) = *not observed.* T_m (first cycle) = 54 °C. 58

Figure S51. DSC thermograms (2nd heating cycle) of PCL, T_g = -61 °C, T_m = 53 °C 59

Figure S52. DSC thermogram (2nd heating cycle) of P4MeCL, T_g = -61 °C, T_m =*not observed* ... 60

Figure S53. DSC thermograms (2nd heating cycle) of PDTC, T_g = 28 °C, T_m = 106 °C 61

Figure S54. TGA thermogram of PVL, $T_{d,onset}$ = 324 °C 62

Figure S55. TGA thermogram of P3MeVL, $T_{d,onset}$ = 320 °C 62

Figure S56. TGA thermogram of PHL, $T_{d,onset}$ = 315 °C 63

Figure S57. TGA thermogram of PCL, $T_{d,onset}$ = 383 °C 63

Figure S58. TGA thermogram of P4MeCL, $T_{d,onset}$ = 373 °C 64

Figure S59. TGA thermogram of PDTC. $T_{d,onset}$ = 227 °C 64

Figure S60. ^{1}H NMR spectrum (400 MHz, $CDCl_3$, 298K) of 3MeVL isolated from recycling of P3MeVL ($[Zn(Oct)_2]_0$:$[P3MeVL]_0$ 1:1000, 130 °C) 65

Figure S61. GC of of 3MeVL isolated from recycling of P3MeVL ($[Zn(Oct)_2]_0$:$[P3MeVL]_0$ 1:1000, 130 °C). Pure sample of 3MeVL elutes at 8.63 minutes........ 66

Figure S62. ^{1}H NMR spectrum (400 MHz, $CDCl_3$, 298K) of 4MeCL isolated from recycling of P4MeCL ($[Zn(Oct)_2]_0$:$[P4MeCL]_0$ 1:100, 160 °C). Residual THF at δ = 3.75 ppm 67

Figure S63. GC of 4MeCL isolated from recycling of P4MeCL ($[Zn(Oct)_2]_0$:$[4MeCL]_0$ 1:100, 160 °C) 68

6. References 68

1. Materials

All raw experimental data and computational outputs can be found online at Oxford University Research Archive (ORA, DOI: 10.5287/ora-aqa4e6xvr, https://dx.doi.org/10.5287/ora-aqa4e6xvr)

All experiments were carried out under N_2 using standard Schlenk/glovebox techniques unless otherwise stated. Anhydrous dichloromethane was purchased from Sigma-Aldrich and degassed by N_2 purge prior to use. THF and toluene were obtained from an SPS system, degassed by several freeze-pump-thaw cycles and stored over 3 Å molecular sieves under nitrogen. Bi(III) 2-ethylhexanoate was purchased from Thermo Fisher Scientific. Y(III) 2-ethylhexanoate was purchased from Thermo Fisher Scientific. Tin(II) 2-ethylhexanoate ($Sn(Oct)_2$) was purchased from Sigma-Aldrich. Zinc(II) 2-ethylhexanoate was purchased from Fluorochem. Co(II) 2-ethylhexanoate was purchased from Sigma-Aldrich. Mg(II) 2-ethylhexanoate was purchased from Strem Chemicals. Ca(II) 2-ethylhexanoate was purchased from Alfa-Aesar. Ba(II) 2-ethylhexanoate was purchased from Biosynth Litd. All 2-ethylhexanoate catalysts were stored in a glovebox. 1,4-benzenedimethanol (BDM) was purchased from Sigma Aldrich. Prior to use, BDM was recrystallised from anhydrous toluene. 1,5,7-Triazabicyclodecene (TBD) was purchased from Caymen Island Chemicals stored in a glovebox and used as received. Diphenyl phosphate (DPP) was purchased from Sigma-Aldrich. Prior to use DPP was dried under vacuum overnight and stored in a glovebox. δ-Valerolactone (VL) was purchased from Alfa Aesar. 3-Methyl-δ-valerolactone (3MeVL) was synthesised according to literature.[1] δ-Hexalactone (HL) was purchased from Sigma Aldrich. ε-Caprolactone (eCL) was purchased from Sigma Aldrich. 4-Methyl-ε-caprolactone (4MeCL) was synthesised according to literature.[2] 2,2-Dimethyltrimethylenecarbonate (DTC) was synthesised following an adapted literature procedure (see methods section).[3] Prior to use, DTC was twice recrystallised from anhydrous diethyl ether. All monomers were stored in a glovebox.

Size Exclusion Chromatography (SEC) was carried out on a Shimadzu LC-20AD instrument, using two PSS SDV linear M columns, in series, with a $CHCl_3$ eluent. Measurements were conducted at 30 °C, with a flow rate of 1 mL/min. Samples were detected with a differential refractive index (RI) detector. Number-average molar mass ($M_{n,SEC}$) and dispersities ($Đ_M$ (M_w/M_n)) were calculated against a polystyrene calibration (molar mass range 500 – 1000000 g mol^{-1}). The polymer samples were dissolved in HPLC-grade THF, at a concentration of *ca* 10 mg/mL, and filtered through a 0.2 µm microfilter prior to analysis.

Differential Scanning Calorimetry (DSC) was performed using a TA Discovery 25 auto instrument. Experiments were performed under N_2 flow (50 mL/min) using aluminium TZERO pans. Samples (2–5 mg) were equilibrated at 40 °C, then heated, at a rate of 20 °C/min, to 200 °C and held at 200 °C, for 5 minutes. The sample was then cooled, at a rate of 20 °C/min, to –80 °C and held at –80 °C, for 5 minutes. The sample was then heated at a rate of 10 °C/min, to 200 °C and cooled, at a rate 10 °C/min, to –80 °C for 2 successive cycles. Thermal data is reported from the second heating cycle and exotherms are up (i.e., correspond to positive values).

Thermal Gravimetric Analysis – Fourier Transform Infrared Spectroscopy (TGA-FTIR) were collected on a TGA5500 System (TA Instruments), equipped with a Nicolet iS20 FTIR spectrometer (Thermo Scientific Instruments). The TGA temperature was calibrated against standards of alumel (Curie Point = 153.0 °C), nickel (Curie point = 358.2 °C), nickel-83:cobalt-17 (Curie point = 554.4 °C) and nickel-63:cobalt-37 (Curie point = 746.4 °C). The TGA mass was calibrated against standards of 100.0000 mg and 1000.0000 mg. Detailed procedures for the TGA analysis of the recycling reactions are given in the protocol section. The FTIR spectrometer was equipped with a KBr/Ge beamsplitter, fast-recovery deuterated triglycine sulfate KBr detector and solid-state diode laser. FTIR spectra were recorded, between 400–4000 cm^{-1}, with 10 scans per spectrum, at a resolution of 8 cm^{-1}.

NMR spectra were obtained using a Bruker AVIII HD nanobay NMR spectrometer. Coupling constants are given in Hertz. Polymer recycling selectivities for monomer were determined by ^1H NMR spectroscopy.

GC-MS spectra were recorded on an Agilent 7820A, equipped with a HP5-MS ultra inert column (30 m length, 0.25 mm internal diameter, 0.25 µm film thickness), a 5977B single quad mass spectrometer, a liquid injection autosampler and He carrier gas. Data was processed using MassHunter software. Samples of 5 mg/mL were prepared in dichloromethane with 1µL injected into the instrument. Samples

were loaded on to the column, in 1:100 sample:solvent ratio, and an injection port temperature of 300 °C.The column was pressurised, at 9.1 PSI, with a column flow of 1.2 mL/min and total flow of 22.12 mL/min. Following equilibration of the column oven at 40 °C for 1 minute, the temperature was increased from 40 °C to 300 °C, at a rate of 10 °C/min, and held at 300 °C, for 3 minutes. The MS source and quadrupole temperature were 230 °C and 150 °C, respectively.

Turnover Frequency (TOF): calculations were performed at 30% conversion of the polymer, using the formula:

$$\boldsymbol{TOF} = \frac{\boldsymbol{moles\ polymer\ at\ 30\%\ conversion}}{\boldsymbol{moles\ of\ catalyst}} / \boldsymbol{time\ (h)}$$

Equation S1

Error analysis all reactions were repeated at least in duplicate. For a given reaction, errors were taken as the standard deviation of the mean and a percent error was calculated as standard deviation/average of run *100. For a given polymer, the average percent error was calculated for depolymerizations conducted with that polymer. If the percent error of a given reaction was very small (i.e. smaller than 2 standard deviations away from the average percent error), the error for that run was instead calculated as the average percent error obtained for that polymer.

Hydrolysis constant and *p*K$_h$ is defined for a hydrolysis reaction of a metal ion:

$$M^{n+} + H_2O \rightleftharpoons M^{(n-1)+}OH + H^+$$

Equation S2

Where K_h is:

$$K_H = \frac{[M^{(n-1)+}OH][H^+\ ]}{[M^{n+}][H_2O]}$$

Equation S3

And pK_h is:

$$pK_h = -\log K_H$$

Equation S4

All values were taken from[4]

2. Methods

N.B. All polymer concentrations are calculated as concentration of the repeat unit.

For details on catalyst and monomer concentrations in polymer synthesis, see table S2.

2.1 Synthesis of 2,2,-Dimethyltrimethylene carbonate (DTC)

2,2-Dimethyltrimethylene carbonate (DTC) were synthesised following an adapted literature procedure.[3] 2,2-dimethyl-1,3-propane diol (12.5 g, 0.12 mol, 1 equv.) was mixed with anhydrous DCM (300 mL) under nitrogen, then 1,1'-carbonyldiimazole (24.3 g, 0.15 mol, 1.25 eq.) was added in portions over 1 hour. The reaction mixture was washed with 1 M HCl (2 x 250 mL) then dried over magnesium sulfate. The solvent was then removed *in vacuo* to isolate the product. DTC was isolated as a white powder

and recrystallised from diethyl ether to give colourless needles (7.2 g, 0.0553 mol, 46 %). See section 5 for product characterisation.

2.2 Polymerization of monomers using diphenyl phosphate catalyst

In a nitrogen-filled glovebox, delta valerolactone (1.00 g, 10 mmol, 100 equiv., $[\text{delta valerolactone}]_0$ = 9.98 M) benzene dimethanol (13.8 mg, 0.1 mmol, 1 equiv.) and diphenyl phosphate (DPP, 25.0 mg, 0.1 mmol, 1 equiv.) were added to a polymerization vial. After stirring for 3 h, the reaction mixture had solidified. The reaction was quenched with excess triethyl amine (~40 µL) and dissolved in dichloromethane. To remove the catalyst, the polymer was precipitated from dichloromethane in methanol (~30 mL), centrifuged (3900 rpm, 4 min), then the supernatant was decanted. The solid obtained was redissolved in DCM (~5 mL), then the precipitation in methanol was repeated a further two times. The product was dried under vacuum overnight, then analyzed by ^{1}H NMR, SEC, DSC, and TGA. See section 5 for product characterisation.

2.3 Polymerization of monomers using 1,5,7-Triazabicyclo[4.4.0]dec-5-ene catalyst

In a nitrogen-filled glovebox, 2,2-dimethylenecarbonate (1.30 g, 10 mmol, 100 equiv.) benzene dimethanol (13.8 mg, 0.1 mmol, 1 equiv.) and 1,5,7-triazabicyclo[4.4.0]dec-5-ene (TBD, 13.9 mg, 0.1 mmol, 1 equiv.) were added to a polymerization vial and dissolved in THF (10 mL). After stirring for 10 minutes, the reaction mixture was quenched with excess benzoic acid in THF (0.5 M, ~0.5 mL). To remove the catalyst, the polymer was precipitated in cold diethyl ether (0 °C, ~30 mL), centrifuged (3900 rpm, 4 min), then the supernatant was decanted. The solid obtained was redissolved in DCM (~5 mL), then the precipitation in diethyl ether was repeated a further two times. The product was dried under vacuum overnight, then analyzed by ^{1}H NMR, SEC, DSC, and TGA. See section 5 for product characterisation.

2.4 Kinetics of Solid-State Polymer Chemical Recycling to Monomer

Exemplary method for PVL

In a nitrogen-filled glovebox, stock solutions of PVL (1.00 M, 100 mg of PVL in 1.00 mL of THF) and $Zn(Oct)_2$ (1.00 x 10^{-2} M,10.6 mg of $Zn(Oct)_2$ in 3.00 mL of THF) were prepared. The PVL stock solution (100 µL, 0.1 mmol, 1000.0 equiv.) was added to a vial containing $Zn(Oct)_2$ (10 µL, 1.00 x 10^{-4} mmol, 1.0 equiv.). The $Zn(Oct)_2$-PVL solution was thoroughly mixed before being dropcast (*ca* 3 drops, total mass once dry 1 – 2 mg) onto Platinum TGA crucibles. The solvent was allowed to evaporate at room temperature for *ca* 1 h before the crucible was. loaded into the TGA instrument for monitored solid-state depolymerization.

The polymer samples were analyzed using the following common method:

N_2 flow of 25.0 mL min^{-1}, isotherm at 130 °C for 120 minutes

The catalyst loading, reaction temperature and length of the isotherm were varied as required.

To account for any residual solvent loss from the polymer films, which will also be detected as a mass loss by TGA, data from the first 0.5 minutes of the run was removed from the analysis. The mass at 0.5 minutes was then taken as the polymer initial mass ($mass_0$) and used to calculate the change in mass of the sample using the following formula:

$$\boldsymbol{mass\ fraction = \frac{mass_t}{mass_0}}$$

Equation S5

Where $mass_t$ = mass at time t and $mass_0$ = mass at 0.5 mins

Conversion for the reaction was calculated as:

$$Conversion = 1 - \ mass\ fraction$$

Equation S6

For Eyring analysis, isotherms were conducted at 5 different temperatures between 90 – 190 °C (polymer dependent). Each isothermal measurement was repeated in triplicate.

Based on mechanistic studies, the depolymerization was shown to proceed *via* a chain-end depolymerization mechanism. Therefore, the depolymerization rate law (variation of fraction conversion with time; s^{-1}) can be expressed as:

$$rate = k_d\ [M(OR)_n]^1$$

Equation S7

Where k_d is the depolymerization rate constant and $[Zn(OR)_2\ ]$ is the concentration of *in-situ*-formed zinc polymer alkoxide. The number of propagating chains is expected to correspond to the metal's valency. Therefore, in these instances the rate equation is expressed as:

$$rate = k_d\ \ n\ [M(Oct)_n]^1$$

Equation S8

To fit the mass *vs* time data, as expressed in equation S1, a sigmoidal function (named logistic 1 function, in Origin 2023 software package) was used:

$$y = \frac{a}{1 + e^{-k(x - x_c)}}$$

Equation S9

Where y = polymer conversion, x = time. The parameters k (growth constant), a (final conversion) and x_c (time at maximum of the fit) are determined from the fit of the data, as indicated in Fig. S1.

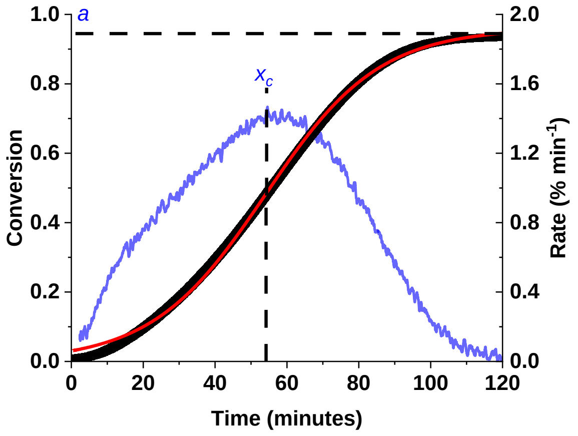

Figure S1. Example data for recycling of polyesters and polycarbonates showing conversion vs time (black), measured rate (blue), kinetic fit ($y = \frac{a}{1+e^{-k(x-x_c)}}$, red curve), a and x_c parameters. Reproduced from,[5] available under CC-BY 4.0 licence. Copyright McGuire et al 2025.

The rate of the reaction at any point of the fit can be determined by taking the derivative of the Equation S5:

$$rate = \frac{dy}{dx} = \frac{kae^{-k(x-x_c)}}{(1+e^{-k(x-x_c)})^2}$$

Equation S10

k_{obs} is taken as the maximum rate of the fit, i.e., where x = xc:

$$k_{obs} = \frac{dy}{dx}(\max of\ fit) = \frac{ka}{4}$$

Equation S11

Experiments show, k_{obs} has a linear dependence on $[Zn(Oct)_2]_0$.[5] Assuming this holds for all divalent metals

$$k_{obs} = k'_d[M(Oct)_2]_0$$

Equation S12

Assuming all polymers follow the same rate law, k_d can be approximated as:

$$k_d = \frac{k_{obs}}{n\,[M(Oct)_2]_0}$$

Equation S13

2.5 Procedure for end-capping PVL

Following an adapted literature procedure,[6] under an N_2 atmosphere, PVL (300 mg, 3 mmol, 1 equiv.) and triethylamine (0.22 mL, 3 mmol, 1 equiv.) were added to a Schlenk tube and dissolved in dichloromethane (3 mL). The solution was cooled to 0 °C and acetyl chloride (0.15 mL, 2.1 mmol, 0.7 equiv) was added dropwise. An orange/brown precipitate immediately formed. The suspension was left to stir overnight before precipitation into methanol. The sample was redissolved in dichloromethane and precipitated two more times to yield acetyl-end capped PVL (PVL-OAc) as an off white powder (221 mg, 74% yield).

3. Monomer, polymer and catalyst properties

Table S1. Catalyst properties

Metal	pK_h[a]	Ionic radius[b]	Oxophilicity[c]
Bi(III)	0.92 (±0.15)	0.96	0.2
Sn(II)	3.53 (±0.4)	1.02	0.4
Y(III)	7.77 (±0.06)	0.9	0.8
Zn(II)	8.94 (±0.06)	0.74	0.2
Co(II)	9.61 (±0.17)	0.745	0.4
Mg(II)	11.7 (±0.04)	0.72	0.6
Ca(II)	12.57 (±0.03)	1	0.3
Ba(II)	13.32 (±0.07)	1.35	0.7

[a]Taken from,[4] all values correspond to monomeric aqua metal ions $M^{z+} + H_2O \rightarrow M(OH)^{(z-1)+} + H^+$ where $K_h = [M(OH)^{(z-1)+}][H^+]/[M(OH)^{(z-1)+}]$ and $pK_h = -\log K_h$. [b]Taken from.[7, 8] [c]Taken from.[9]

Table S2. Polymerization and Depolymerization Thermodynamic Parameters for Bulk Polymerization

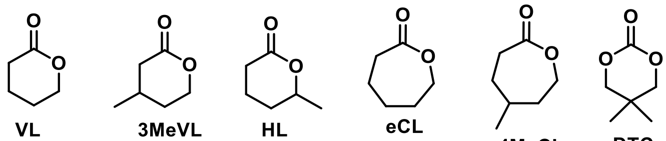

VL 3MeVL HL eCL 4MeCL DTC

Mon.	[bulk] [a] (M)	$\Delta H_p°$ (kJ mol^{-1})	$\Delta S_p°$ 1M (J mol^{-1})	ΔS_p bulk (J mol^{-1})[b]	$\Delta G_d^{130°C}$ (kJ mol^{-1})[c]	K_d 130 °C[d]	Ref
VL[e]	9.99	-27.4	-65.0	-45.9	8.9	0.070	10
3MeVL	8.76	-13.8	-46.0	-28.0	2.5	0.47	1
HL	8.76	-19.3	-62.2	-44.2	1.5	0.64	1
eCL	8.76	-13.9	-10.4	+7.6	17.0	0.043	11
4MeCL	7.79	-18.5	-23.5	-6.4	15.9	0.053	5
DTC	7.68	-25.0	-51.8	-34.8	11.0	0.038	12

All polymerization parameters are given for bulk monomer polymerization, where the monomer state is liquid (l) and the polymer state is amorphous (a), unless otherwise stated. [a] bulk concentration of polymer calculated as [moles of monomer repeat unit]/[volume of polymer] assuming density of all polymers = 1.0 g/mL. [b] Calculated according to method in [13]: ΔS_p bulk = $\Delta S_p°$ 1M + ln ([polymer bulk]$_0$). [c] $\Delta G_d^{130°C} = -\Delta G_p^{130°C}$. $\Delta G_p^{130°C} = \Delta H_p° - 403.14 \times \Delta S_p°$ bulk. [d] $K_d = exp^{\frac{-\Delta G_d}{RT}}$, where T = 130 °C. [e] Monomer state = *l*, polymer state = crystalline (c).

Table S3. Polymer Synthesis Data

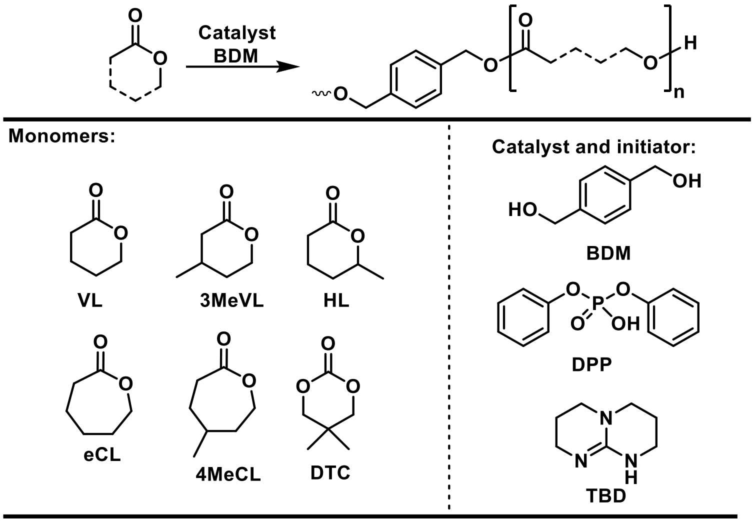

Polymer	$[cat]_0$: $[BDM]_0$: $[Mon]_0$	Cat.	Temp. (°C)	$[Mon]_0$ (M)	$M_{n,SEC}$ [a] [$Đ_M$] (g mol^{-1})	DP_{NMR} [b]	T_g [c] (°C)	T_m [d] (°C)	T_d [e] (°C)
PVL	1:1:100	DPP	25	bulk	17500 [1.09]	104	-55	53	324
P3MeVL	1:1:100	DPP	25	bulk	23100 [1.10]	125	-54	-	320
PHL	1:1:125	DPP	60	bulk	21000 [1.10]	106	-41	(54)	315
PCL	1:1:100	DPP	60	bulk	24900 [1.39]	113	-62	54	383
P4MeCL	1:1:100	DPP	25	bulk	22800 [1.39]	111	-61	-	373
PDTC	1:1:100	TBD	25	2.0 M[f]	22200 [1.20]	108	28	106	227

[a] For SEC traces see Figs. S28 – 33. Calculated by SEC relative to polystyrene standards in tetrahydrofuran (THF) eluent; $Đ_M = M_w/M_n$. [b] For NMR spectra, See Figs. S34 – 39. Determined by ^{1}H NMR spectroscopy ($CDCl_3$) by relative integration of benzyl protons of BDM (δ = 5.09 ppm) vs ester moiety (δ = 5.0 – 3.8 ppm) in isolated polymers. [c] For DSC thermograms, see Figs. S40 – 45. T_g = glass transition reported from second heating cycles in DSC. [d] T_m = melt transition reported from second heating cycles in DSC. Bracketed values indicated melt transition observed only on first heating cycle.[e] For TGA thermograms, see Figs. S46 – 51. T_d = degradation onset measured under N_2 flow (25 ml min^{-1}) under dynamic heating (10 °C min^{-1}). [f] in THF solvent.

4. Additional information

4.1 Recycling Catalyst Data

Table S4. Depolymerization Kinetic Data for PVL at 130 °C with 1:1000 $[M(Oct)_n]_0$:$[PVL]_0$ loading

Entry	Catalyst	k[a] (min^{-1})	a[a]	xc[a] (min)	$ka/4$ (x 10^{-5} s^{-1})	k_{obs} [b] (x 10^{-5} s^{-1})	TOF[c] (h^{-1})
1	$Bi(Oct)_3$	0.0113	0.25	158.00	1.15	1.0 ±0.2	34 ±8
		0.0157	0.13	68.78	0.818		
2	$Sn(Oct)_2$	0.0149	0.28	82.19	1.76	1.9 ±0.1	61 ± 12
		0.0147	0.34	91.76	2.09		
		0.0194	0.23	79.55	1.88		
3	$Y(Oct)_3$	0.0125	0.48	181.81	2.48	2.5 ±0.3	78 ±8
		0.0122	0.49	190.17	2.47		
4	$Zn(Oct)_2$	0.727	0.93	3.83	281	270 ±30	6600 ±60
		0.767	0.91	3.47	292		
		0.595	0.91	4.44	226		
5	$Co(Oct)_2$	0.217	0.87	10.86	78.6	77 ±3	2286 ± 91
		0.200	0.88	11.84	73.0		
		0.220	0.88	10.61	80.6		
6	$Mg(Oct)_2$	0.0208	0.46	148.52	3.94	3.8 ±0.2	95 ± 4
		0.0196	0.47	154.66	3.80		
		0.0187	0.46	164.49	3.55		
7	$Ca(Oct)_2$	0.0129	0.50	139.82	2.67	2.5 ±0.1	96 ±10
		0.0130	0.44	137.03	2.40		
8	$Ba(Oct)_2$	0.0110	0.25	154.28	1.16	1.3 ±0.1	50±5
		0.0117	0.30	142.15	1.45		

Reaction conditions: $[M(Oct)_x]_0$: $[PVL]_0$ = 1: 1000, $[M(Oct)_x]_0$ = 9.98 x 10^{-3} M in bulk polymer, in TGA crucibles, N_2 flow = 25 mL min^{-1}.[a] Determined from kinetic fits of conversion *vs* time profiles from 0 – 90% conversion, or from 0 – 5 h.[b] k_{obs} = pseudo first-order rate coefficient. Average taken of repeats listed, error = standard deviation from repeats.[c] TOF = moles of PVL consumed/time * moles of catalyst. Determined from 0 – 30% conversion or from conversion at 5 h.

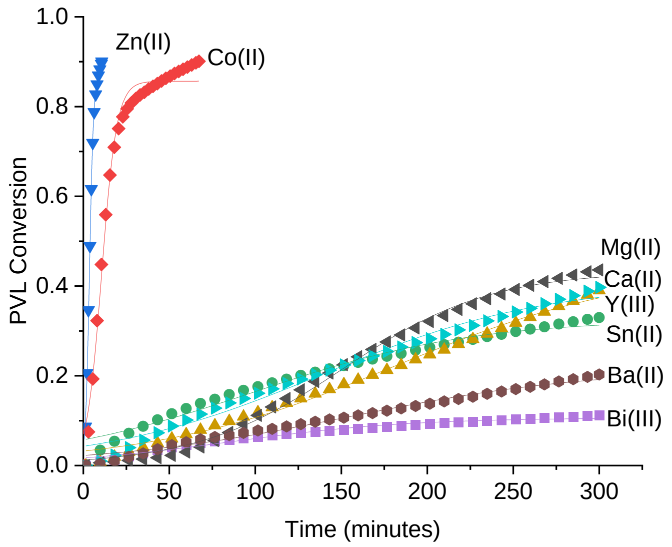

Figure S2. Plots of PVL conversion *vs* time and kinetic fits. Experimental conditions: 1: 1000 M(Oct)$_n$:PVL, 130 °C, for 5 h or until >95% conversion

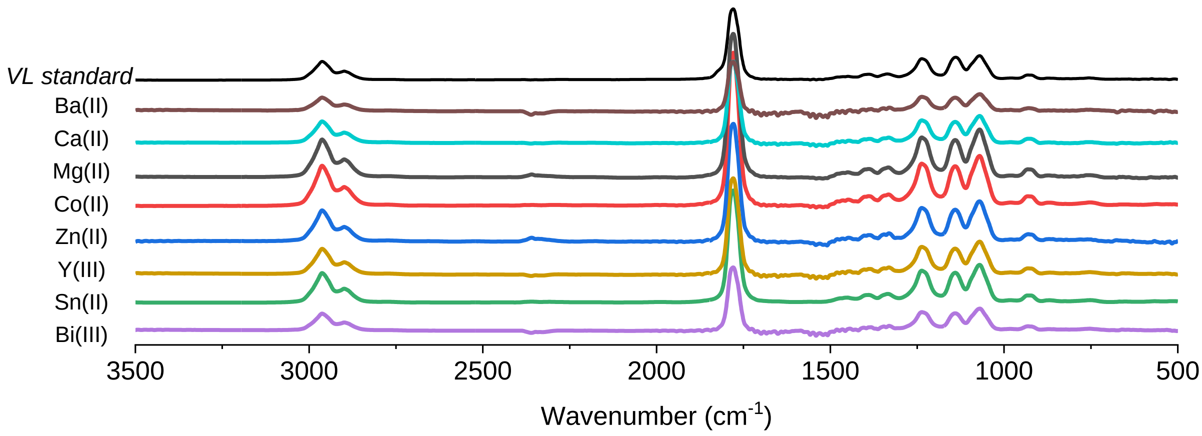

Figure S3. IR spectra showing formation of VL in TGA-IR experiments of M(Oct)$_n$:PVL mixtures. Experimental conditions: 1: 1000 M(Oct)$_n$:PVL, heating rate of 10 °C min^{-1}

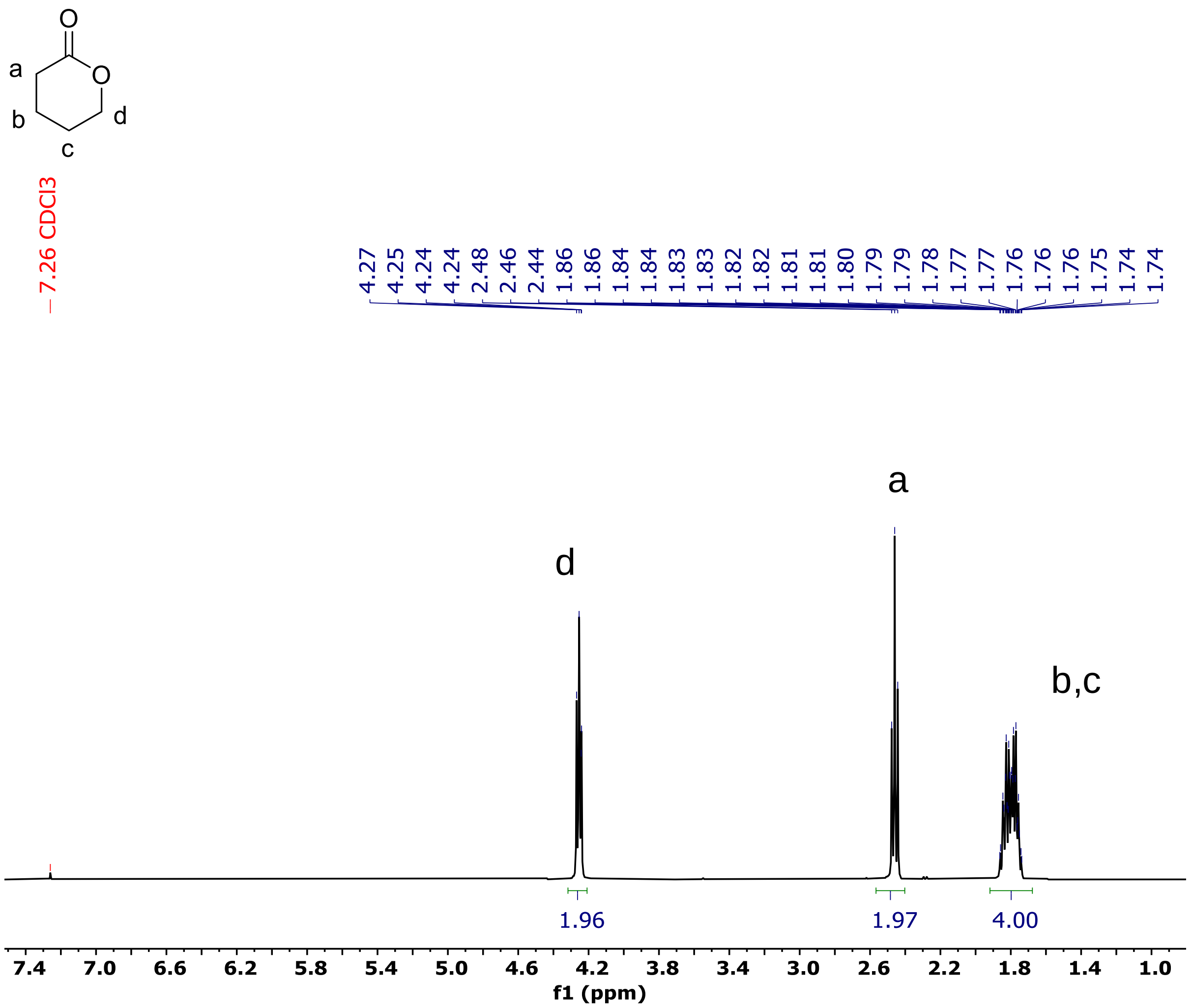

Figure S4. ^{1}H NMR spectrum (400 MHz, $CDCl_3$, 298K) of VL isolated from recycling of PVL ($[Zn(Oct)_2]_0$:$[PVL]_0$ 1:1000, 130 °C). Reproduced from,[5] available under CC-BY 4.0 licence. Copyright McGuire et al 2025.

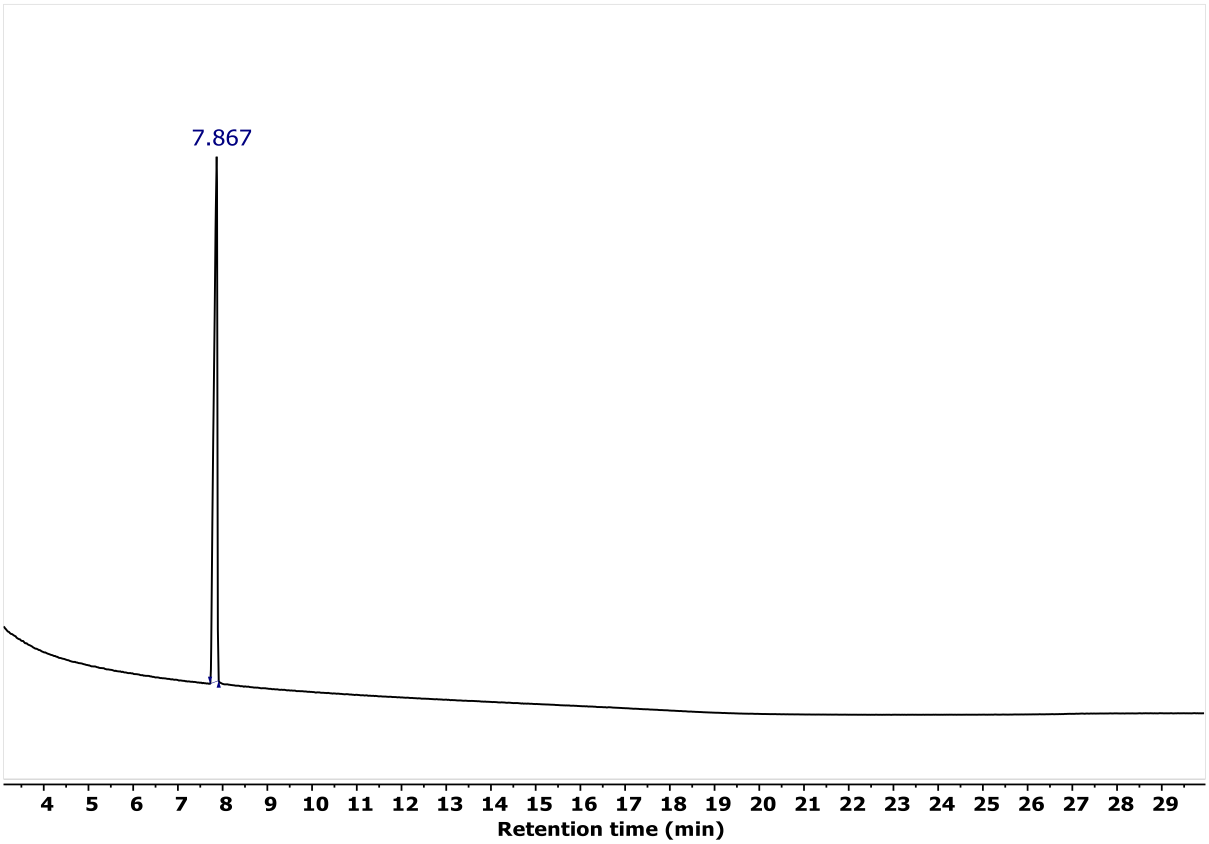

Figure S5. GC of of VL isolated from recycling of PVL ($[Zn(Oct)_2]_0$:$[PVL]_0$ 1:1000, 130 °C); pure sample of VL elutes at 7.87 minutes. Reproduced from,[5] available under CC-BY 4.0 licence. Copyright McGuire et al 2025.

Table S5. Depolymerization Kinetic Data for PCL at 160 °C with 1:100 $[M(Oct)_n]_0$: $[PCL]_0$ loading

Entry	Catalyst	k[a] (min^{-1})	a[a]	xc[a] (min)	$ka/4$ (x 10^{-5} s^{-1})	k_{obs} [b] (x 10^{-5} s^{-1})	TOF[c] (h^{-1})
1	$Bi(Oct)_3$	0.00915	0.44	239.64	1.67	1.7 ±0.2	6±1
		0.00923	0.47	249.45	1.79		
2	$Sn(Oct)_2$	0.0116	0.19	51.15	0.918	1.1 ± 0.2	4±1
		0.0126	0.25	73.65	1.33		
3	$Y(Oct)_3$	0.0092	0.38	24.25	1.46	1.5 ±0.2	5±1
		0.0137	0.26	113.71	1.48		
4	$Zn(Oct)_2$	0.246	0.92	9.73	94.5	75 ±16	200± 40
		0.137	0.93	16.99	53.2		
		0.161	0.93	15.31	62.7		
		0.241	0.90	21.66	90.3		
5	$Co(Oct)_2$	0.0599	0.93	36.48	23.2	25 ±1	85±5
		0.0673	0.93	32.47	26.1		
6	$Mg(Oct)_2$	0.0223	1.03	123.50	9.56	10 ±1	21±2
		0.0222	1.02	129.52	9.47		
7	$Ca(Oct)_2$	0.0148	0.82	135.02	5.04	5 ±1	20±2
		0.0158	0.81	129.40	5.32		
8	$Ba(Oct)_2$	0.00518	0.97	619.66	2.09	2.5 ±0.3	3±1
		0.00575	0.60	465.46	1.45		

Reaction conditions: $[M(Oct)_x]_0$: $[PCL]_0$ = 1: 100, $[M(Oct)_x]_0$ = 8.76 x 10^{-2} M in bulk polymer, in TGA crucibles, N_2 flow = 25 mL min^{-1}.[a] Determined from kinetic fits of conversion *vs* time profiles from 0 – 90% conversion, or from 0 – 5 h.[b] k_{obs} = pseudo first-order rate coefficient. Average taken of repeats listed, error = standard deviation from repeats.[c] TOF = moles of PCL consumed/time * moles of catalyst. Determined from 0 – 30% conversion or from conversion at 5 h.

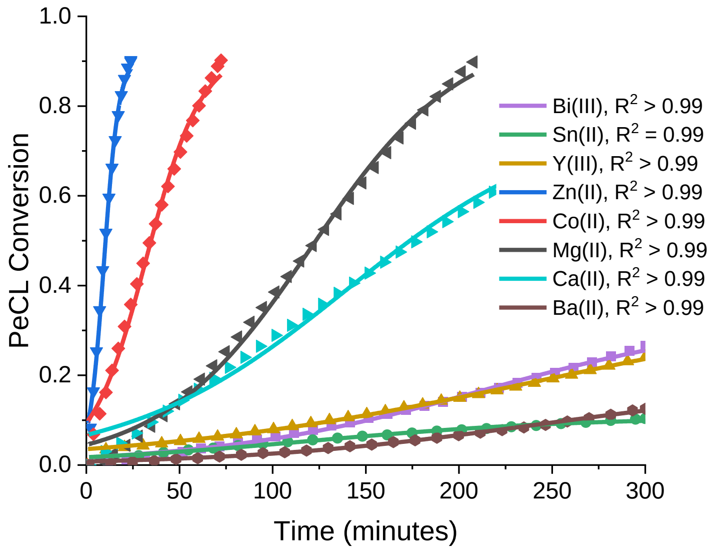

Figure S6. Plots of PCL conversion *vs* time and kinetic fits. Experimental conditions: 1: 100 M(Oct)$_n$:PCL, 160 °C, for 5 h or until >95% conversion

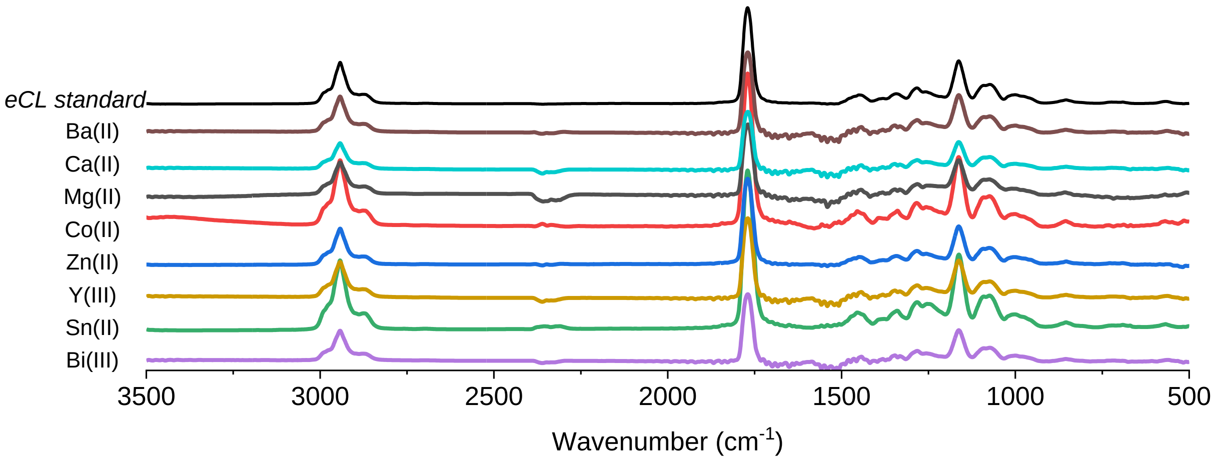

Figure S7. IR spectra showing formation of CL in TGA-IR experiments of M(Oct)$_n$:PCL mixtures. Experimental conditions: 1: 100 M(Oct)$_n$:PCL, heating rate of 10 °C min^{-1}

Table S6. Depolymerization Kinetic Data for PHL at 150 °C with 1:100 $[M(Oct)_n]_0$: $[PHL]_0$ loading

Entry	Catalyst	*k*[a] (min^{-1})	*a*[a]	*xc*[a] (min)	*ka/4* (x 10^{-5} s^{-1})	k_{obs} [b] (x 10^{-5} s^{-1})	TOF[c] (h^{-1})
1	$Bi(Oct)_3$	0.0149	0.37	102.85	2.31	2.4 ±0.2	8±1
		0.0167	0.35	114.65	2.40		
2	$Sn(Oct)_2$	0.0159	0.50	46.82	3.30	4.1 ±0.1	33±6
		0.0172	0.68	64.37	4.88		
3	$Y(Oct)_3$	0.0135	0.26	306.10	1.46	1.6 ±0.2	6±1
		0.0138	0.31	116.44	1.79		
4	$Zn(Oct)_2$	1.24	0.92	2.63	474	470 ±50	930 ±10
		1.20	0.92	2.62	457		
		1.22	0.92	2.60	465		
5	$Co(Oct)_2$	0.749	0.88	5.08	274	315 ±30	383± 40
		0.922	0.87	3.92	335		
6	$Mg(Oct)_2$	0.106	1.10	30.62	48.8	48 ±1	85±1
		0.101	1.11	31.97	46.8		
7	$Ca(Oct)_2$	0.0499	0.94	56.84	19.5	17 ±2	40±5
		0.0373	0.96	74.85	15.0		
8	$Ba(Oct)_2$	0.0141	0.37	166.02	2.17	2.7 ±0.5	8±1
		0.0155	0.50	159.22	3.23		

Reaction conditions: $[M(Oct)_x]_0$: $[PHL]_0$ = 1: 100, $[M(Oct)_x]_0$ = 8.76 x 10^{-2} M in bulk polymer, in TGA crucibles, N_2 flow = 25 mL min^{-1}.[a] Determined from kinetic fits of conversion *vs* time profiles from 0 – 90% conversion, or from 0 – 5 h.[b] k_{obs} = pseudo first-order rate coefficient. Average taken of repeats listed, error = standard deviation from repeats.[c] TOF = moles of PHL consumed/time * moles of catalyst. Determined from 0 – 30% conversion or from conversion at 5 h.

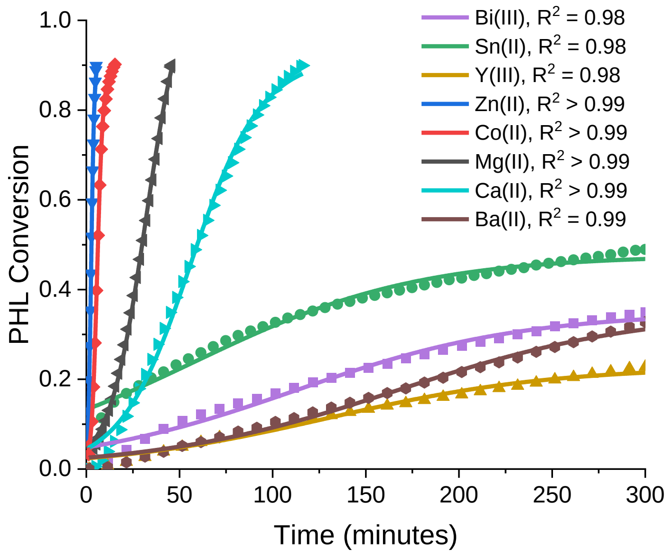

Figure S8. Plots of PHL conversion *vs* time and kinetic fits. Experimental conditions: 1: 100 $M(Oct)_n$:PHL, 150 °C, for 5 h or until >95% conversion

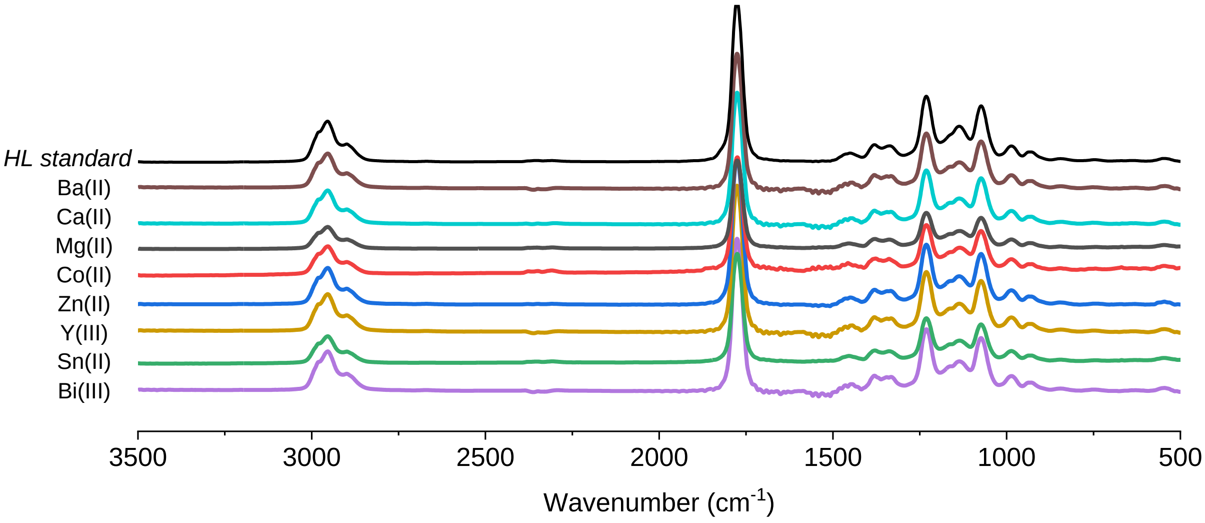

Figure S9. IR spectra showing formation of HL in TGA-IR experiments of $M(Oct)_n$:PHL mixtures (1:100). Experimental conditions: 1: 100 $M(Oct)_n$:PHL, heating rate of 10 °C min^{-1}

Table S7. Depolymerization Kinetic Data for PDTC at 150 °C with 1:1000 $[M(Oct)_n]_0$: $[PDTC]_0$ loading

Entry	Catalyst	k[a] (min^{-1})	a[a]	xc[a] (min)	$ka/4$ ($x10^{-5}$ s^{-1})	k_{obs} [b] ($x10^{-5}$ s^{-1})	TOF[c] (h^{-1})
1	$Bi(Oct)_3$	0.00875	0.18	101.31	0.647	0.71 ±0.07	32 ±3
		0.0112	0.17	76.16	0.781		
2	$Sn(Oct)_2$	0.0297	0.048	46.34	0.589	0.51 ±0.08	16 ±2
		0.0220	0.048	46.61	0.427		
3	$Y(Oct)_3$	0.00959	0.10	57.93	0.416	0.5 ±0.1	22±2
		0.0126	0.12	48.14	0.630		
4	$Zn(Oct)_2$	0.272	0.92	10.05	104	110 ± 4	2600 ±260
		0.299	0.92	9.49	114		
		0.288	0.92	9.96	110		
5	$Co(Oct)_2$	0.0809	0.91	32.55	30.8	32 ±1	853 ±50
		0.0882	0.91	29.23	33.4		
6	$Mg(Oct)_2$	0.0293	0.79	66.97	9.59	9.1 ±0.5	356 ±33
		0.0249	0.83	83.13	8.59		
7	$Ca(Oct)_2$	0.0148	0.54	135.96	3.31	3.4 ±0.1	120 ±2
		0.0150	0.55	137.49	3.46		
8	$Ba(Oct)_2$	0.0130	0.39	174.14	2.10	1.9 ±0.2	66 ±2
		0.0109	0.39	165.34	1.75		

Reaction conditions: $[M(Oct)_x]_0$: $[PDTC]_0$ = 1: 1000, $[M(Oct)_x]_0$ = 7.68 x 10^{-3} M in bulk polymer, in TGA crucibles, N_2 flow = 25 mL min^{-1}.[a] Determined from kinetic fits of conversion *vs* time profiles from 0 – 90% conversion, or from 0 – 5 h.[b] k_{obs} = pseudo first-order rate coefficient. Average taken of repeats listed, error = standard deviation from repeats.[c] TOF = moles of PDTC consumed/time * moles of catalyst. Determined from 0 – 30% conversion or from conversion at 5 h.

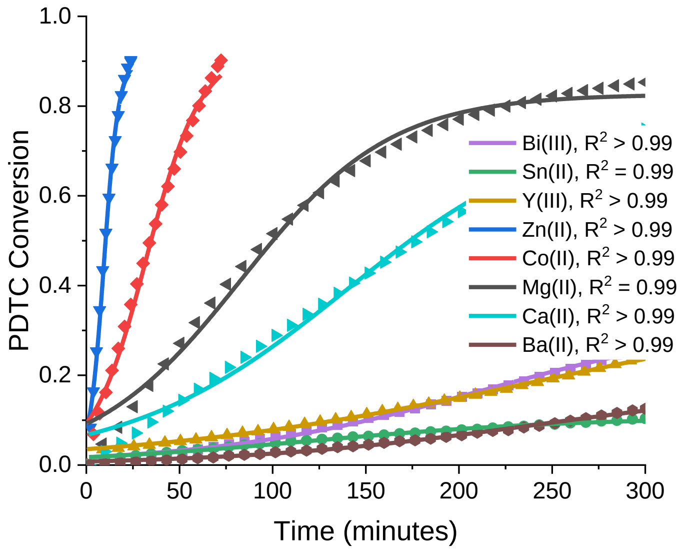

Figure S10. Plots of PDTC conversion *vs* time and kinetic fits. Experimental conditions: 1: 1000 $M(Oct)_n$:PDTC, 150 °C, for 5 h or until >95% conversion

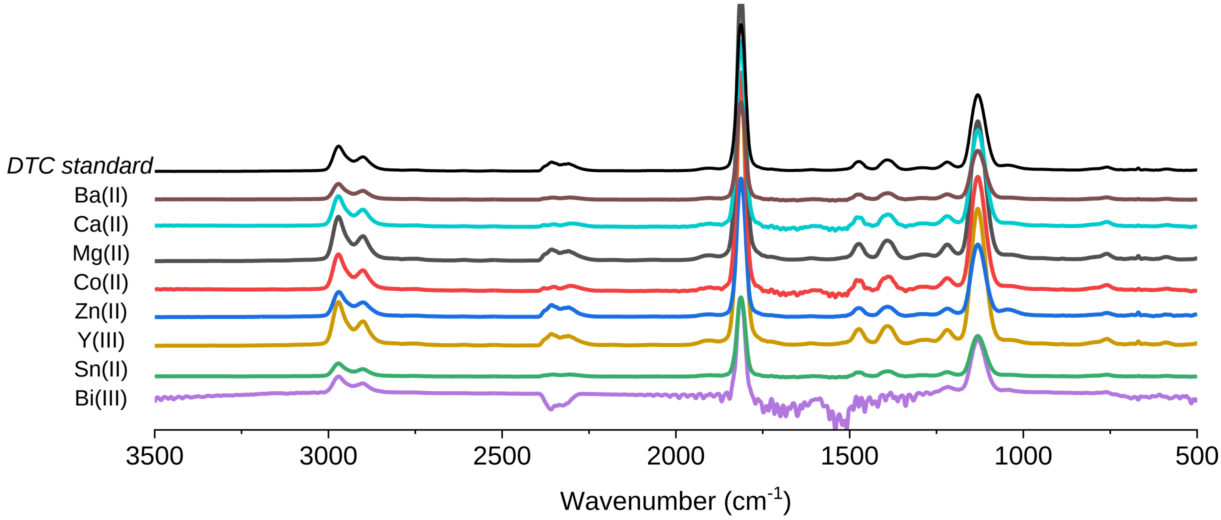

Figure S11. IR spectra showing formation of DTC in TGA-IR experiments of $M(Oct)_n$:PDTC mixtures. Experimental conditions: 1: 1000 $M(Oct)_n$:PDTC, heating rate of 10 °C min^{-1}

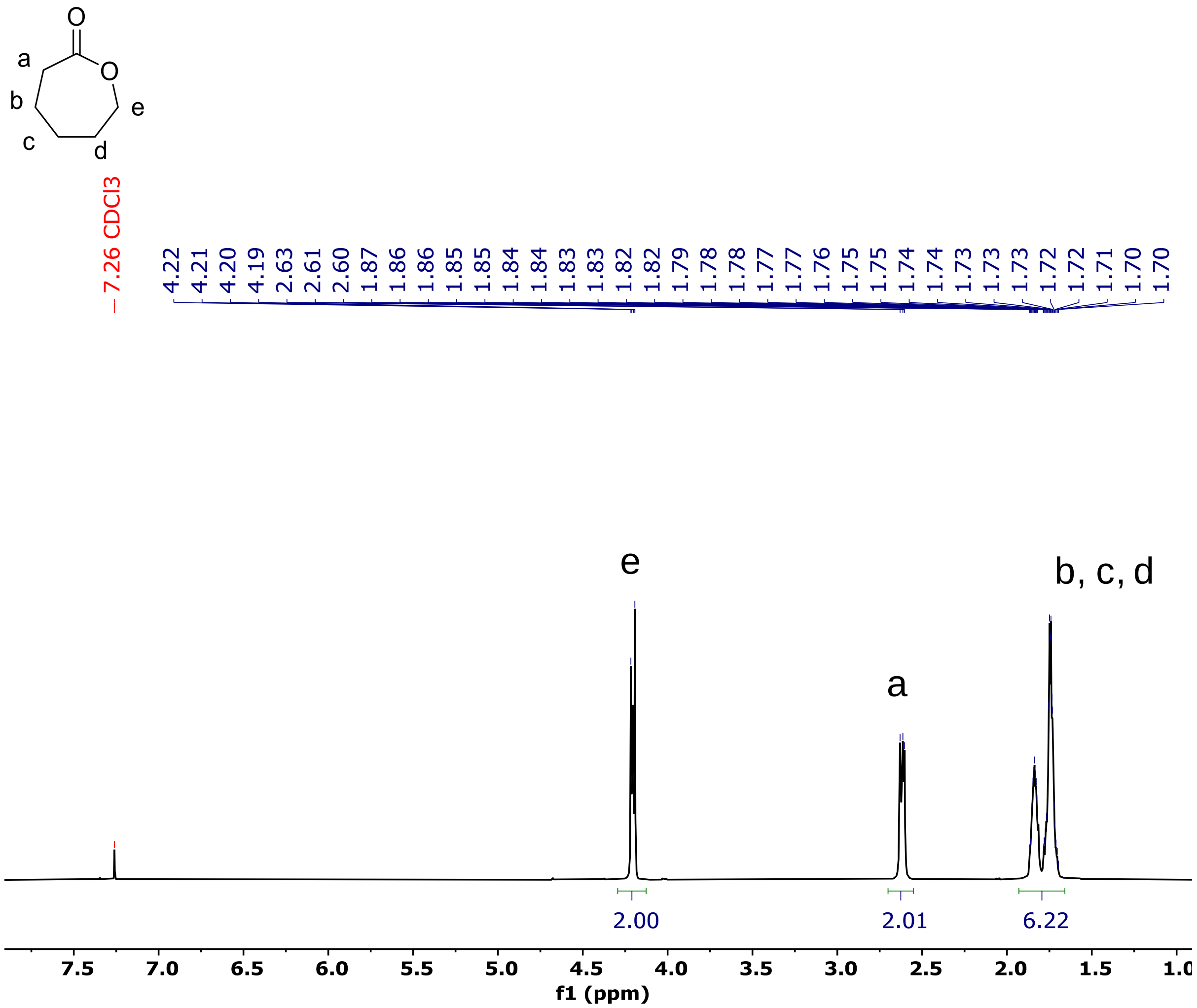

Figure S12. ^{1}H NMR spectrum (400 MHz, $CDCl_3$, 298K) of CL isolated from recycling of PCL ($[Zn(Oct)_2]_0$:$[PCL]_0$ 1:100, 160 °C). Reproduced from,[5] available under CC-BY 4.0 licence. Copyright McGuire et al 2025.

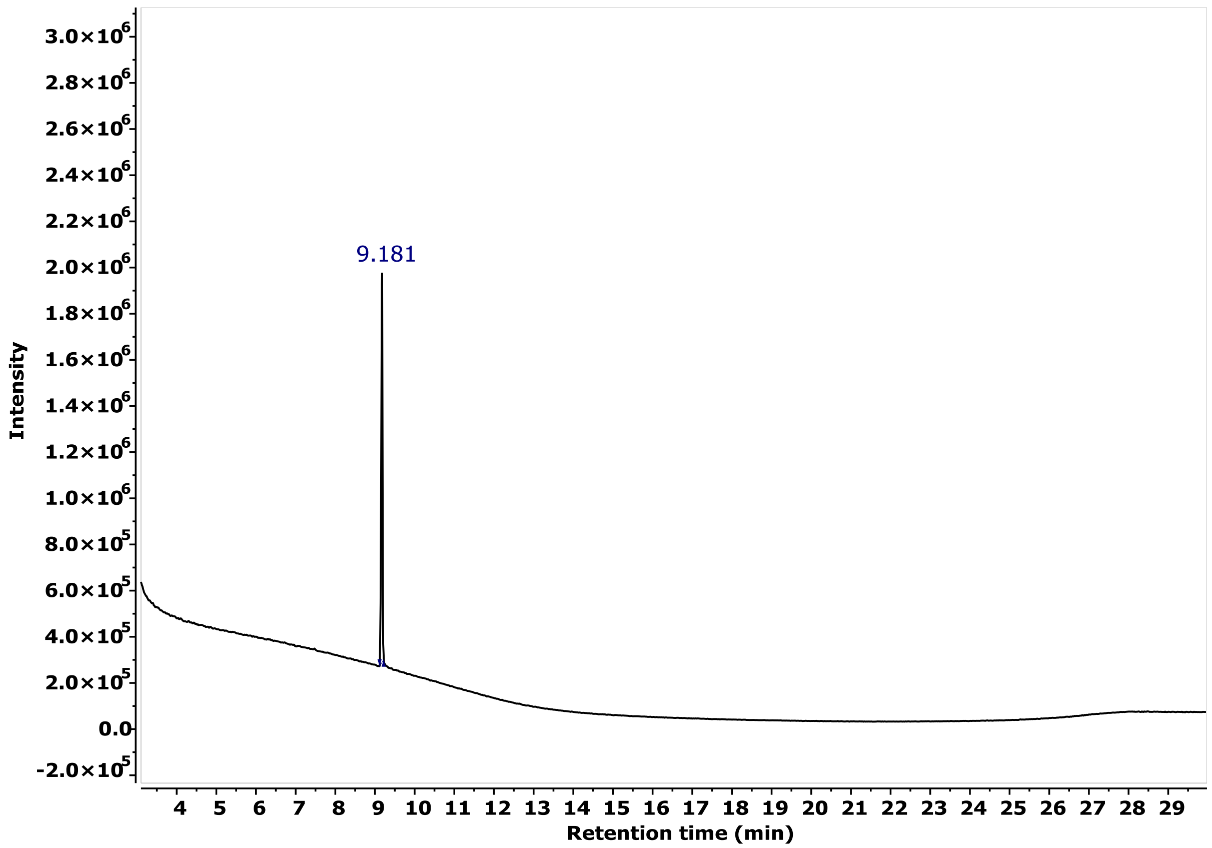

Figure S13. GC of CL isolated from recycling of PCL ($[Zn(Oct)_2]_0$:$[PCL]_0$ 1:100, 160 °C). Reproduced from,[5] available under CC-BY 4.0 licence. Copyright McGuire et al 2025.

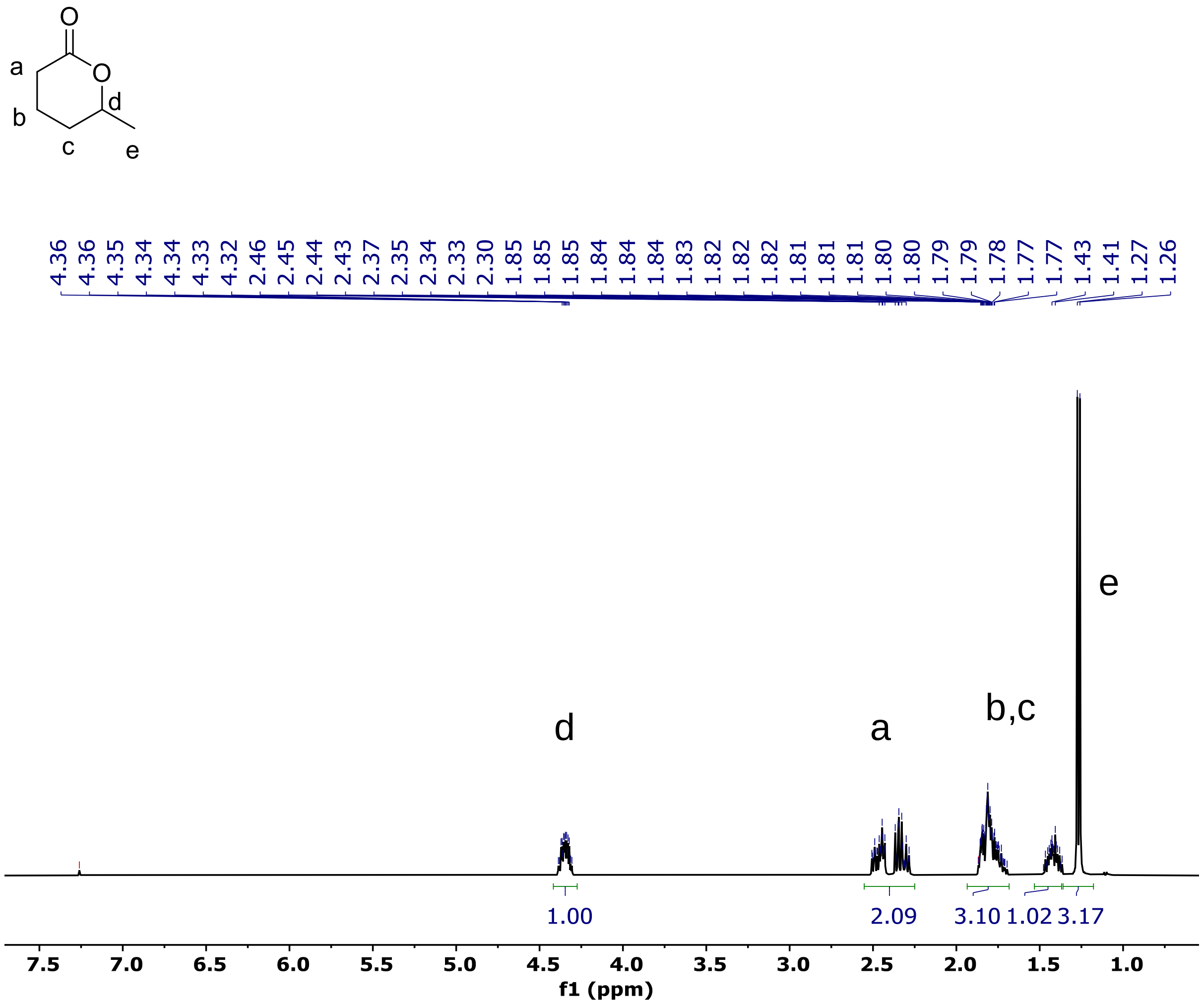

Figure S14. ^{1}**H NMR spectrum (400 MHz, $CDCl_3$, 298K) of HL isolated from recycling of PHL ([Zn(Oct)$_2$]$_0$:[PHL]$_0$ 1:100, 160 °C). Reproduced from,[5] available under CC-BY 4.0 licence. Copyright McGuire et al 2025.**

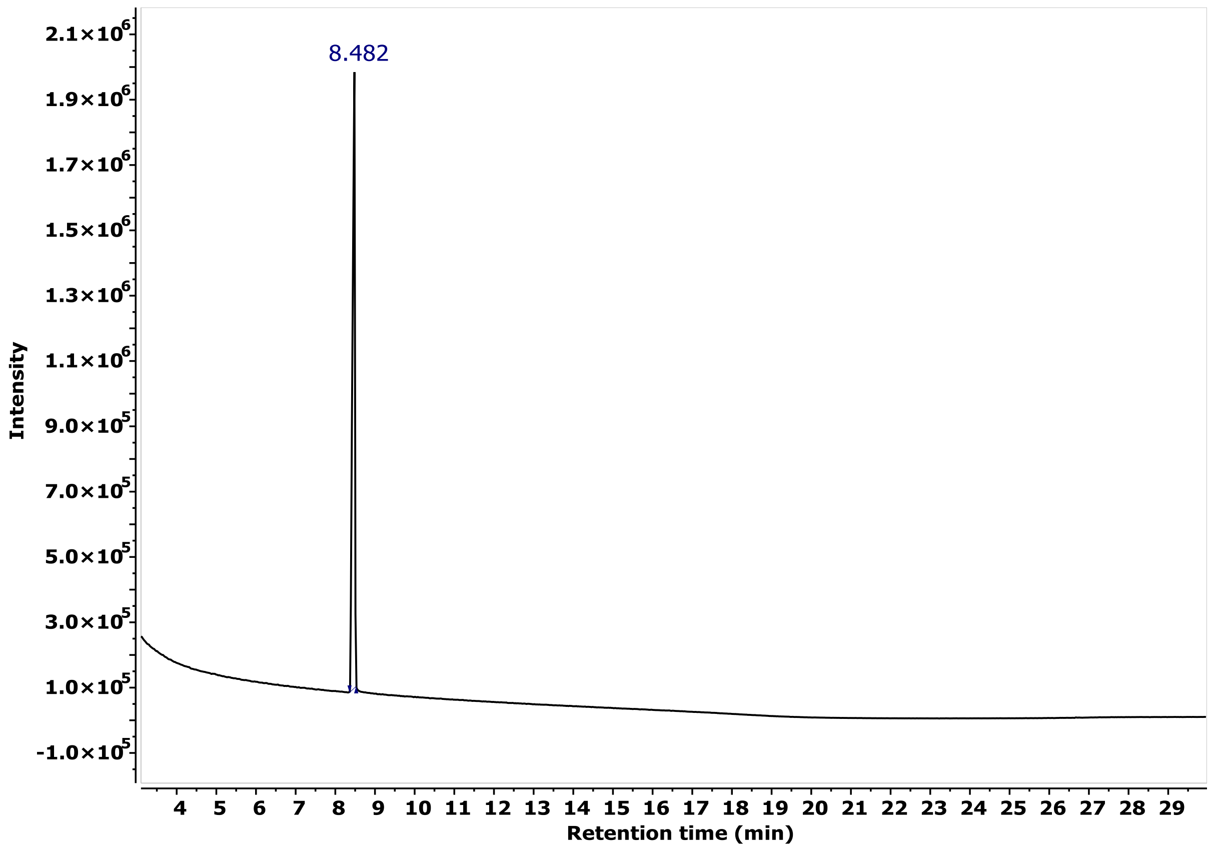

Figure S15. GC of HL isolated from recycling of of PHL ($[Zn(Oct)_2]_0$:$[PHL]_0$ 1:100, 160 °C). Pure sample of HL elutes at 8.48 minutes. Reproduced from,[5] available under CC-BY 4.0 licence. Copyright McGuire et al 2025.

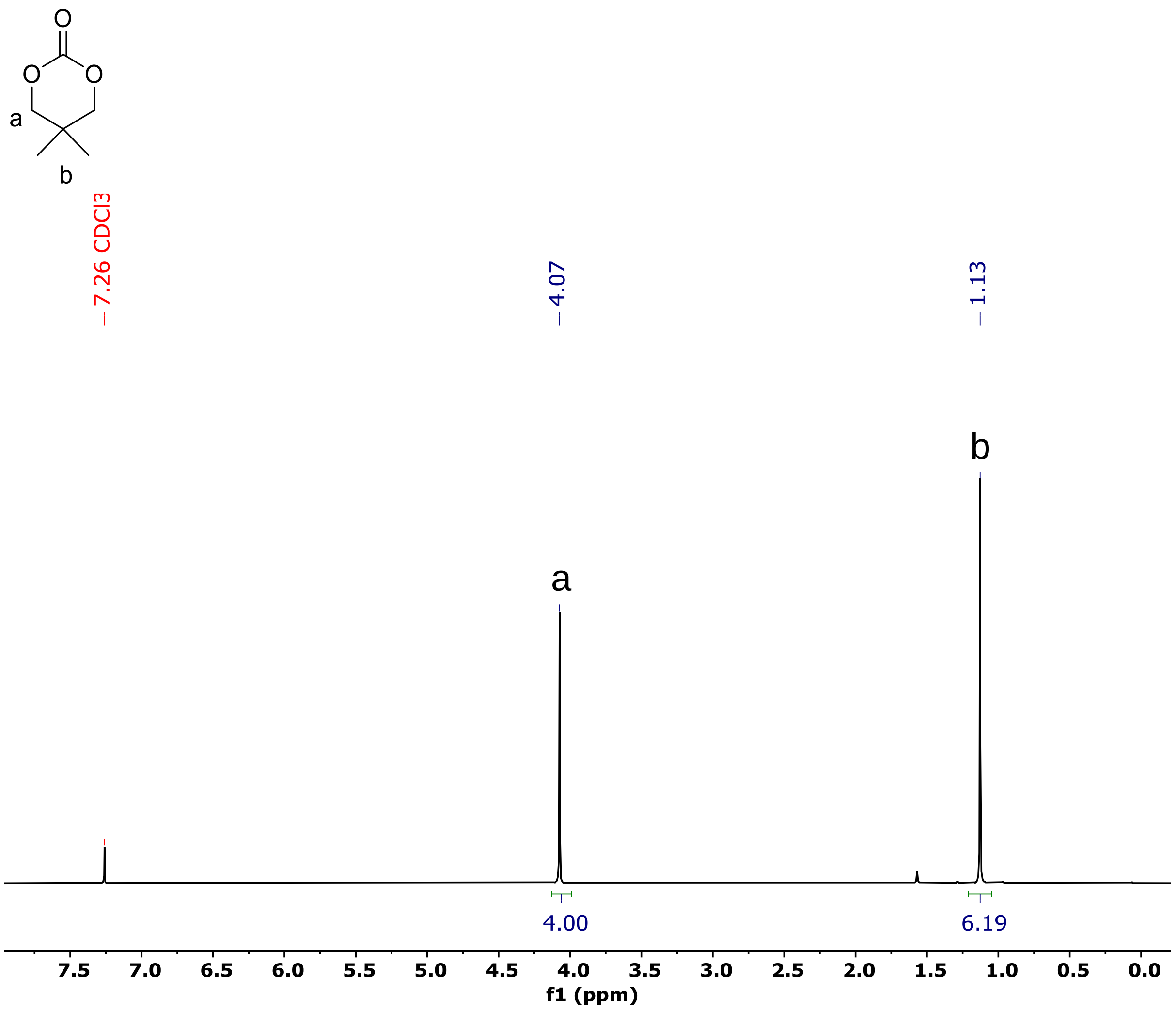

Figure S16. **^{1}H NMR spectrum (400 MHz, $CDCl_3$, 298K) of DTC isolated from recycling of PDTC ($[Zn(Oct)_2]_0$:$[PDTC]_0$ 1:100, 160 °C). Reproduced from,[5] available under CC-BY 4.0 licence. Copyright McGuire et al 2025.**

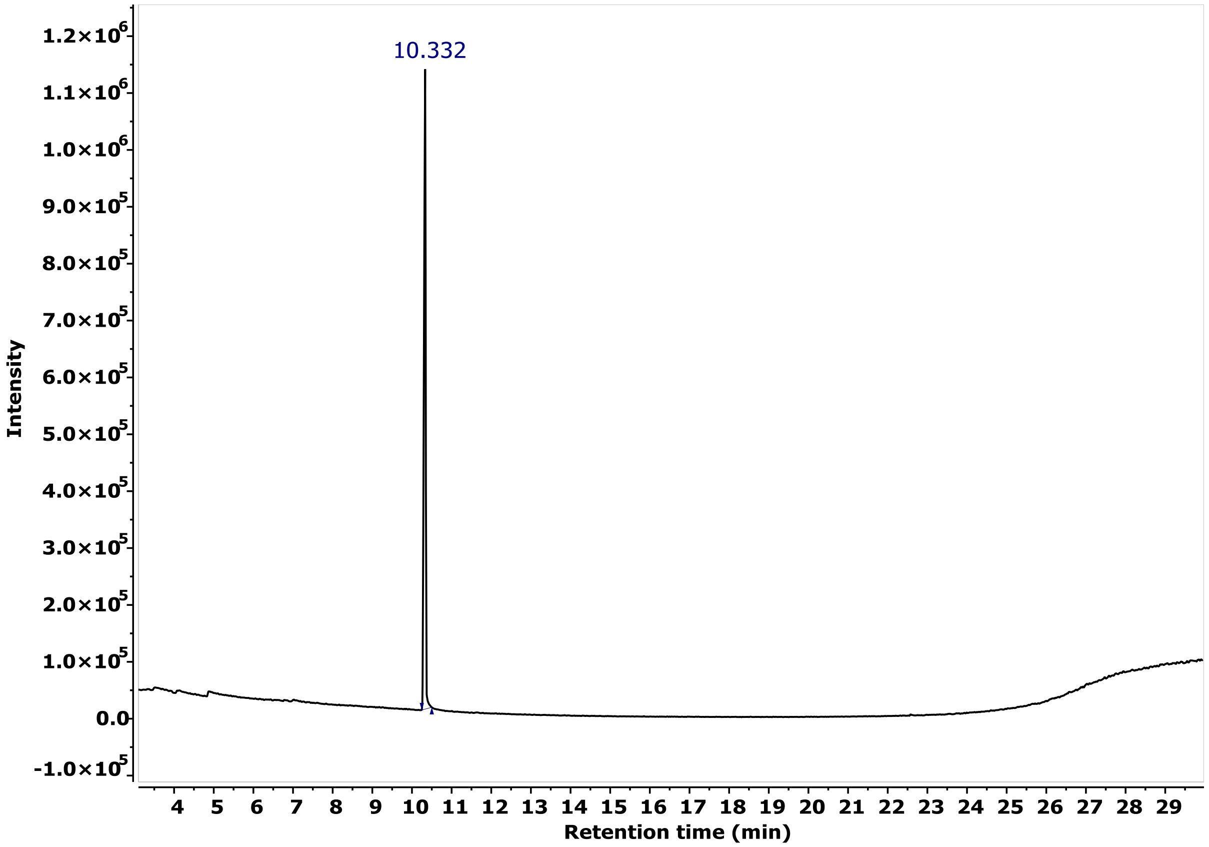

Figure S17. GC of DTC isolated from recycling of PDTC ($[Zn(Oct)_2]_0$:$[DTC]_0$ 1:100, 160 °C). Reproduced from,[5] available under CC-BY 4.0 licence. Copyright McGuire et al 2025.

4.2 Investigation of the PVL Recycling Mechanism using $Zn(Oct)_2$, $Co(Oct)_2$, $Mg(Oct)_2$ and $Sn(Oct)_2$ Catalysts at 130 °C

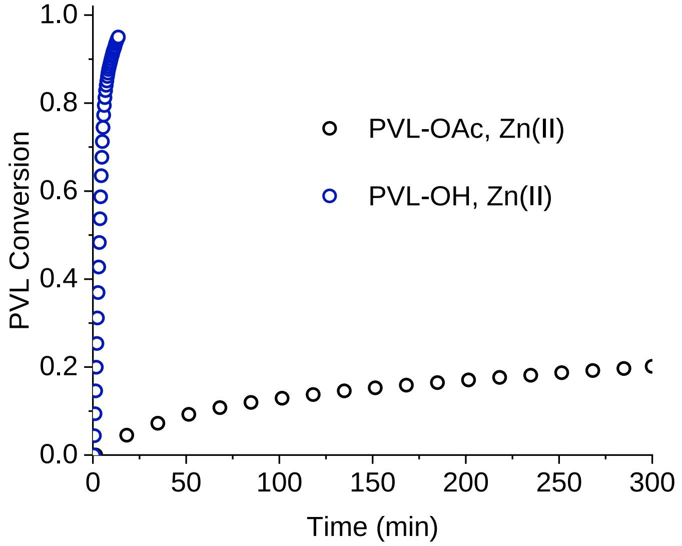

Figure S18. Plots of PVL conversion *vs* time for hydroxyl-end capped PVL (PVL-OH) and acetyl-end capped PVL (PVL-OAc) catalysed by $Zn(Oct)_2$. Experimental conditions: 1: 1000 $Zn(Oct)_2$: PVL, 130 °C, for 5 h or until >95% conversion

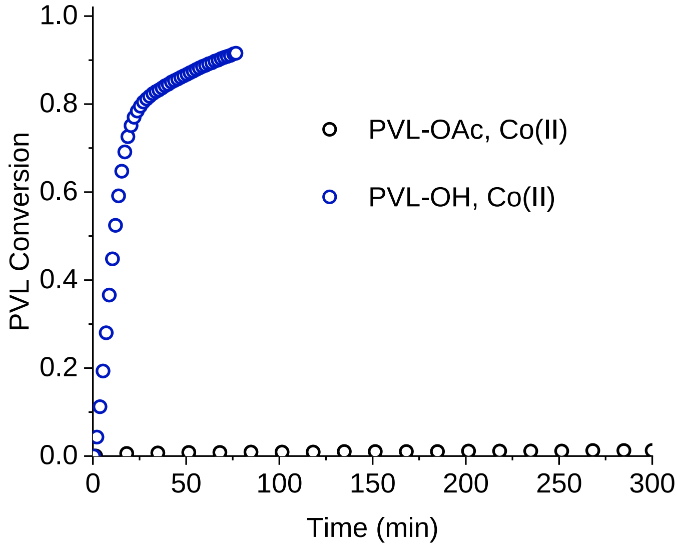

Figure S19. Plots of PVL conversion *vs* time for hydroxyl-end capped PVL (PVL-OH) and acetyl-end capped PVL (PVL-OAc) catalysed by Co(Oct)$_2$. Experimental conditions: 1: 1000 Co(Oct)$_2$: PVL, 130 °C, for 5 h or until >95% conversion

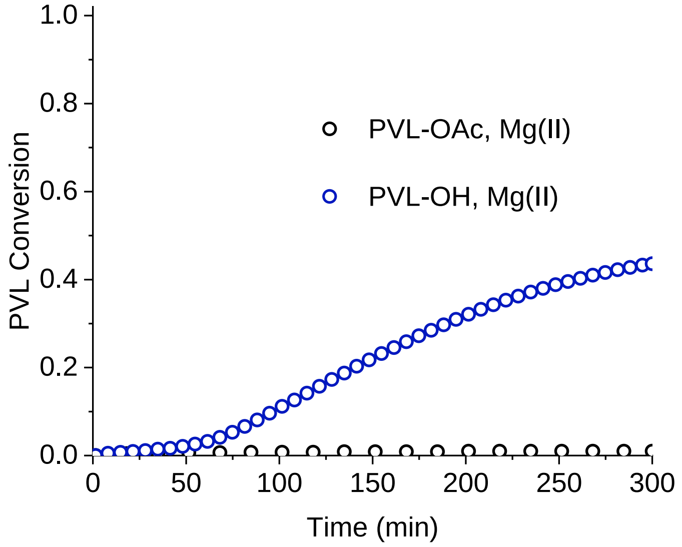

Figure S20. Plots of PVL conversion *vs* time for hydroxyl-end capped PVL (PVL-OH) and acetyl-end capped PVL (PVL-OAc) catalysed by Mg(Oct)$_2$.. Experimental conditions: 1: 1000 Mg(Oct)$_2$: PVL, 150 °C, for 5 h or until >95% conversion

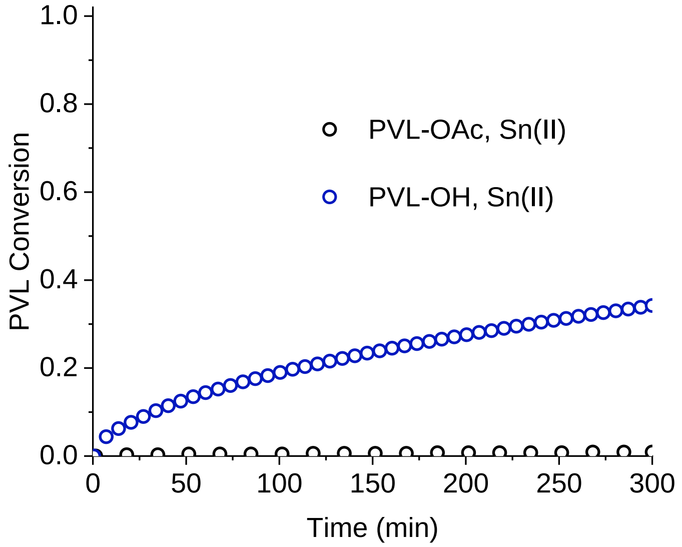

Figure S21. Plots of PVL conversion *vs* time for hydroxyl-end capped PVL (PVL-OH) and acetyl-end capped PVL (PVL-OAc) catalysed by Sn(Oct)$_2$. Experimental conditions: 1: 1000 Sn(Oct)$_2$: PVL, 150 °C, for 5 h or until >95% conversion

Table S8. Depolymerization Kinetic Data for P3MeVL, PVL, PDTC, P4MeCl and PCL at 130 °C with 1:1000 [Co(Oct)$_2$]$_0$: [polymer repeat unit]$_0$ loading

Entry	Polymer	k[a] (min^{-1})	a[a]	xc[a] (min)	$ka/4$ (x 10^{-5} s^{-1})	k_{obs} [b] (x 10^{-5} s^{-1})	k_d [c] (x 10^{-4} mol^{-1} dm^3 s^{-1})	TOF[d] (h^{-1})
1	P3MeVL[e]	0.634	0.95	6.38	252	260 ± 15	1500 ± 100	3500± 200
		0.616	0.93	6.67	240			
		0.712	0.93	5.83	277			
2	PVL[f]	0.217	0.88	10.86	78.6	77 ± 3	390 ± 20	2300±90
		0.200	0.88	11.83	73.0			
		0.220	0.88	10.60	80.6			
3	PDTC[g]	0.0233	0.91	94.04	8.91	8.0 ±0.9	52 ±5	270 ±30
		0.0195	0.88	117.09	7.13			
4	P4MeCl[h]	0.0221	0.48	88.76	4.44	4.4 ±0.3	28 ± 2	160 ±10
		0.0198	0.48	96.35	4.00			
		0.0267	0.43	74.98	4.77			
5	PCL[e]	0.00713	0.42	247.16	1.24	1.3 ± 0.1	7.4 ± 0.5	47±4
		0.00753	0.45	239.53	1.42			
		0.00752	0.39	229.35	1.22			

Reaction conditions: [Co(Oct)$_2$]$_0$: [polymer repeat unit]$_0$ = 1: 1000 in bulk polymer (see footnotes for [Co(Oct)$_2$]$_0$), in TGA crucibles, N_2 flow = 25 mL min^{-1}. [a] Determined from kinetic fits of conversion *vs* time profiles from 0 – 90% conversion, or from 0 – 5 h.[b] k_{obs} = pseudo first-order rate coefficient. Average taken of repeats listed, error = standard deviation from repeats.[c] Recycling rate coefficient, $k_d = k_{obs}/(2 \times [cat]_0)$, assuming 2 active chains per metal centre. [d] TOF = moles of polymer consumed/time * moles of catalyst. Determined from 0 – 30% conversion or from conversion at 5 h. [e] [Co(Oct)$_2$]$_0$ = 8.76 x 10^{-3} M. [f] [Co(Oct)$_2$]$_0$ = 9.98 x 10^{-3} M. [g] [Co(Oct)$_2$]$_0$ = 7.68 x 10^{-3} M.[h] [Co(Oct)$_2$]$_0$ = 7.80 x 10^{-3} M.

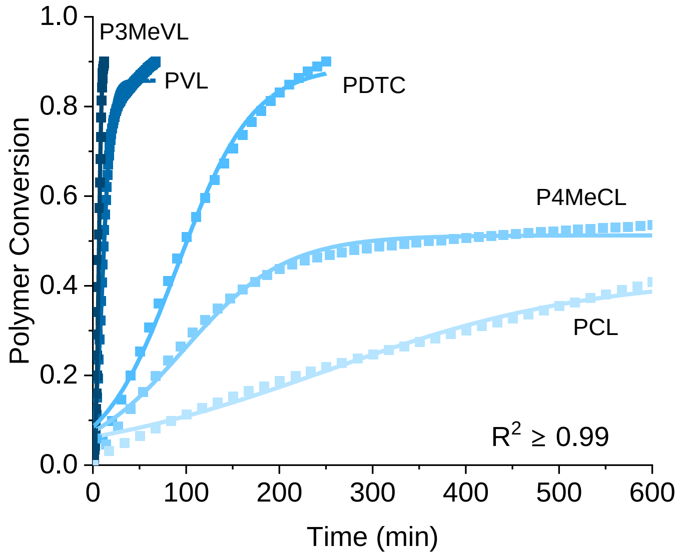

Figure S22. Plots of polymer conversion *vs* time for P3MeVL, PVL, PDTC, P4MeCL and PCL depolymerization catalysed by Co(Oct)$_2$. Experimental conditions: 1: 1000 Co(Oct)$_2$:PVL, 130 °C, for 10 h or until >95% conversion

Table S9. Depolymerization Kinetic Data for P3MeVL, PVL, PDTC, P4MeCL and PCL at 130 °C with 1:1000 $[Mg(Oct)_2]_0$: [polymer repeat unit]$_0$ loading

Entry	Polymer	k[a] (min^{-1})	a[a]	xc[a] (min)	$ka/4$ ($x 10^{-5} s^{-1}$)	k_{obs} [b] ($x 10^{-5} s^{-1}$)	k_d [c] ($x 10^{-5} mol^{-1} dm^3 s^{-1}$)	TOF[d] (h^{-1})
1	P3MeVL [e]	0.0335	0.94	59.80	13.1	11 ±2	620 ± 120	500 ± 60
		0.0288	0.94	63.60	11.2			
		0.0207	0.94	88.60	8.15			
2	PVL[f]	0.0208	0.46	148.52	3.95	3.8± 0.2	190 ± 8	95 ± 4
		0.0196	0.47	154.66	3.80			
		0.0187	0.46	164.49	3.55			
3	PDTC[g]	0.0169	0.21	117.11	1.47	1.6 ± 0.2	10 ± 1	44±4
		0.0182	0.24	104.25	1.84			
		0.0177	0.21	112.32	1.53			
4	P4MeCL [h]	0.0102	0.036	151.59	0.155	0.13 ± 0.02	8 ± 2	6±1
		0.0104	0.023	148.82	0.0987			
		0.00888	0.038	107.18	0.139			
5	PCL[e]	0.0194	0.012	116.74	0.0995	0.097 ± 0.002	5.6 ± 0.1	3±1
		0.0153	0.015	140.09	0.0956			
		0.0144	0.016	132.87	0.0972			

Reaction conditions: $[Mg(Oct)_2]_0$: [polymer repeat unit]$_0$ = 1: 1000 in bulk polymer (see footnotes for $[Mg(Oct)_2]_0$), in TGA crucibles, N_2 flow = 25 mL min^{-1}.[a] Determined from kinetic fits of conversion *vs* time profiles from 0 – 90% conversion, or from 0 – 5 h.[b] k_{obs} = pseudo first-order rate coefficient. Average taken of repeats listed, error = standard deviation from repeats.[c] Recycling rate coefficient, $k_d = k_{obs}/(2 \times [cat]_0)$, assuming 2 active chains per metal centre.[d] TOF = moles of polymer consumed/time x moles of catalyst. Determined from 0 – 30% conversion or from conversion at 5 h. [e] $[Mg(Oct)_2]_0 = 8.76 \times 10^{-3}$ M. [f] $[Mg(Oct)_2]_0 = 9.98 \times 10^{-3}$ M. [g] $[Mg(Oct)_2]_0 = 7.68 \times 10^{-3}$ M.[h] $[Mg(Oct)_2]_0 = 7.80 \times 10^{-3}$ M.

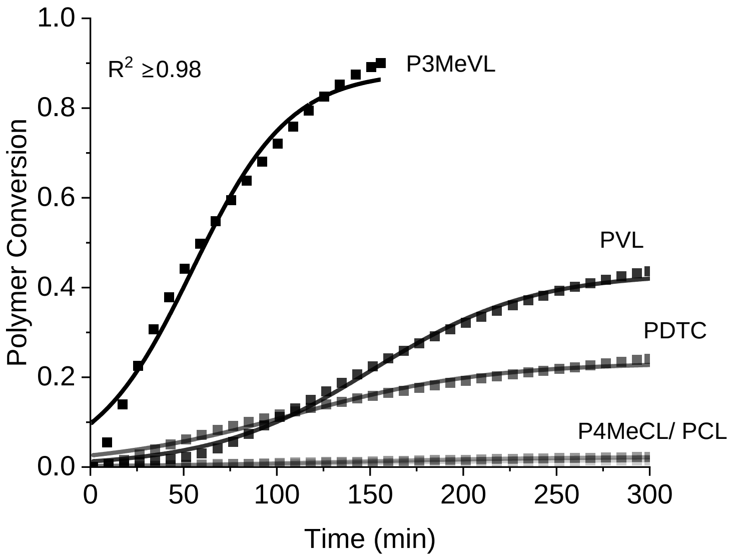

Figure S23. Plots of polymer conversion *vs* time for P3MeVL, PVL, PDTC, P4MeCL and PCL depolymerization catalysed by $Mg(Oct)_2$. Experimental conditions: 1: 1000 $Mg(Oct)_2$: PVL, 130 °C, for 5 h or until >95% conversion

Table S10. Depolymerization Kinetic Data for P3MeVL, PVL, PDTC, P4MeCl and PCL at 130 °C with 1:1000 $[Sn(Oct)_2]_0$: [polymer repeat unit]$_0$ loading

Entry	Polymer	k[a] (min^{-1})	a[a]	xc[a] (min)	$ka/4$ ($\times 10^{-5}$ s^{-1})	k_{obs} [b] ($\times 10^{-5}$ s^{-1})	k_d [c] ($\times 10^{-5}$ mol^{-1} $dm^3 s^{-1}$)	TOF[d] (h^{-1})
1	P3MeVL[e]	0.0237	0.58	43.80	5.69	6.3 ±0.5	360 ±27	470 ± 13
		0.0268	0.58	45.75	6.50			
		0.0275	0.60	44.53	6.82			
2	PVL[f]	0.0149	0.28	82.19	1.76	1.9 ±0.2	190 ± 8	61 ± 12
		0.0147	0.34	91.76	2.09			
		0.0194	0.23	79.55	1.88			
3	PDTC[g]	0.0159	0.055	85.25	0.361	2 ± 1	14 ± 7	7 ± 3
		0.0101	0.029	88.34	0.121			
		0.0112	0.034	84.36	0.158			
4	P4MeCl[h]	0.0151	0.037	132.77	0.233	0.18 ± 0.06	12 ± 4	6 ±1
		0.0149	0.035	120.31	0.214			
		0.0128	0.017	124.02	0.0929			
5	PCL[e]	0.0148	0.00	7.56	0.0289	0.036 ±0.007	2.0 ±0.4	1±1
		0.0215	0.00	6.96	0.0421			

Reaction conditions: $[Sn(Oct)_2]_0$: [polymer repeat unit]$_0$ = 1: 1000 in bulk polymer (see footnotes for $[Sn(Oct)_2]_0$), in TGA crucibles, N_2 flow = 25 mL min^{-1}.[a] Determined from kinetic fits of conversion *vs* time profiles from 0 – 90% conversion, or from 0 – 5 h.[b] k_{obs} = pseudo first-order rate coefficient. Average taken of repeats listed, error = standard deviation from repeats.[c] Recycling rate coefficient, $k_d = k_{obs}/(2 \times [cat]_0)$, assuming 2 active chains per metal centre. [d] TOF = moles of polymer consumed/time x moles of catalyst. Determined from 0 – 30% conversion or from conversion at 5 h. [e] $[Sn(Oct)_2]_0 = 8.76 \times 10^{-3}$ M. [f] $[Sn(Oct)_2]_0 = 9.98 \times 10^{-3}$ M. [g] $[Sn(Oct)_2]_0 = 7.68 \times 10^{-3}$ M.[h] $[Sn(Oct)_2]_0 = 7.80 \times 10^{-3}$ M.

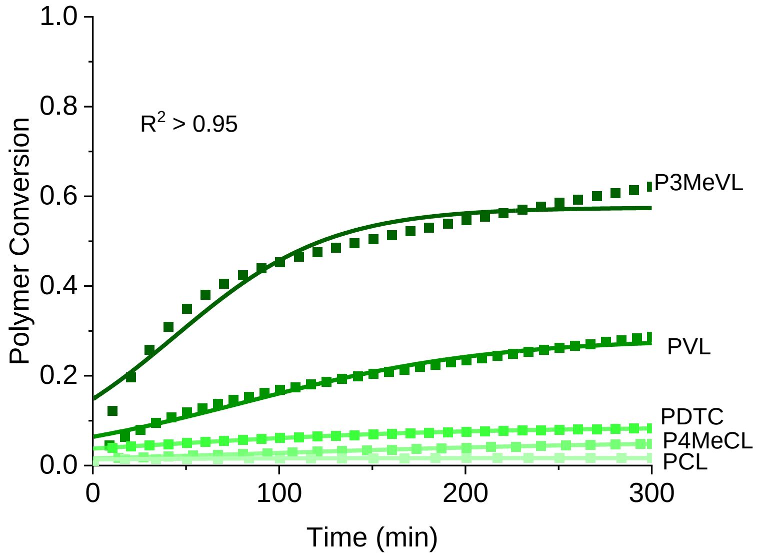

Figure S24. Plots of polymer conversion *vs* time for P3MeVL, PVL, PDTC, P4MeCL and PCL depolymerization catalysed by $Sn(Oct)_2$. Experimental conditions: 1: 1000 $Sn(Oct)_2$: PVL, 130 °C, for 5 h or until >95% conversion

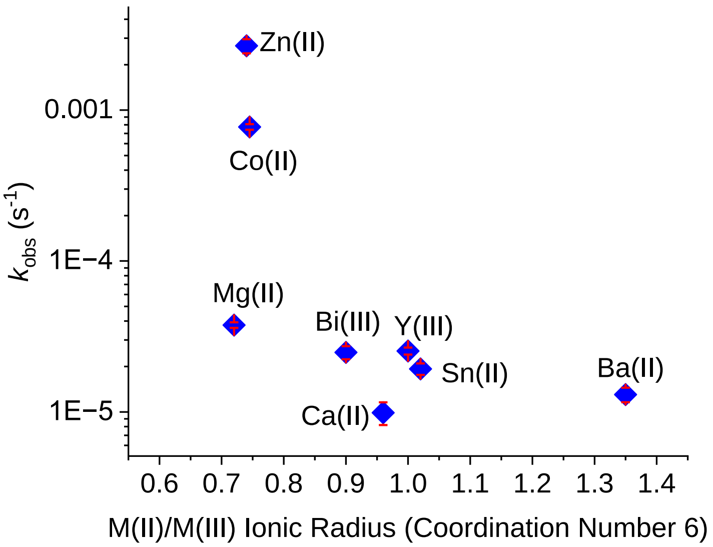

Figure S25. Plot showing k_{obs} vs metal ionic radius (coordination number 6) for the depolymerization of PVL catalysed by $M(Oct)_n$. Experimental conditions: $M(Oct)_n$: PVL, 1:1000, 130 °C for 5 h or until >95% conversion. Ionic radii were taken from[7, 8]

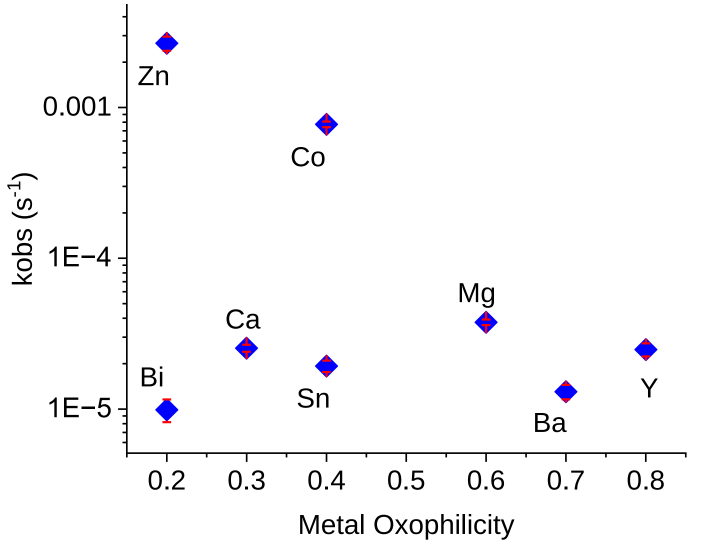

Figure S26. Plot showing k_{obs} vs metal oxophilicity for the depolymerization of PVL catalysed by $M(Oct)_n$. Experimental conditions: $M(Oct)_n$: PVL, 1:1000, 130 °C for 5 h or until >95% conversion. Oxophilicity values taken from[9]

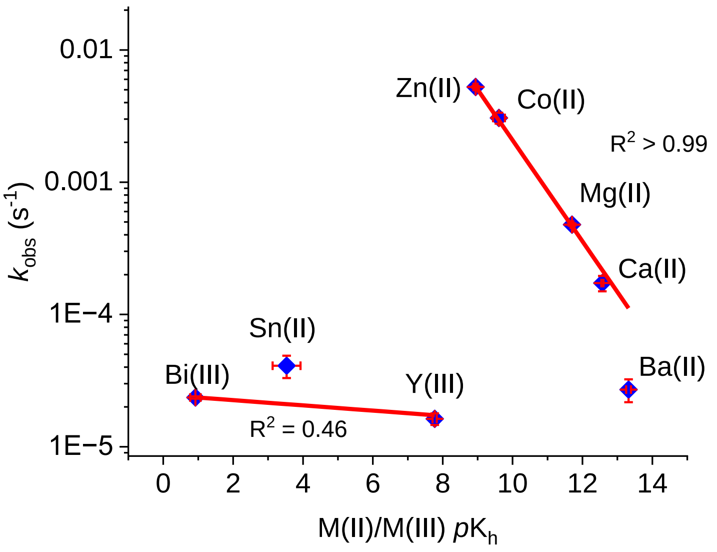

Figure S27. Plot showing k_{obs} vs metal ion pK_h for the depolymerization of PHL catalysed by $M(Oct)_n$. Experimental conditions: $M(Oct)_n$: PHL, 1:100, 150 °C for 5 h or until >95% conversion. pK_h values taken from[4]

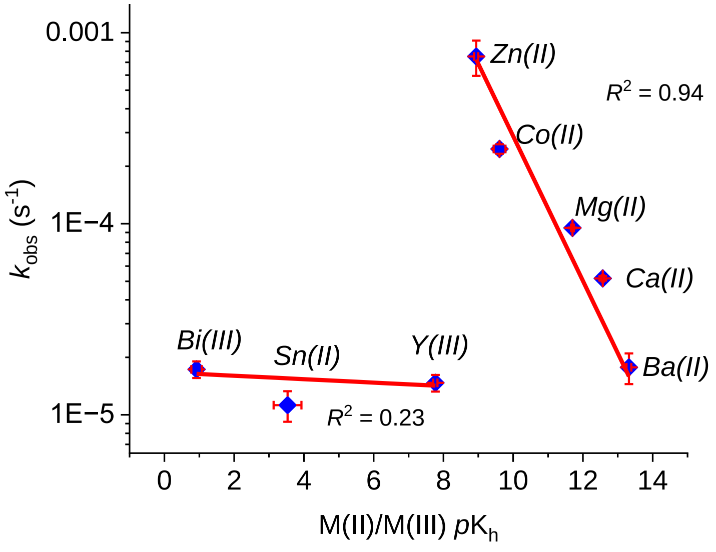

Figure S28. Plot showing k_{obs} vs metal ion pK_h for the depolymerization of PCL catalysed by $M(Oct)_n$. Experimental conditions: $M(Oct)_n$: PCL, 1:100, 160 °C for 5 h or until >95% conversion. pK_h values taken from[4]

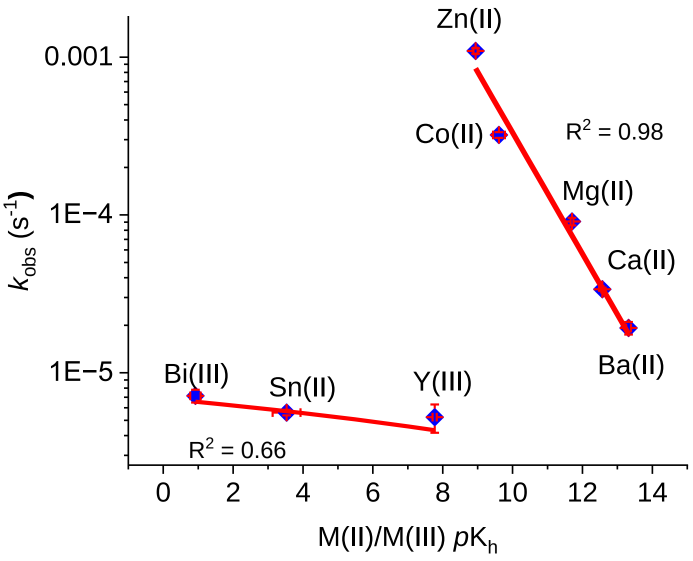

Figure S29. Plot showing k_{obs} vs metal ion pK_h for the depolymerization of PDTC catalysed by $M(Oct)_n$. Experimental conditions: $M(Oct)_n$: PDTC, 1:100, 150 °C for 5 h or until >95% conversion. pK_h values taken from[4]

4.3 Eyring analysis

Table S11. Depolymerization Kinetic Data for PVL at 100 – 140 °C with 1:1000 $[Co(Oct)_2]_0$: $[PVL]_0$ loading

Entry	Temp. (°C)	k[a] (min^{-1})	a[a]	xc[a] (min)	$ka/4$ (x 10^{-4} s^{-1})	k_{obs} [b] (x 10^{-4} s^{-1})	k_d [c] (x 10^{-3} mol^{-1} $dm^3 s^{-1}$)	TOF[d] (h^{-1})
1	100	0.033	0.814	60.7	1.12	1.1 ± 0.13	5.3 ± 0.6	410 ±70
		0.035	0.797	54.0	1.18			
		0.028	0.761	77.672	0.882			
2	110	0.055	0.832	36.919	1.91	2.0 ± 0.1	10 ± 1	720 ±12
		0.059	0.851	35.919	2.08			
		0.056	0.851	36.450	1.99			
3	120	0.073	0.840	27.600	2.55	3.3 ± 0.7	16 ± 4	1100± 150
		0.088	0.841	23.721	3.08			
		0.119	0.854	14.469	4.24			
4	130	0.217	0.867	10.861	7.86	7.7 ± 0.3	390 ± 20	2300±90
		0.200	0.876	11.835	7.30			
		0.220	0.881	10.609	8.06			
5	140	0.388	0.833	6.878	13.5	12 ± 1	610 ± 49	3900± 300
		0.298	0.892	6.380	11.1			
		0.334	0.879	7.224	12.2			

Reaction conducted by solvent casting $Co(Oct)_2$:PVL solutions ($[Co(Oct)_2]_0$: $[PVL]_0$ = 1: 1000, $[Co(Oct)_2]_0$ = 9.98 x 10^{-3} M in bulk polymer) in TGA crucibles. N_2 flow = 25 mL min^{-1}.[a] Determined from logistic fitting of conversion *vs* time profiles from 0 – 90% mass loss or from 0 – 5 h.[b] k_{obs} = average of repeats listed, error = standard deviation from repeats.[c] $k_d = k_{obs}/(2 \times [cat]_0)$, assuming 2 active chains per metal centre. [d] TOF = moles of PVL consumed/time x moles of catalyst. Determined from 0 – 30% conversion or from conversion at 5 h.

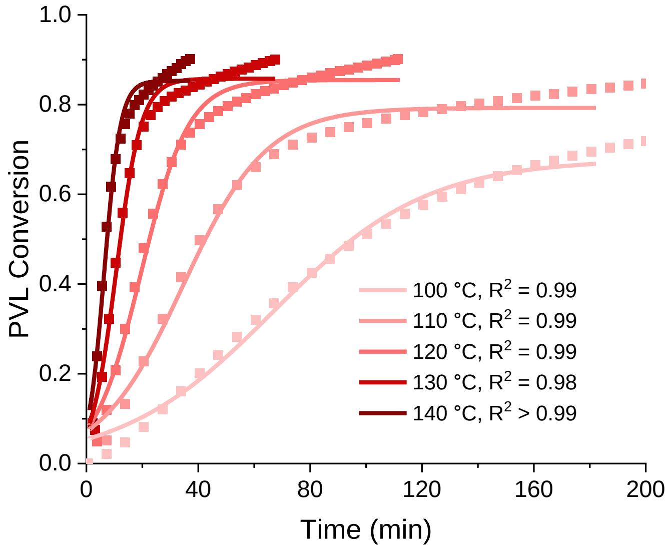

Figure S30. Plots of polymer conversion *vs* time and kinetic fits for PVL depolymerization catalysed by $Co(Oct)_2$ at 100, 110, 120, 130 and 140 °C. Experimental conditions: 1: 1000 $Co(Oct)_2$:PVL, for 5 h or until >95% conversion

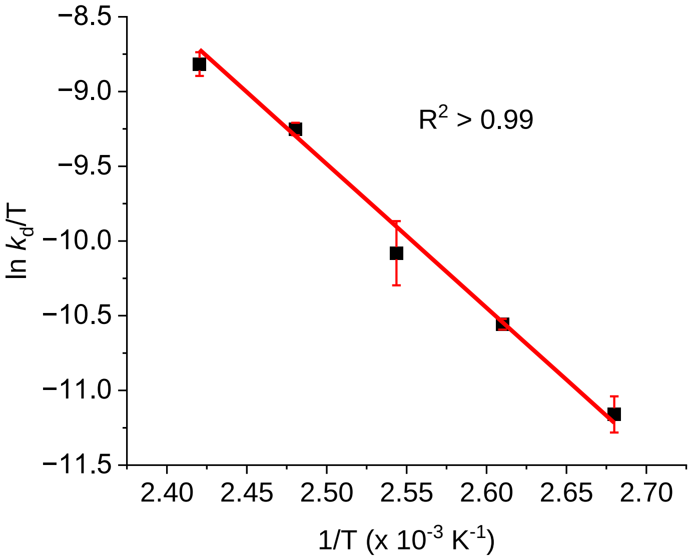

Figure S31. Plot of ln (k_d/T) *vs* 1/T for the recycling of PVL. Reactions were performed at 100, 110, 120, 130 and 140 °C with loadings of $[Co(Oct)_2]_0$: $[PVL]_0$ of 1:1000. $[Co(Oct)_2]_0$ = 9.98 x10^{-3}M

Table S12. Depolymerization Kinetic Data for PVL at 130 – 170 °C with 1:1000 $[Mg(Oct)_2]_0$: $[PVL]_0$ loading

Entry	Temp. (°C)	k[a] (min^{-1})	a[a]	xc[a] (min)	$ka/4$ ($\times 10^{-5} s^{-1}$)	k_{obs}[b] ($\times 10^{-5} s^{-1}$)	k_d[c] ($\times 10^{-3}$ $mol^{-1} dm^3 s^{-1}$)	TOF[d] (h^{-1})
1	130	0.0208	0.46	148.52	3.94	3.8 ± 0.2	1.9 ± 0.1	95 ± 4
		0.0196	0.47	154.66	3.80			
		0.0187	0.46	164.49	3.55			
2	140	0.0192	0.72	87.46	5.75	6.6 ± 0.6	3.3 ± 0.3	270± 30
		0.0190	0.72	86.69	5.68			
		0.0251	0.66	90.72	6.92			
3	150	0.0413	0.87	49.53	15.0	13 ± 2	6.3 ± 0.9	560± 60
		0.0309	0.83	61.63	10.7			
		0.0333	0.87	56.12	12.0			
4	160	0.0764	0.84	25.92	26.8	28 ± 1	14 ± 1	1030 ±40
		0.0806	0.85	27.09	28.4			
		0.0823	0.85	27.01	29.0			
5	170	0.249	0.88	10.11	90.8	83 ± 13	41 ± 7	2400 ±120
		0.255	0.88	10.91	93.0			
		0.185	0.83	11.59	63.9			

Reaction conducted by solvent casting $Mg(Oct)_2$:PVL solutions ($[Mg(Oct)_2]_0$: $[PVL]_0$ = 1: 1000, $[Mg(Oct)_2]_0$ = 9.98 x 10^{-3} M in bulk polymer) in TGA crucibles. N_2 flow = 25 mL min^{-1}.[a] Determined from logistic fitting of conversion *vs* time profiles from 0 – 90% mass loss or from 0 – 5 h.[b] k_{obs} = average of repeats listed, error = standard deviation from repeats.[c] $k_d = k_{obs}/(2 \times [cat]_0)$, assuming 2 active chains per metal centre. [d] TOF = moles of PVL consumed/time x moles of catalyst. Determined from 0 – 30% conversion or from conversion at 5 h.

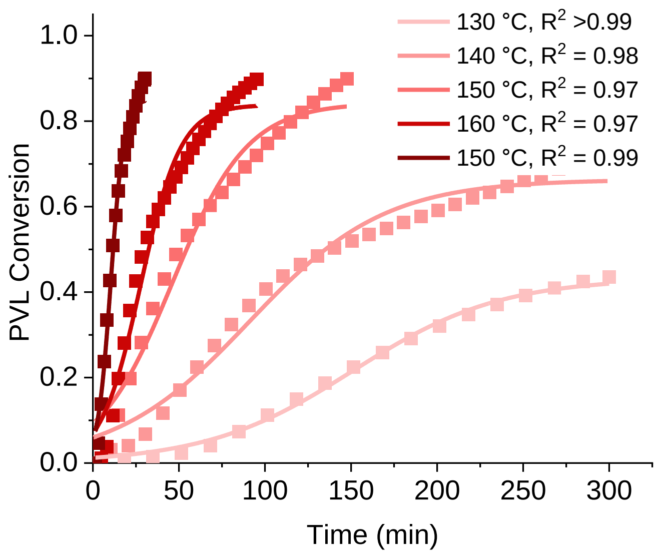

Figure S32. Plots of polymer conversion *vs* time and kinetic fits for PVL depolymerization catalysed by $Mg(Oct)_2$ at 100, 110, 120, 130 and 140 °C. Experimental conditions: 1: 1000 $Mg(Oct)_2$:PVL, for 5 h or until >95% conversion.

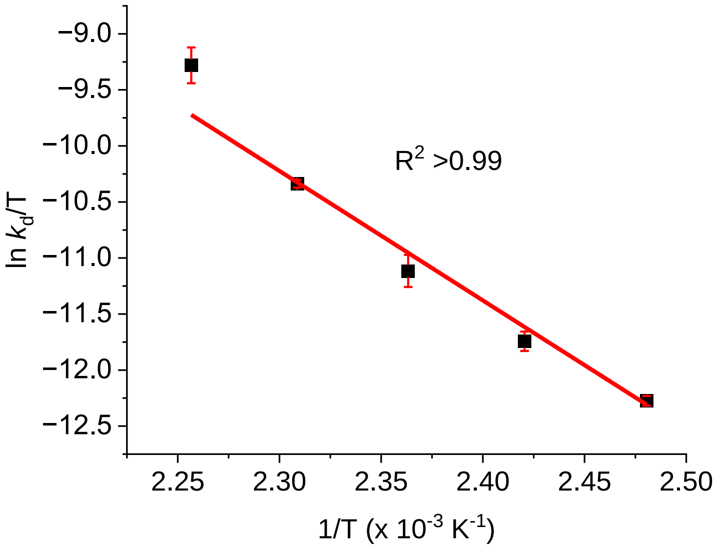

Figure S33. Plot of ln (k_d/T) *vs* 1/T for the recycling of PVL. Reactions were performed at 130, 140, 150, 160 and 170 °C with loadings of $[Mg(Oct)_2]_0$: $[PVL]_0$ of 1:1000. $[Mg(Oct)_2]_0$ = 9.98 x10^{-3} M

Table S13. Depolymerization Kinetic Data for PVL at 130 – 170 °C with 1:1000 $[Sn(Oct)_2]_0$: $[PVL]_0$ loading

Entry	Temp. (°C)	k[a] (min^{-1})	a[a]	xc[a] (min)	$ka/4$ (x 10^{-5} s^{-1})	k_{obs} [b] (x 10^{-5} s^{-1})	k_d [c] (x 10^{-3} mol^{-1} $dm^3 s^{-1}$)	TOF[d] (h^{-1})
1	130	0.0149	0.28	82.19	1.76	1.9E ± 0.1	0.96 ± 0.07	60 ± 10
		0.0147	0.34	91.76	2.09			
		0.0194	0.23	79.55	1.88			
2	140	0.0163	0.24	62.86	1.64	2.5 ± 0.8	1.2 ± 0.4	110 ± 10
		0.0205	0.39	66.16	3.36			
		0.0195	0.42	76.54	3.37			
3	150	0.0149	0.51	85.77	3.15	3.0 ± 0.5	1.5 ± 0.2	130 ± 25
		0.0236	0.36	67.74	3.56			
		0.0174	0.33	43.40	2.40			
4	160	0.0128	0.69	81.52	3.70	3.5 ± 0.3	1.8 ± 0.2	250 ± 40
		0.0210	0.44	57.59	3.84			
		0.0150	0.49	62.01	3.08			
5	170	0.0129	0.69	88.83	3.72	3.8 ± 0.5	1.9 ± 0.2	360 ± 90
		0.0153	0.69	68.60	4.38			
		0.0122	0.64	81.54	3.26			

Reaction conducted by solvent casting $Mg(Oct)_2$:PVL solutions ($[Sn(Oct)_2]_0$: $[PVL]_0$ = 1: 1000, $[Sn(Oct)_2]_0$ = 9.98 x 10^{-3} M in bulk polymer) in TGA crucibles. N_2 flow = 25 mL min^{-1}.[a] Determined from logistic fitting of conversion *vs* time profiles from 0 – 90% mass loss or from 0 – 5 h.[b] k_{obs} = average of repeats listed, error = standard deviation from repeats.[c] k_d = k_{obs}/(2 x $[cat]_0$), assuming 2 active chains per metal centre. [d] TOF = moles of PVL consumed/time x moles of catalyst. Determined from 0 – 30% conversion or from conversion at 5 h.

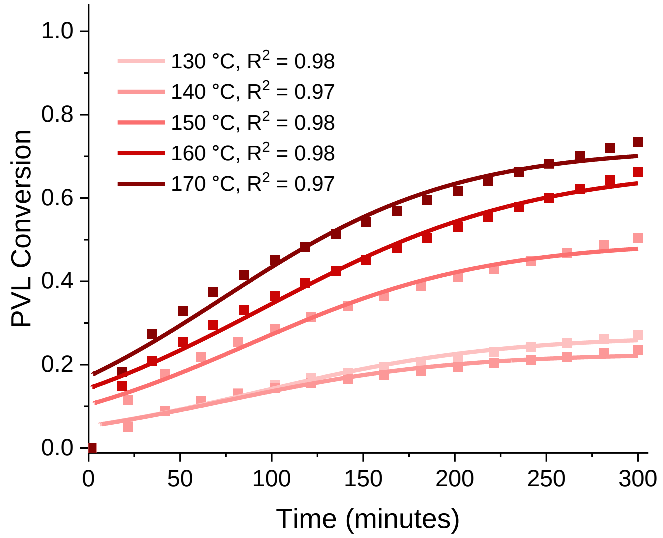

Figure S34. Plots of polymer conversion *vs* time and kinetic fits for PVL depolymerization catalysed by $Sn(Oct)_2$ at 100, 110, 120, 130 and 140 °C. Experimental conditions: 1: 1000 $Sn(Oct)_2$:PVL, for 5 h or until >95% conversion

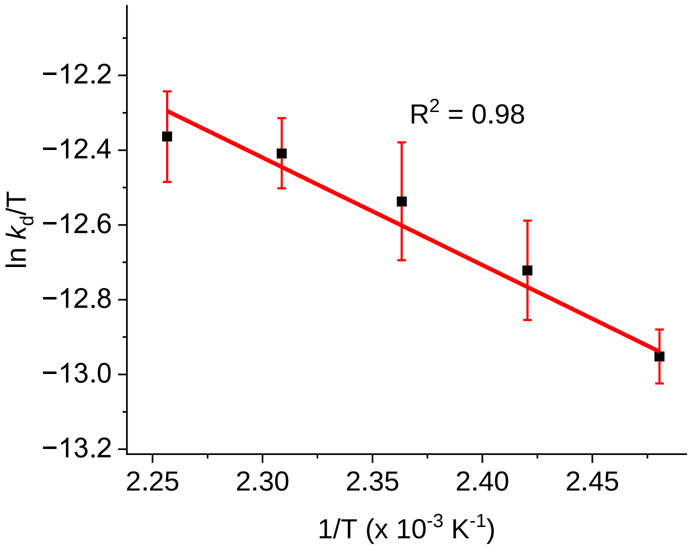

Figure S35. Plot of ln (k_d/T) *vs* 1/T for the recycling of PVL. Reactions were performed at 130, 140, 150, 160 and 170 °C with loadings of $[Sn(Oct)_2]_0$: $[PVL]_0$ of 1:1000. $[Sn(Oct)_2]_0$ = 9.98 x10^{-3}M

Table S14. Summary of Eyring data for depolymerization of PVL using loadings of 1:1000 $[M(Oct)_n]_0$: $[PVL]_0$

Entry	Metal	y = mx + c[a]	R^{2}[a]	$\Delta H^{\ddagger}$ [b] (kJ mol^{-1})	$\Delta S^{\ddagger}$ [c] (J K^{-1} mol^{-1})	$\Delta G^{\ddagger}$ [d] (kJ mol^{-1})
1	Zn(II)	-7453 ± 568<br>10.4 ± 1.4	0.98722	62.0 ± 4.7	-111 ± 15	107 ± 8
2	Co(II)	- 9623 ± 365<br>14.6 ± 0.9	0.9957	80.0 ± 3.0	76 ± 5	111 ± 4
3	Mg(II)	- 11550 ± 606<br>16.3 ± 1.4	0.99181	96.0 ± 5.0	62 ± 5	121 ± 5
4	Sn(II)	-2875 ± 254<br>9.8 ± 1.6	0.97698	23.9 ± 2.1	245 ± 25	123 ± 10

[a] Taken from Figs. S30 – 35 and reference[5]. [a] $\Delta H^{\ddagger}$ = - m x 8.314. [b] $\Delta S^{\ddagger}$ = 8.314 (c - ln (k_b/h)) where k_b = Boltzmann constant, h = planck constant [c] $\Delta G^{\ddagger}$ = $\Delta H^{\ddagger}$ - 403.14 x $\Delta S^{\ddagger}$.

4.4 DFT

Density Functional theory (DFT) calculations were performed using Gaussian16 suite of codes (revision C.01).[14] Geometries were fully optimised without any symmetry or geometry constraints. The nature of all the stationary points as minima or transition states (first-order saddle points) on the potential energy surface was verified by calculations of the vibrational frequency spectrum. Geometry optimisations were carried out using the PBE0 functional with empirical dispersion correction factor, GD3, applied.[15] The 6-31+G(d) basis set was used for O atoms and the 6-31G(d,p) basis set was used for C and H atoms.[16] The SDD pseudopotential was used for Zn and MG atoms and the solvent effects were modelled using conductor-like polarizable continuum model in ethyl acetate.[17] Goodvibes software[18] was used to apply temperature and concentration correction factors to calculated free enthalpies at 403.14 K and $[Zn]_0$ and $[Mg]_0$ = 9.98 x 10^{-3} M for methyl 5-hydroxypentanoate.

Table S15. Computed intermediates and transition states for the backbiting of methyl 5-hydroxypentanoate and methyl 6-hydroxyhexanoate

Structure	G (Hartree)	G temperature corrected (Hartree)[a]	$\Delta G^{\ddagger}$ (kJ mol^{-1})
**Zn(II) Methyl 5-hydroxypentanoate ring closure**			
i	-1147.728022	-1147.751872	0
TS1	-1147.717291	-1147.740207	30.6
ii	-1147.722559	-1147.745081	17.8
iii	-1147.725723	-1147.748227	9.6
TS2	-1147.716685	-1147.739657	32.1
iv	-1147.732621	-1147.755667	-10.0
**Mg(II) Methyl 5-hydroxypentanoate ring closure**			
i'	-1120.701494	-1120.724638	0
TS1’	-1120.688372	-1120.711501	34.5
ii'	-1120.700527	-1120.723489	3.0
iii’	-1120.695917	-1120.71884	15.2
TS2’	-1120.690808	-1120.713107	30.3
iv'	-1120.707054	-1120.729761	-13.5

[a]Temperature correction using Goodvibes software

5. 2,2-Dimethylenecarbonate (DTC), polymer and large-scale recycled monomer characterisation

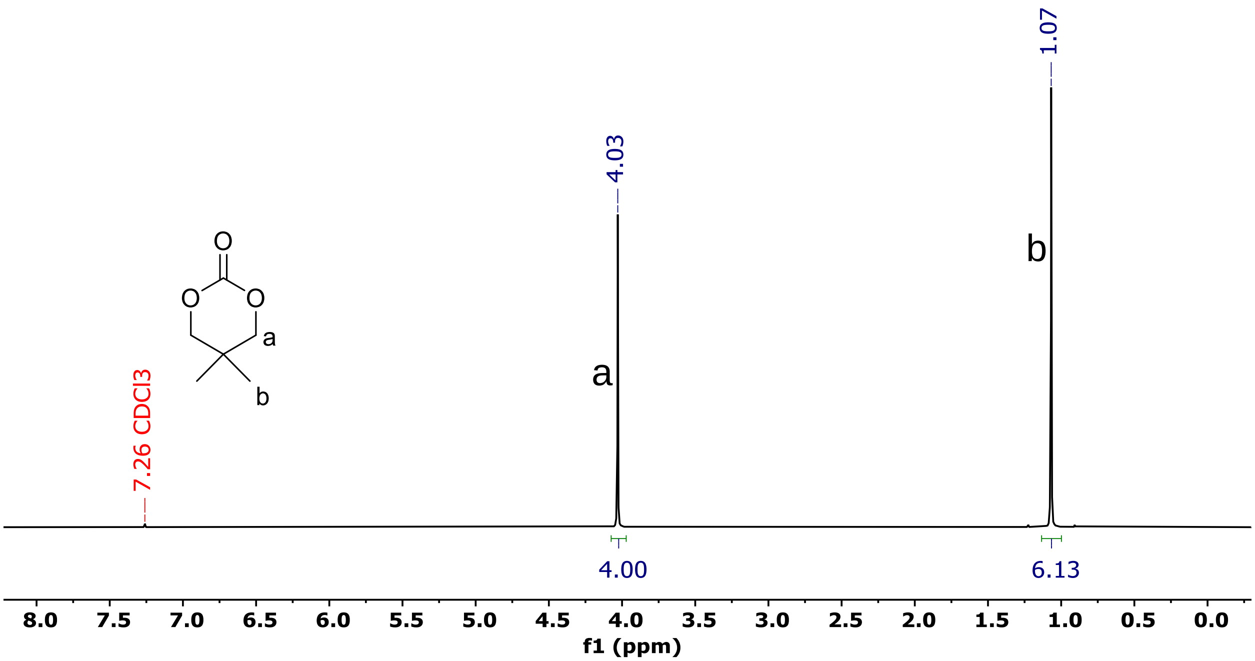

Figure S36. ^{1}H NMR spectrum (400 MHz, $CDCl_3$, 298K) of DTC.

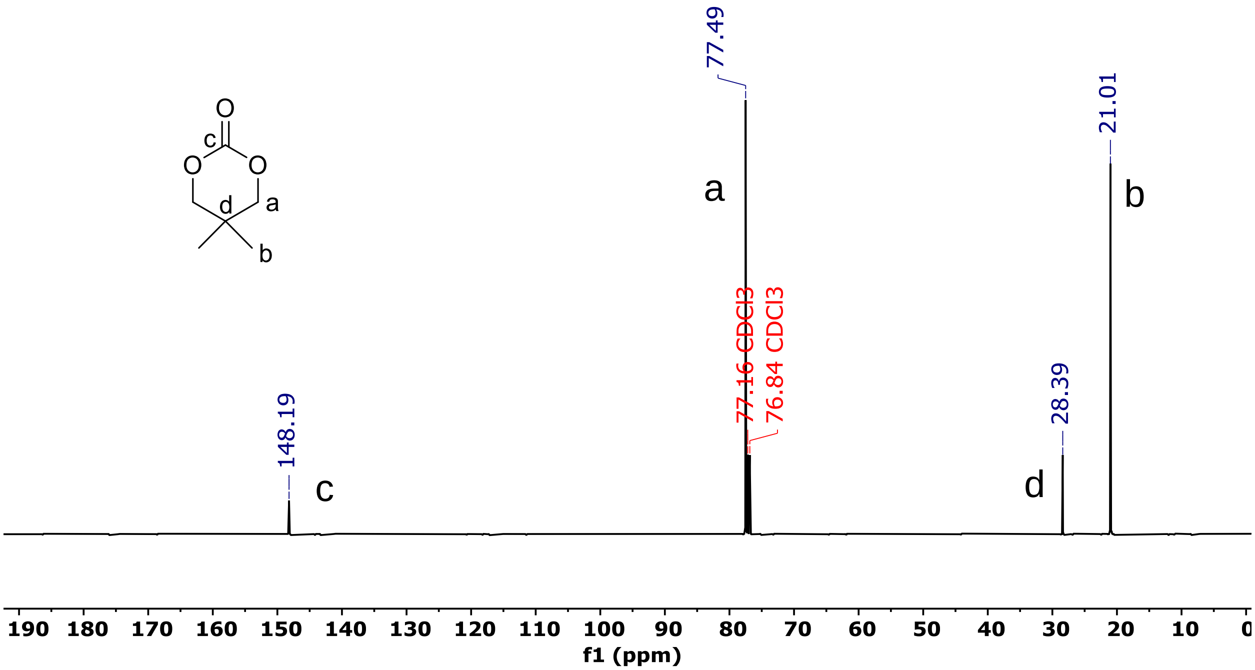

Figure S37. ^{13}C{^{1}H} NMR spectrum (101 MHz, $CDCl_3$, 298K) of DTC.

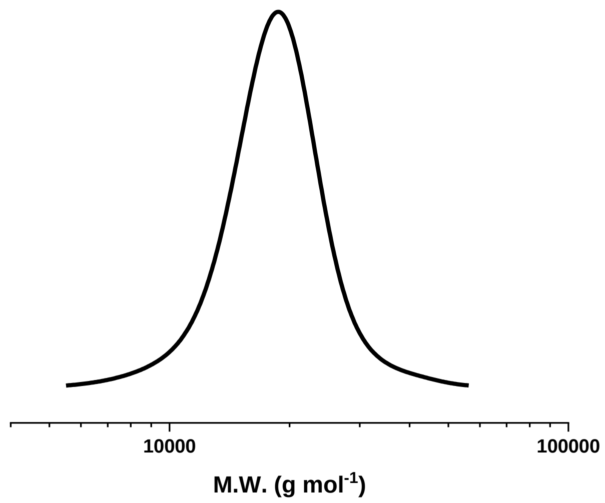

Figure S38. SEC trace of PVL. $M_{n,SEC}$ = 17,500 g mol $^{-1}$, $Đ_M$ = 1.09. Reproduced from,[5] available under CC-BY 4.0 licence. Copyright McGuire et al 2025.

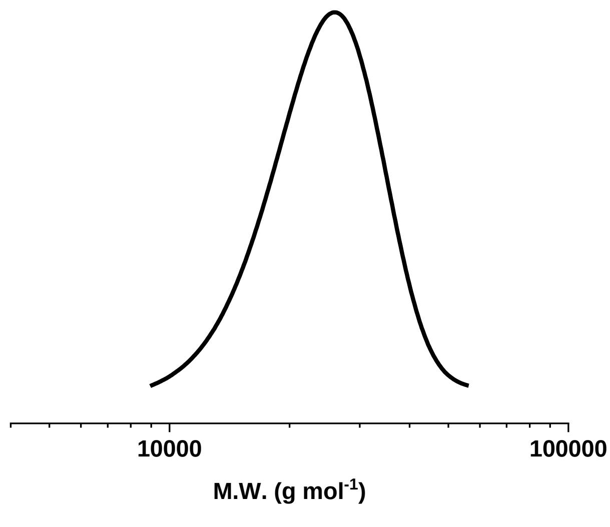

Figure S39. SEC trace of P3MeVL. $M_{n,SEC}$ = 23,100 g mol $^{-1}$, $Đ_M$ = 1.10. Reproduced from,[5] available under CC-BY 4.0 licence. Copyright McGuire et al 2025.

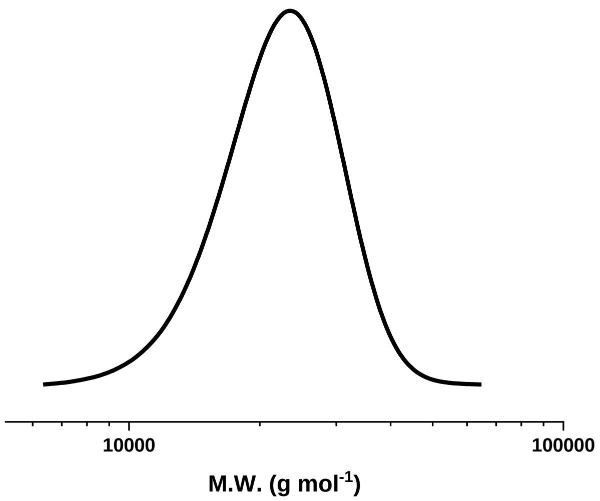

Figure S40. SEC trace of PHL. $M_{n,SEC}$ = 21,000 g mol $^{-1}$, $Đ_M$ = 1.10. Reproduced from,[5] available under CC-BY 4.0 licence. Copyright McGuire et al 2025.

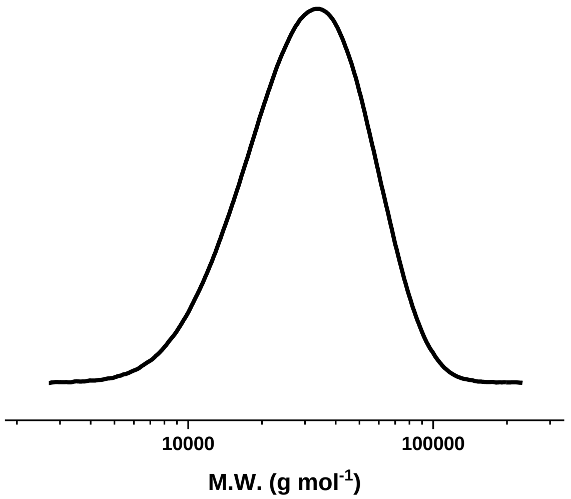

Figure S41. SEC trace of PCL. $M_{n,SEC}$ = 24,900 g mol $^{-1}$, $Đ_M$ = 1.39. Reproduced from,[5] available under CC-BY 4.0 licence. Copyright McGuire et al 2025.

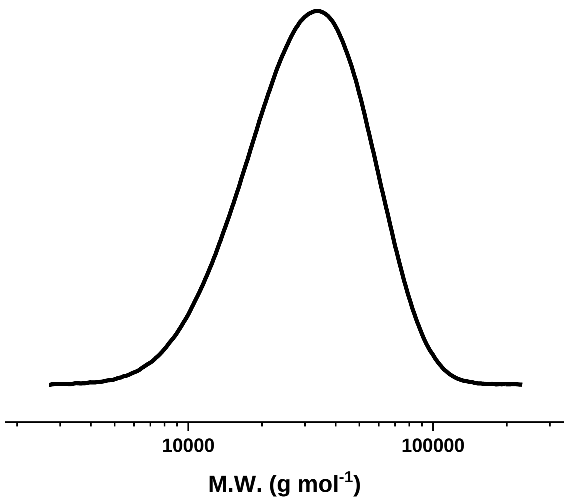

Figure S42. SEC trace of P4MeCL. $M_{n,SEC}$ = 22,800 g mol $^{-1}$, $Đ_M$ = 1.39. Reproduced from,[5] available under CC-BY 4.0 licence. Copyright McGuire et al 2025.

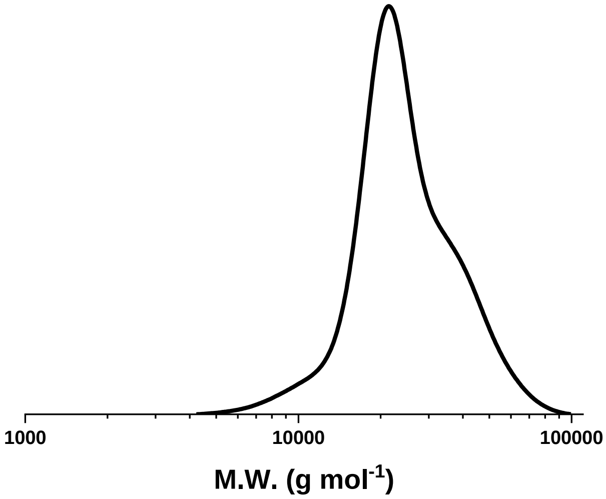

Figure S43. SEC trace of PDTC. $M_{n,SEC}$ = 22,200 g mol^{-1}, $Đ_M$ = 1.20. Reproduced from,[5] available under CC-BY 4.0 licence. Copyright McGuire et al 2025.

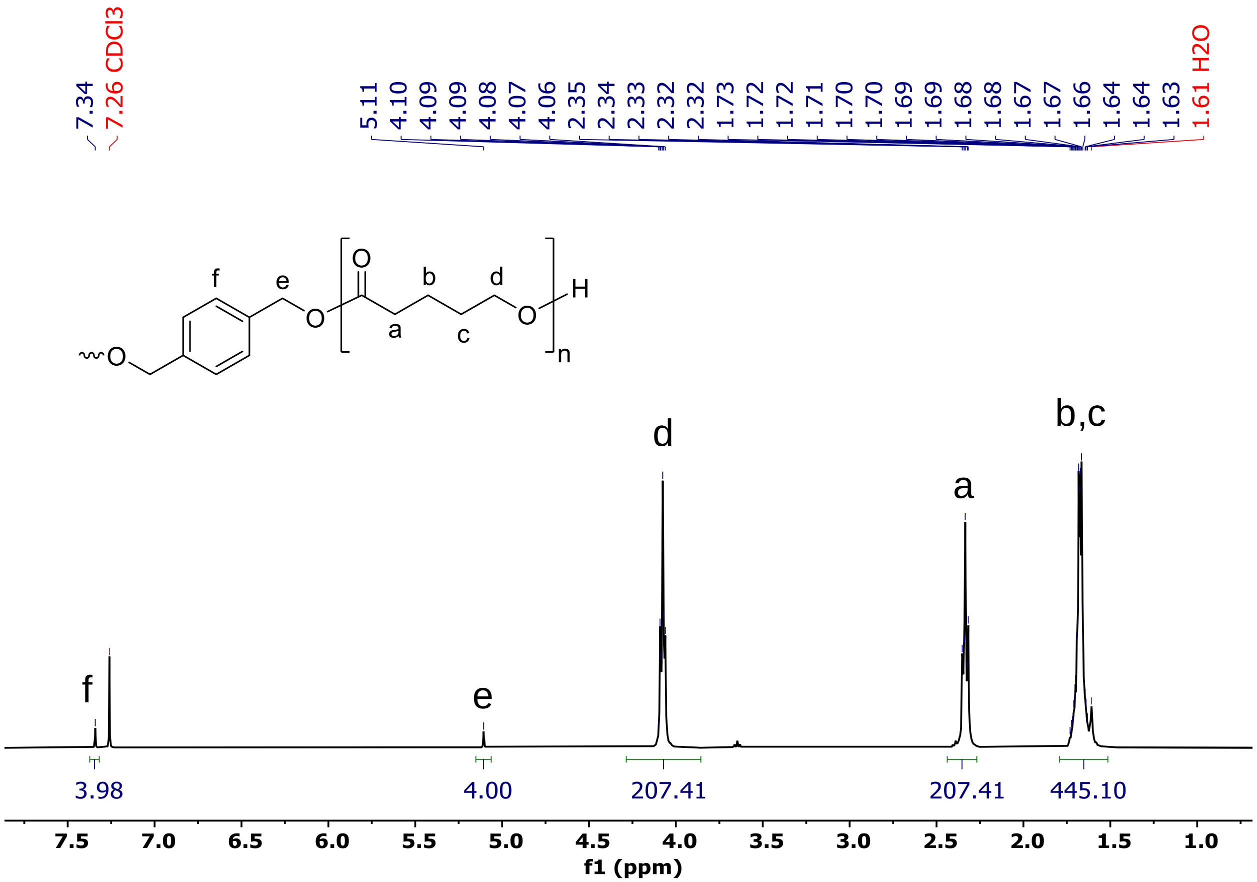

Figure S44. ^{1}H NMR spectrum (400 MHz, $CDCl_3$, 298K) of PVL. DP_{NMR} =104 determined from relative integration of H_d and H_e. Reproduced from,[5] available under CC-BY 4.0 licence. Copyright McGuire et al 2025.

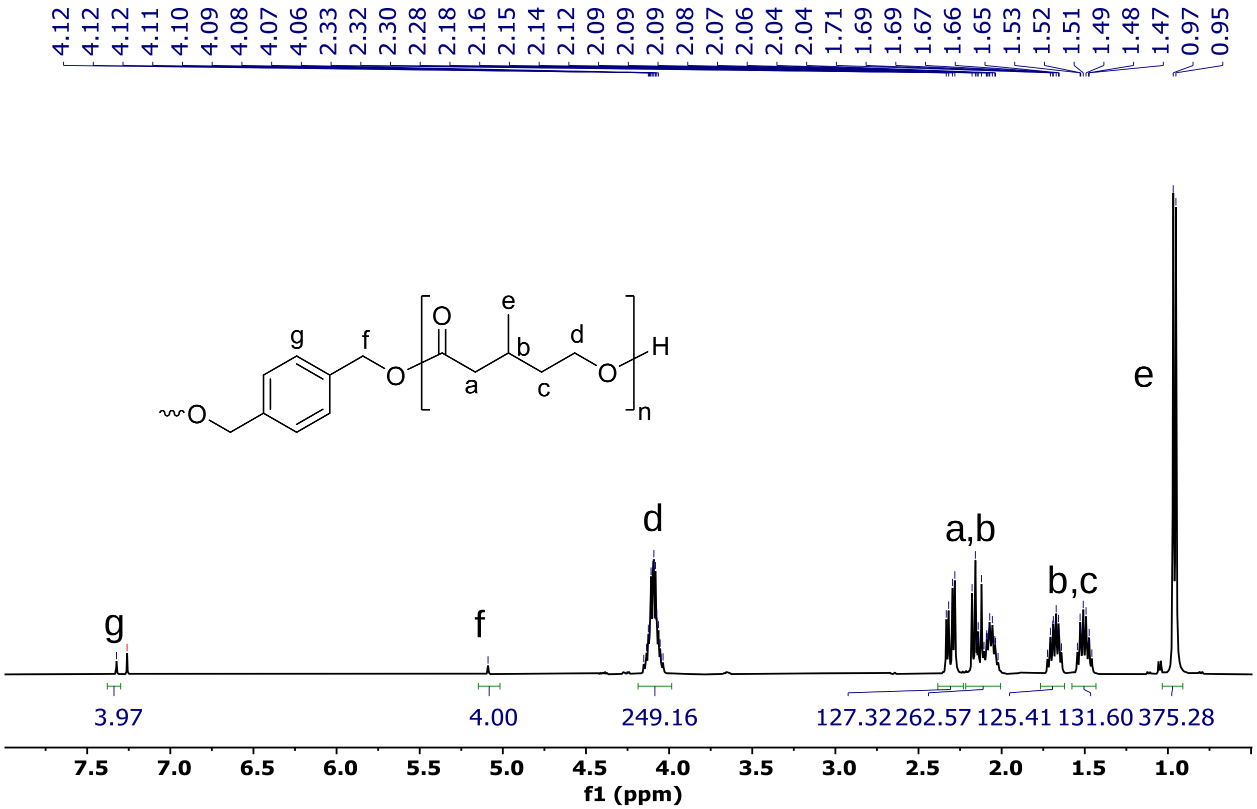

Figure S45. **^{1}H NMR spectrum (400 MHz, $CDCl_3$, 298K) of P3MeVL. DP_{NMR} =125 determined from relative integration of H_d and H_f. Reproduced from,[5] available under CC-BY 4.0 licence. Copyright McGuire et al 2025.**

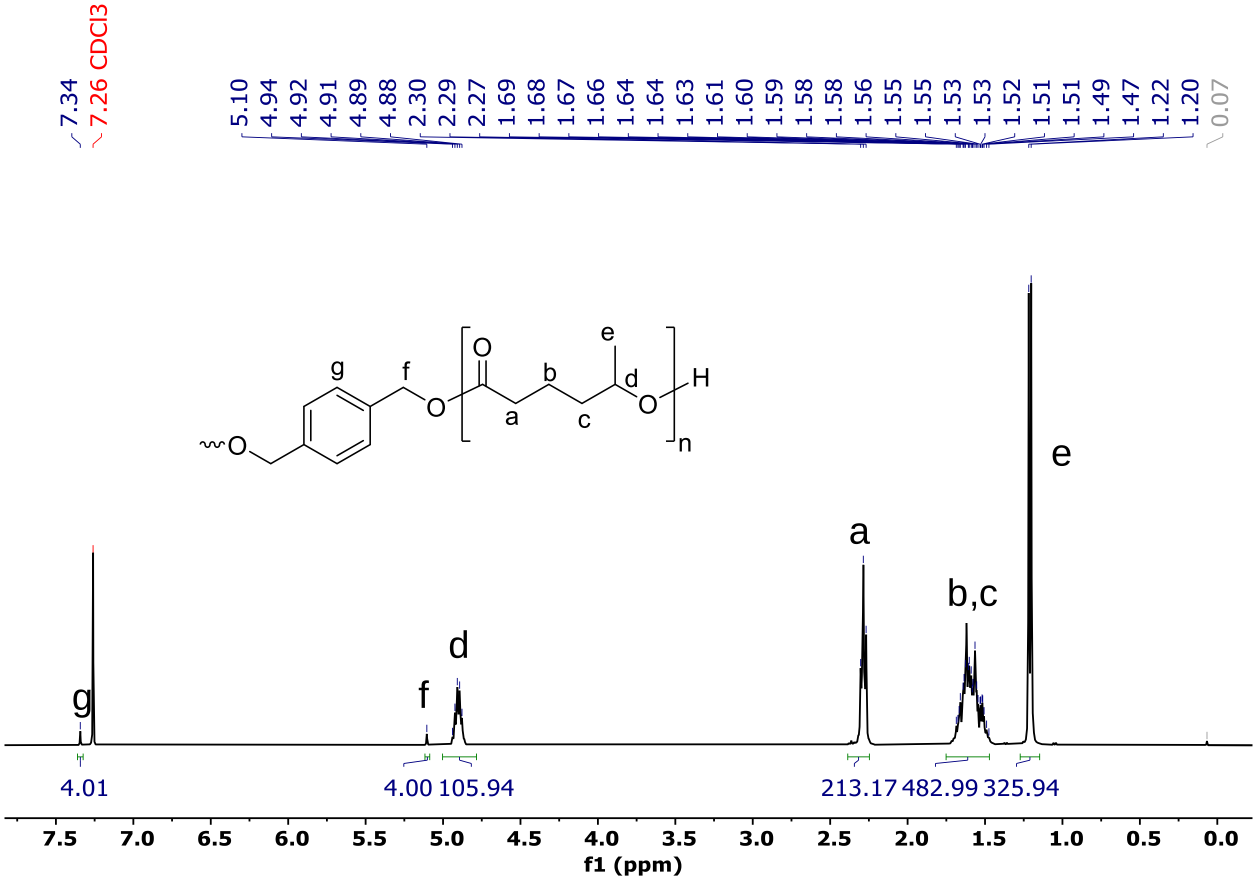

Figure S46. ^{1}H NMR spectrum (400 MHz, $CDCl_3$, 298K) of PHL. DP_{NMR} =106 determined from relative integration of H_d and H_f. Reproduced from,[5] available under CC-BY 4.0 licence. Copyright McGuire et al 2025.

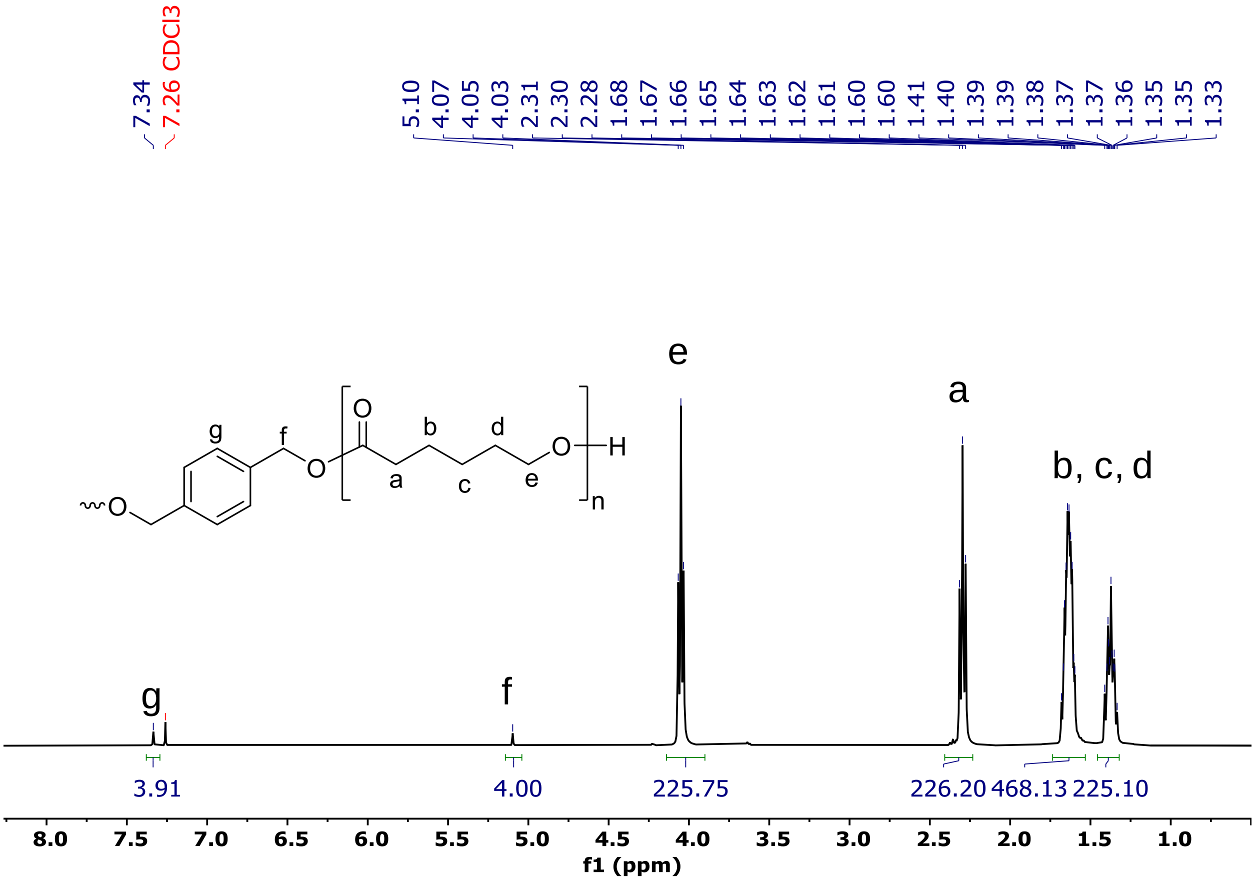

Figure S47. ^{1}H NMR spectrum (400 MHz, $CDCl_3$, 298K) of PCL. DP_{NMR} =112 determined from relative integration of H_f and H_e. Reproduced from,[5] available under CC-BY 4.0 licence. Copyright McGuire et al 2025.

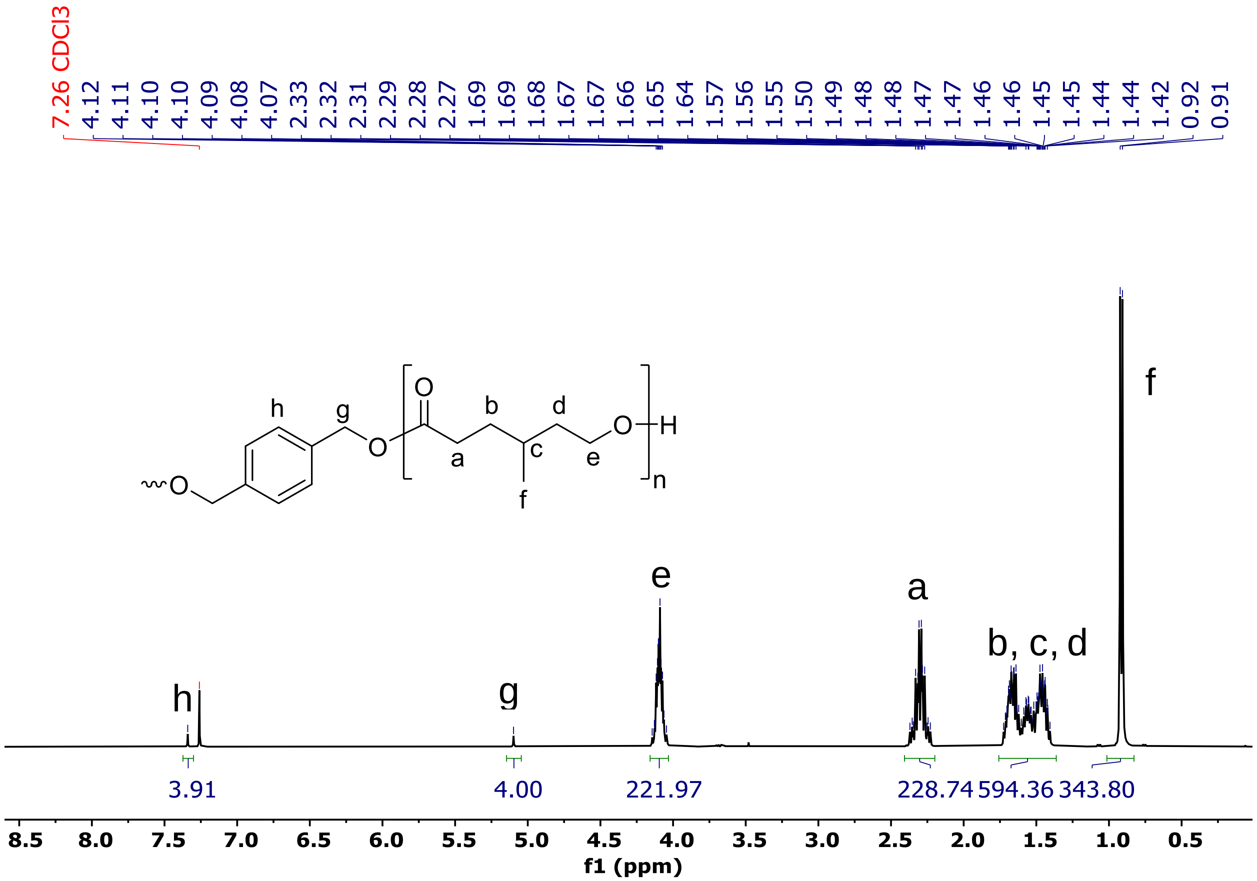

Figure S48. ^{1}**H NMR spectrum (400 MHz,** $CDCl_3$**, 298K) of P4MeCL.** DP_{NMR} **=111 determined from the relative integral of** H_e **and** H_g**. Reproduced from,[5] available under CC-BY 4.0 licence. Copyright McGuire et al 2025.**

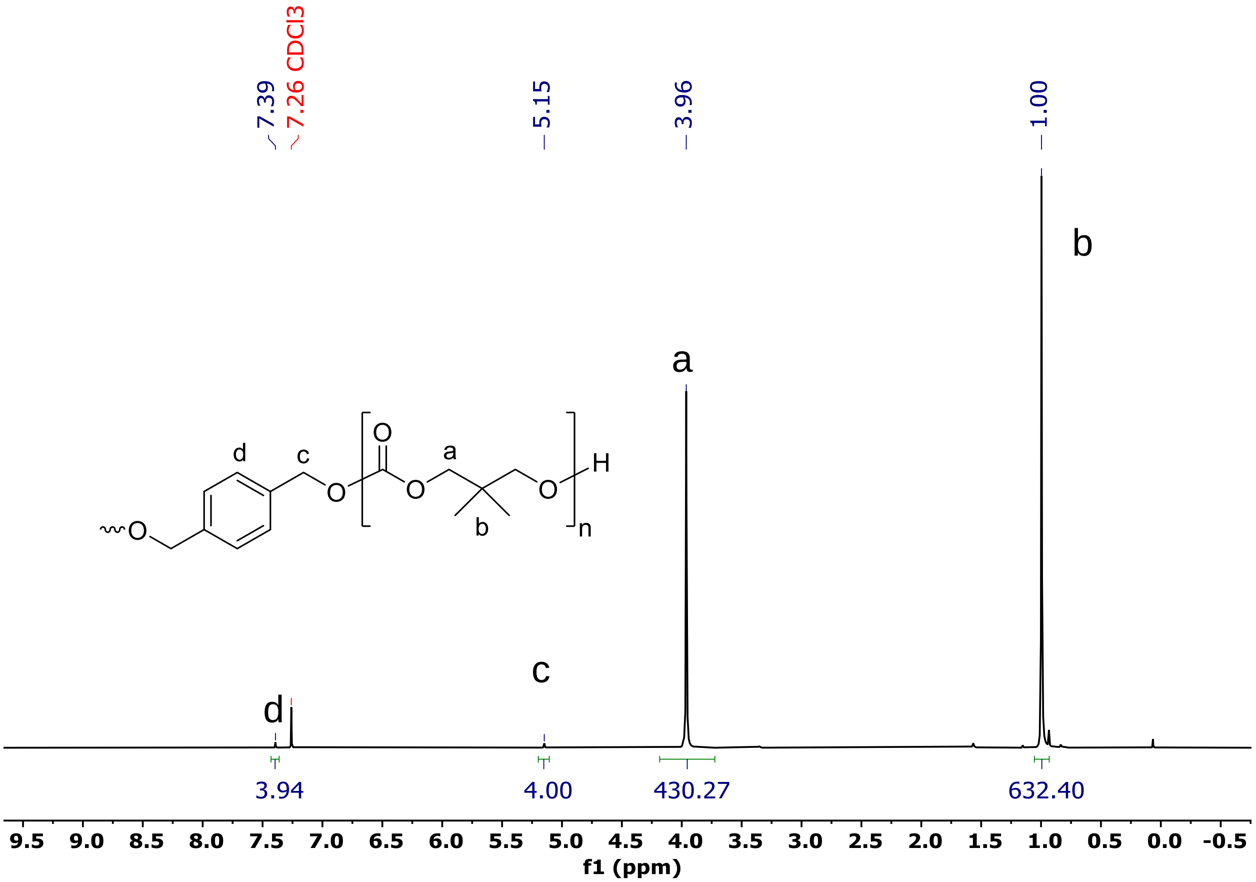

Figure S49. **^{1}H NMR spectrum (400 MHz, $CDCl_3$, 298K) of PDTC. DP_{NMR} =108 determined from relative integration of H_c and H_a. Reproduced from,[5] available under CC-BY 4.0 licence. Copyright McGuire et al 2025.**

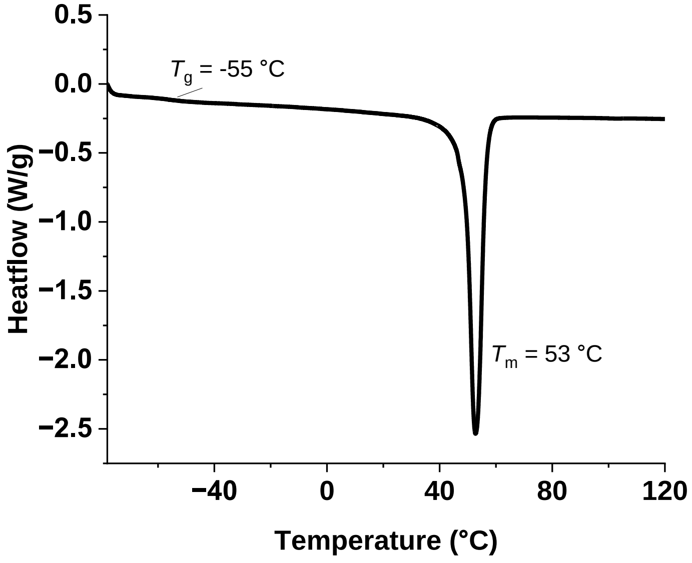

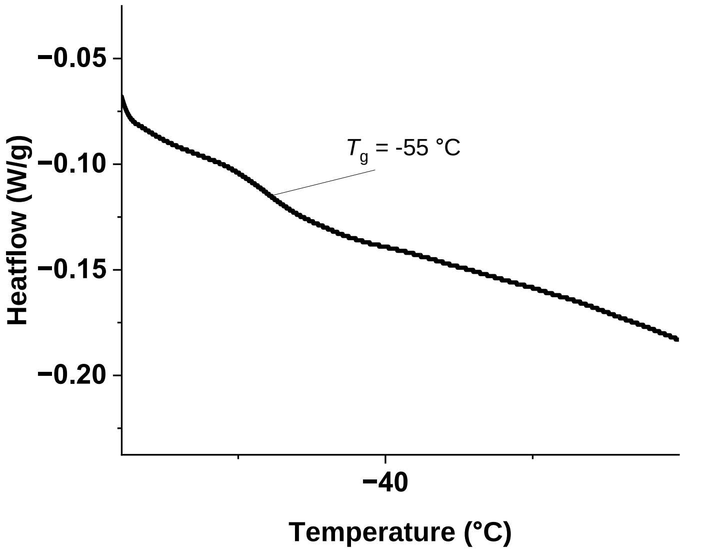

Figure S50. DSC thermograms (2nd heating cycle) of PVL T_g = -55 °C, T_m = 53 °C. Reproduced from,[5] available under CC-BY 4.0 licence. Copyright McGuire et al 2025.

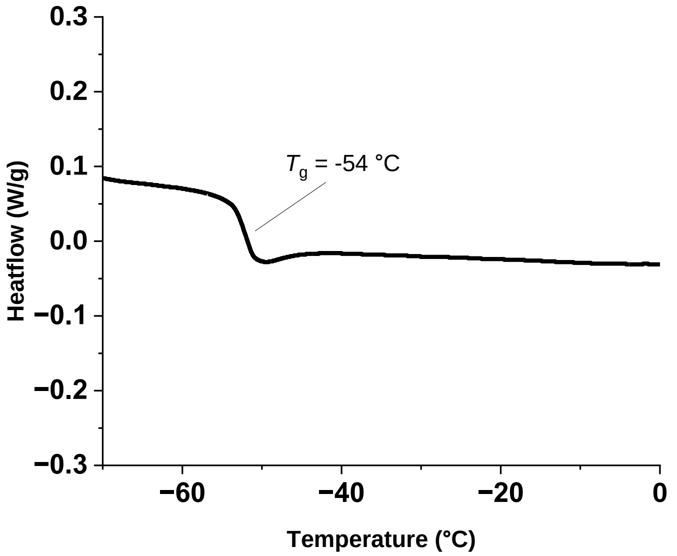

Figure S51. **DSC thermogram (2nd heating cycle) of P3MeVL, T_g = -54 °C, T_m = *not observed*. Reproduced from,[5] available under CC-BY 4.0 licence. Copyright McGuire et al 2025.**

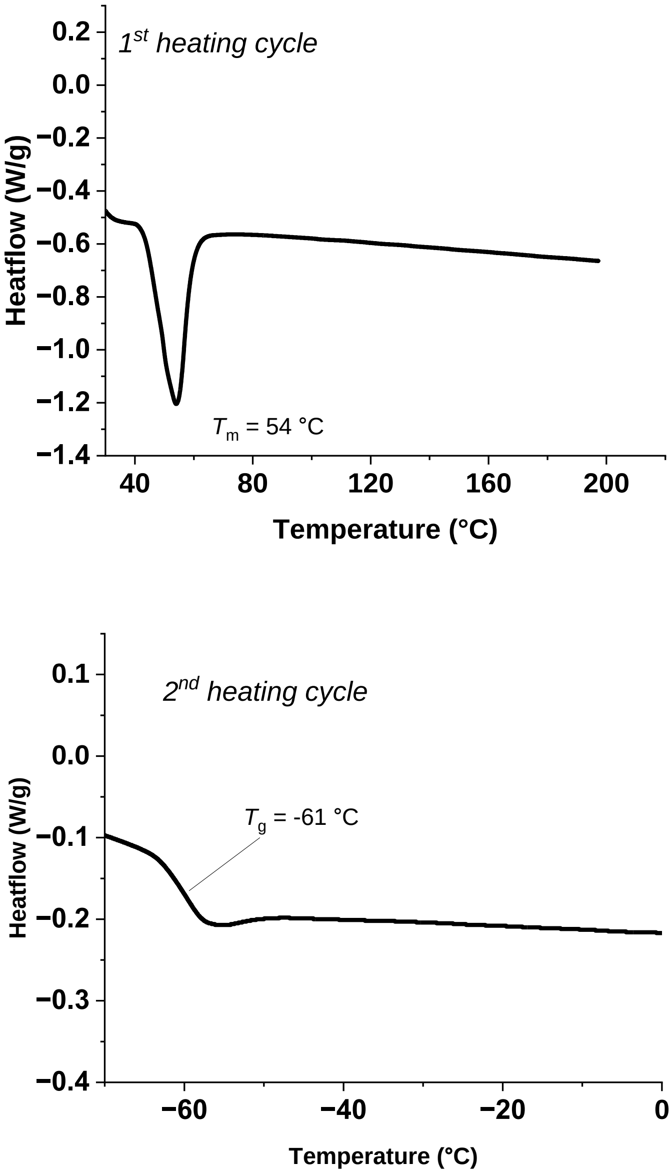

Figure S52. DSC thermograms of PHL, T_g = -61 °C, T_m (2nd heating cycle) = *not observed.* T_m (first cycle) = 54 °C. Reproduced from,[5] available under CC-BY 4.0 licence. Copyright McGuire et al 2025.

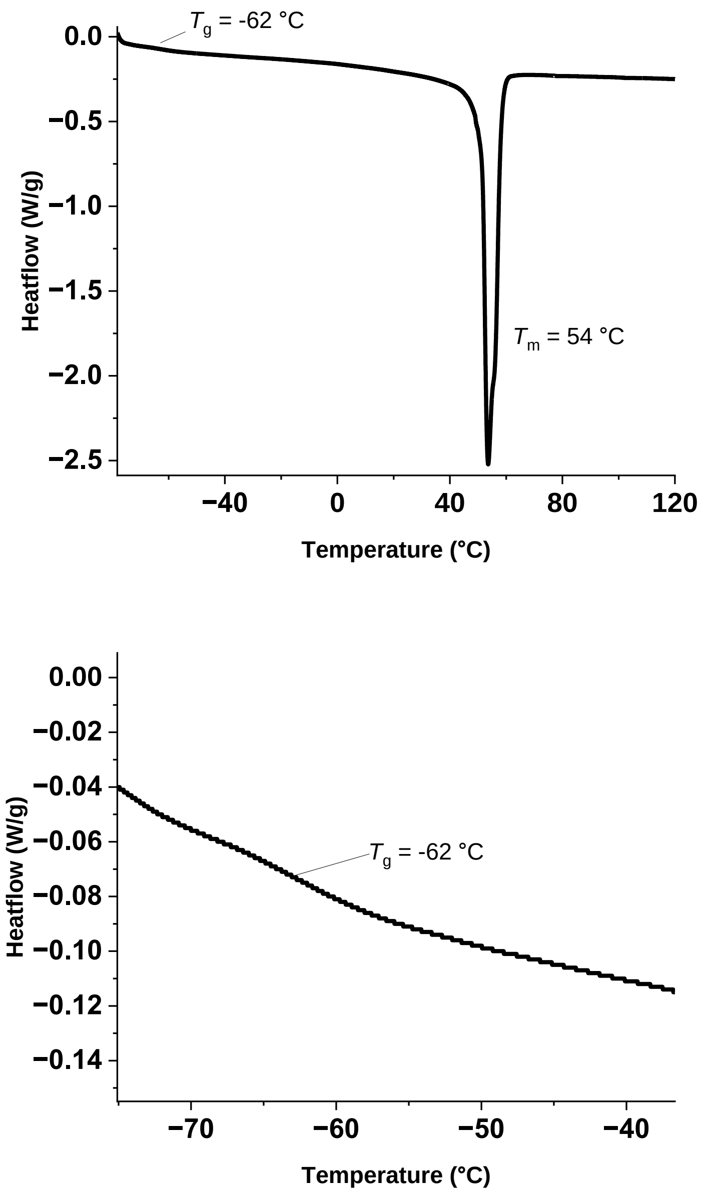

Figure S53. DSC thermograms (2nd heating cycle) of PCL, T_g = -61 °C, T_m = 53 °C. Reproduced from,[5] available under CC-BY 4.0 licence. Copyright McGuire et al 2025.

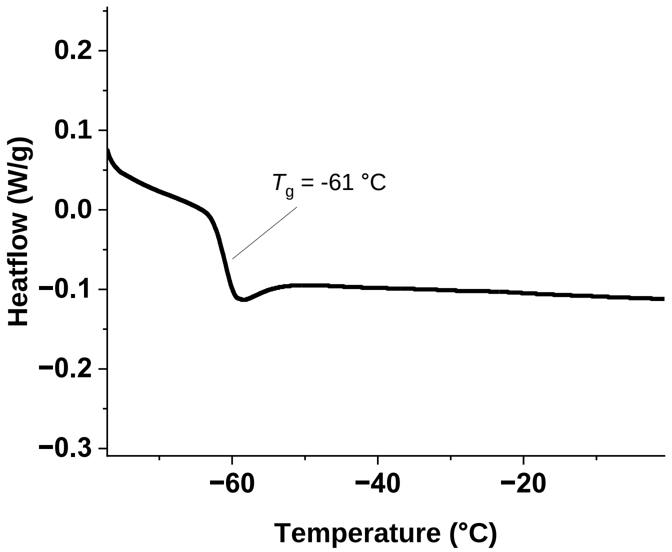

Figure S54. DSC thermogram (2nd heating cycle) of P4MeCL, T_g = -61 °C, T_m =*not observed*. Reproduced from,[5] available under CC-BY 4.0 licence. Copyright McGuire et al 2025.

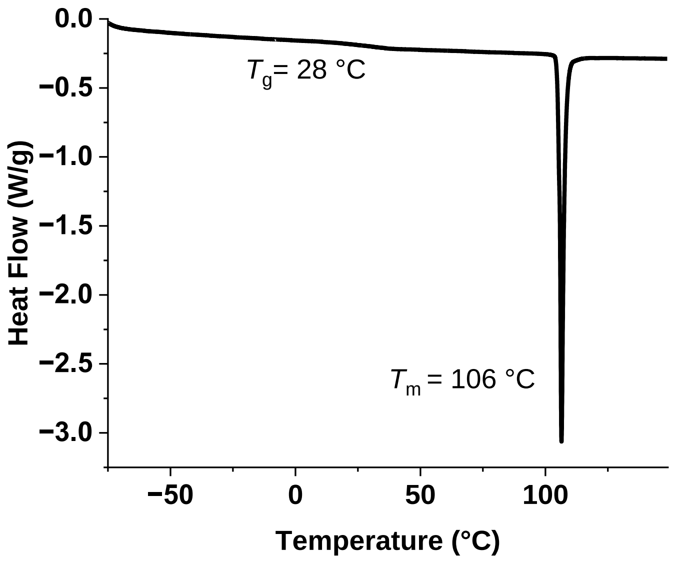

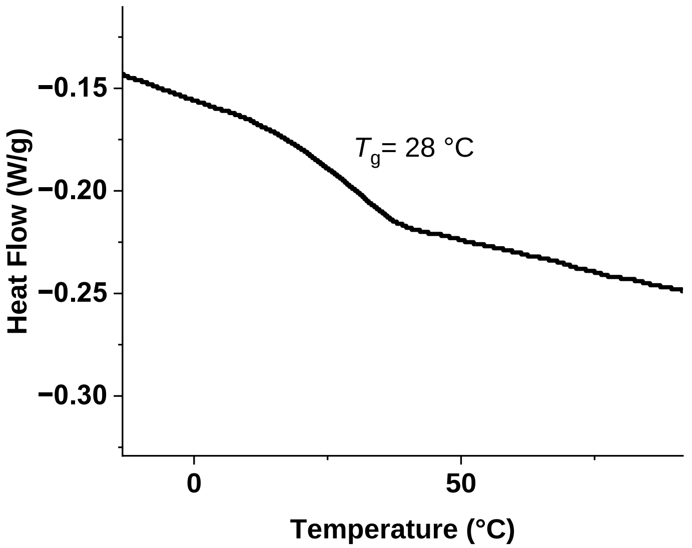

Figure S55. DSC thermograms (2nd heating cycle) of PDTC, T_g = 28 °C, T_m = 106 °C. Reproduced from,[5] available under CC-BY 4.0 licence. Copyright McGuire et al 2025.

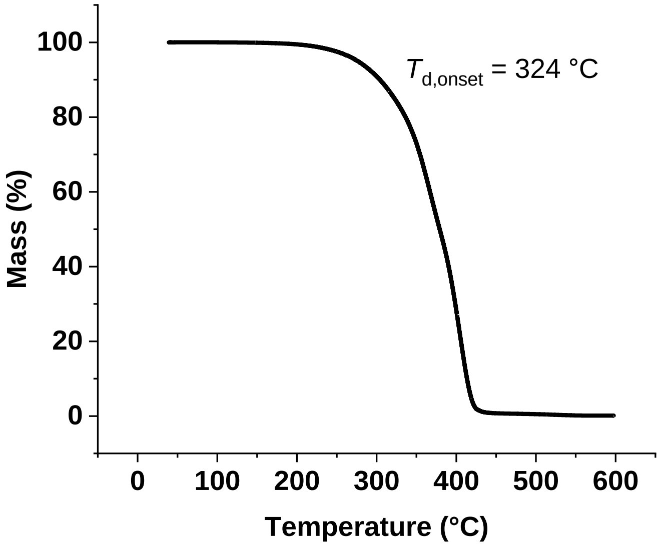

Figure S56. TGA thermogram of PVL, $T_{d,onset}$ = 324 °C. Reproduced from,[5] available under CC-BY 4.0 licence. Copyright McGuire et al 2025.

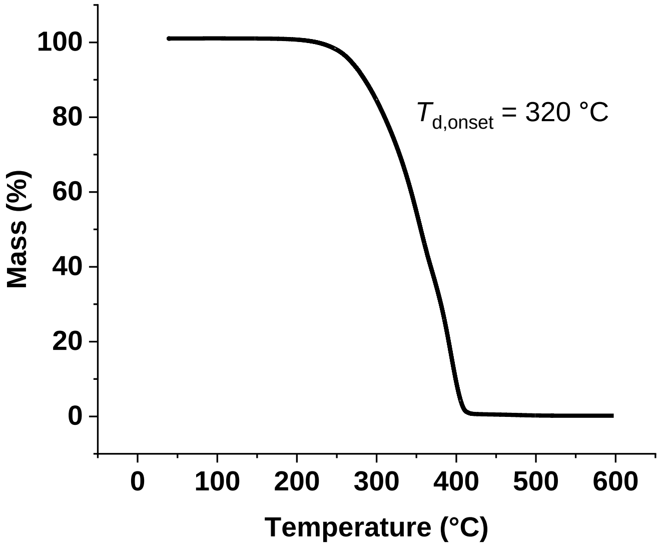

Figure S57. TGA thermogram of P3MeVL, $T_{d,onset}$ = 320 °C. Reproduced from,[5] available under CC-BY 4.0 licence. Copyright McGuire et al 2025.

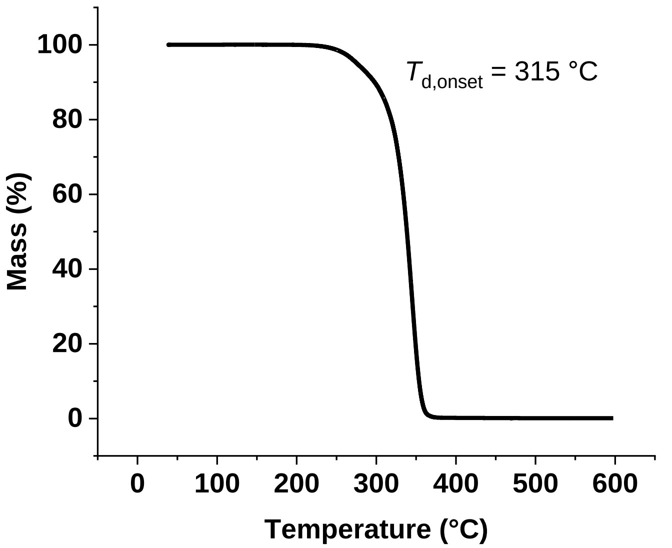

Figure S58. TGA thermogram of PHL, $T_{d,onset}$ = 315 °C. Reproduced from,[5] available under CC-BY 4.0 licence. Copyright McGuire et al 2025.

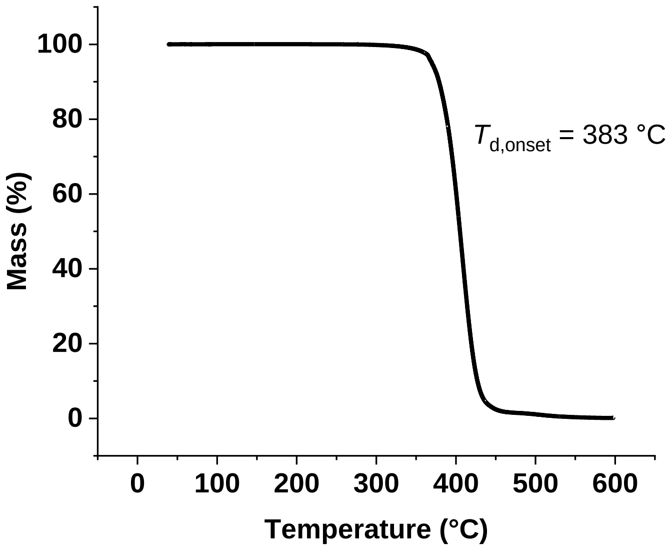

Figure S59. TGA thermogram of PCL, $T_{d,onset}$ = 383 °C. Reproduced from,[5] available under CC-BY 4.0 licence. Copyright McGuire et al 2025.

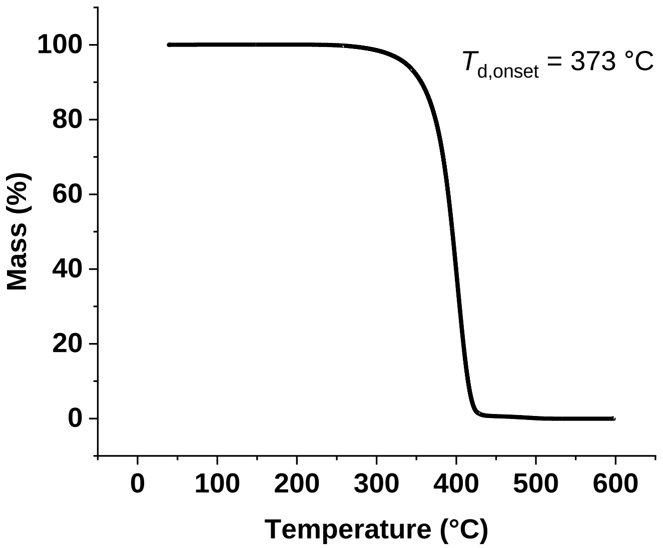

Figure S60. TGA thermogram of P4MeCL, $T_{d,onset}$ = 373 °C. Reproduced from,[5] available under CC-BY 4.0 licence. Copyright McGuire et al 2025.

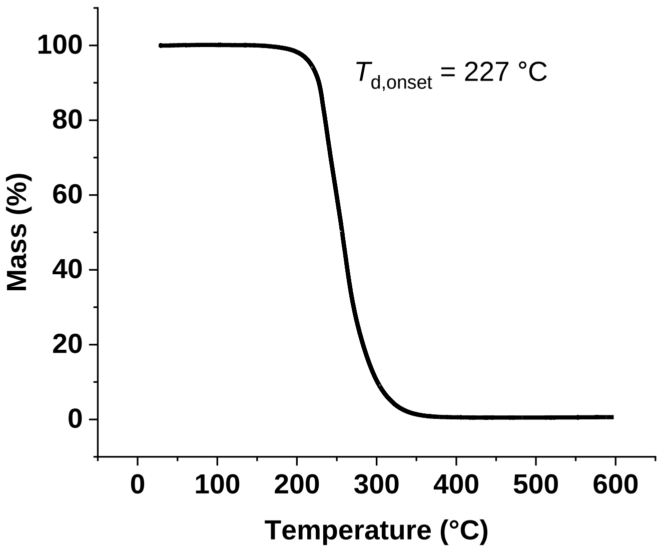

Figure S61. TGA thermogram of PDTC. $T_{d,onset}$ = 227 °C. Reproduced from,[5] available under CC-BY 4.0 licence. Copyright McGuire et al 2025.

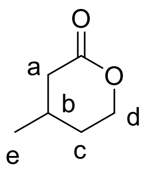

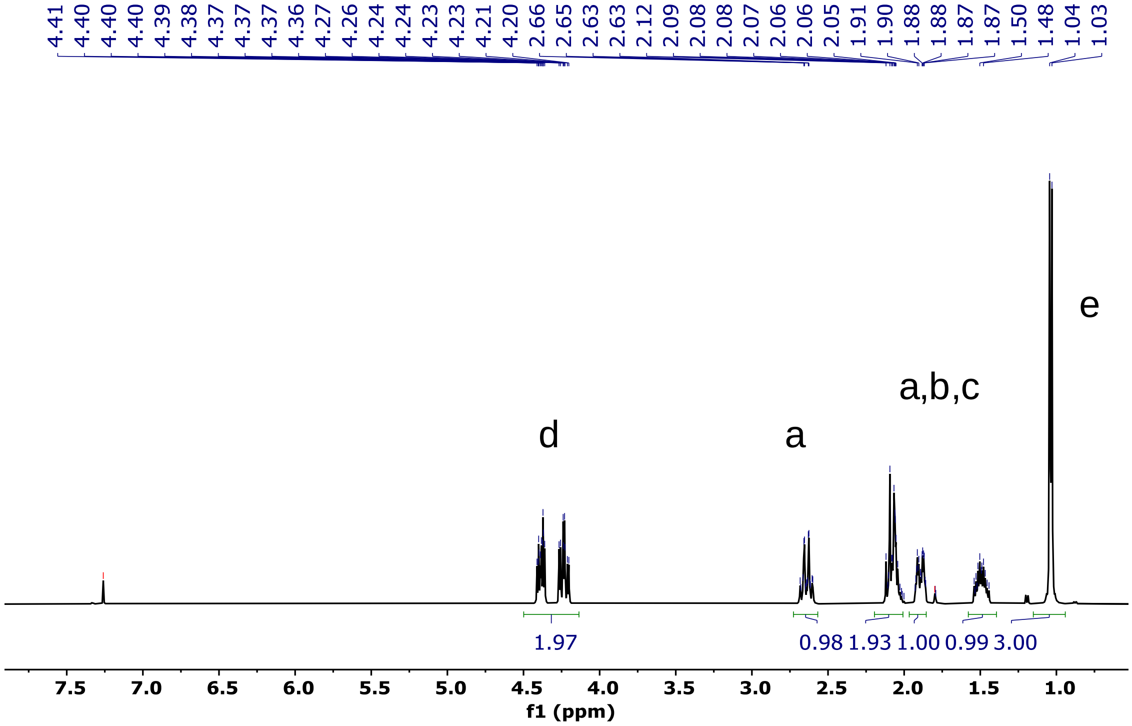

Figure S62. **^{1}H NMR spectrum (400 MHz, $CDCl_3$, 298K) of 3MeVL isolated from recycling of P3MeVL ($[Zn(Oct)_2]_0$:$[P3MeVL]_0$ 1:1000, 130 °C). Reproduced from,[5] available under CC-BY 4.0 licence. Copyright McGuire et al 2025.**

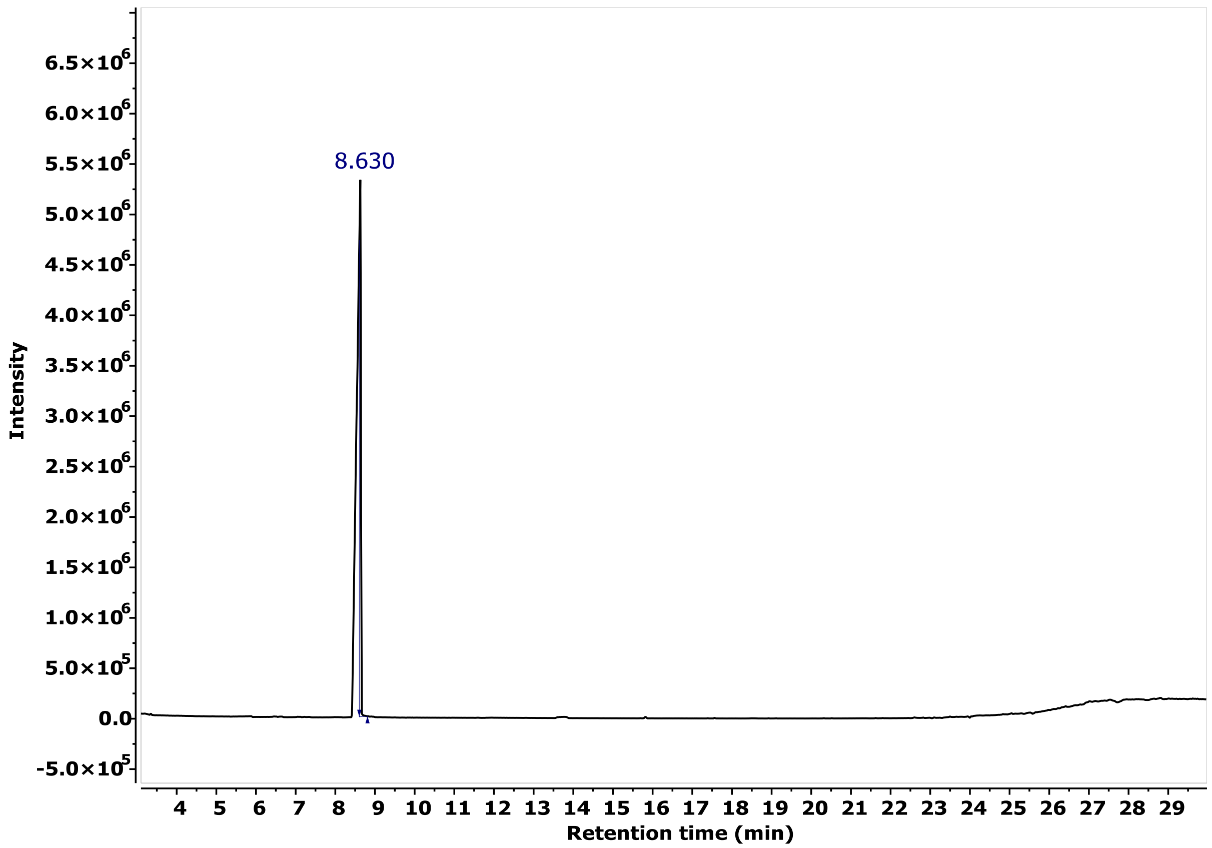

Figure S63. GC of of 3MeVL isolated from recycling of P3MeVL ($[Zn(Oct)_2]_0$:$[P3MeVL]_0$ 1:1000, 130 °C). Pure sample of 3MeVL elutes at 8.63 minutes. Reproduced from,[5] available under CC-BY 4.0 licence. Copyright McGuire et al 2025.

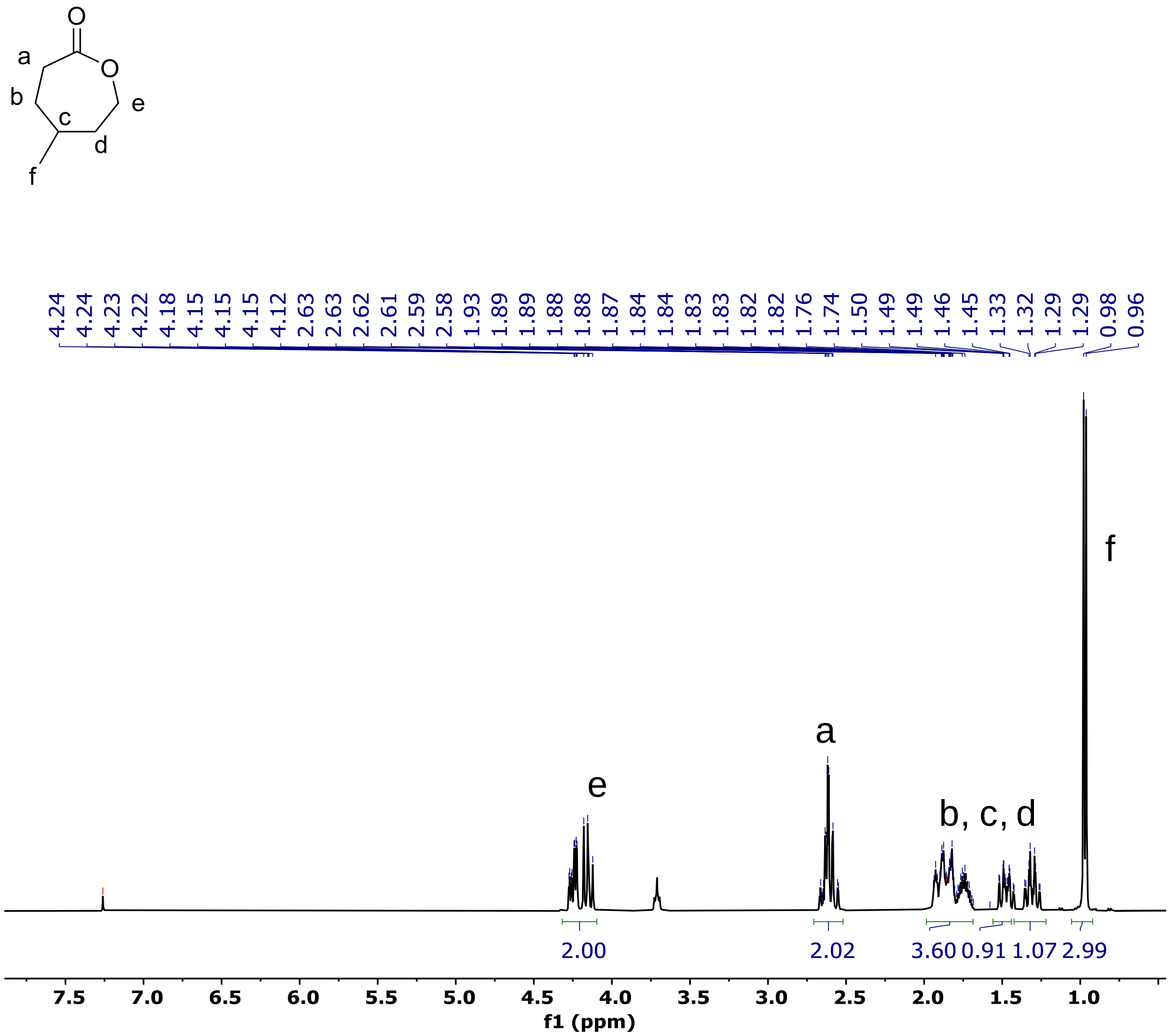

Figure S64. ^{1}H **NMR spectrum (400 MHz,** $CDCl_3$**, 298K) of 4MeCL isolated from recycling of P4MeCL ([Zn(Oct)$_2$]$_0$:[P4MeCL]$_0$ 1:100, 160 °C). Residual THF at δ = 3.75 ppm. Reproduced from,[5] available under CC-BY 4.0 licence. Copyright McGuire et al 2025.**

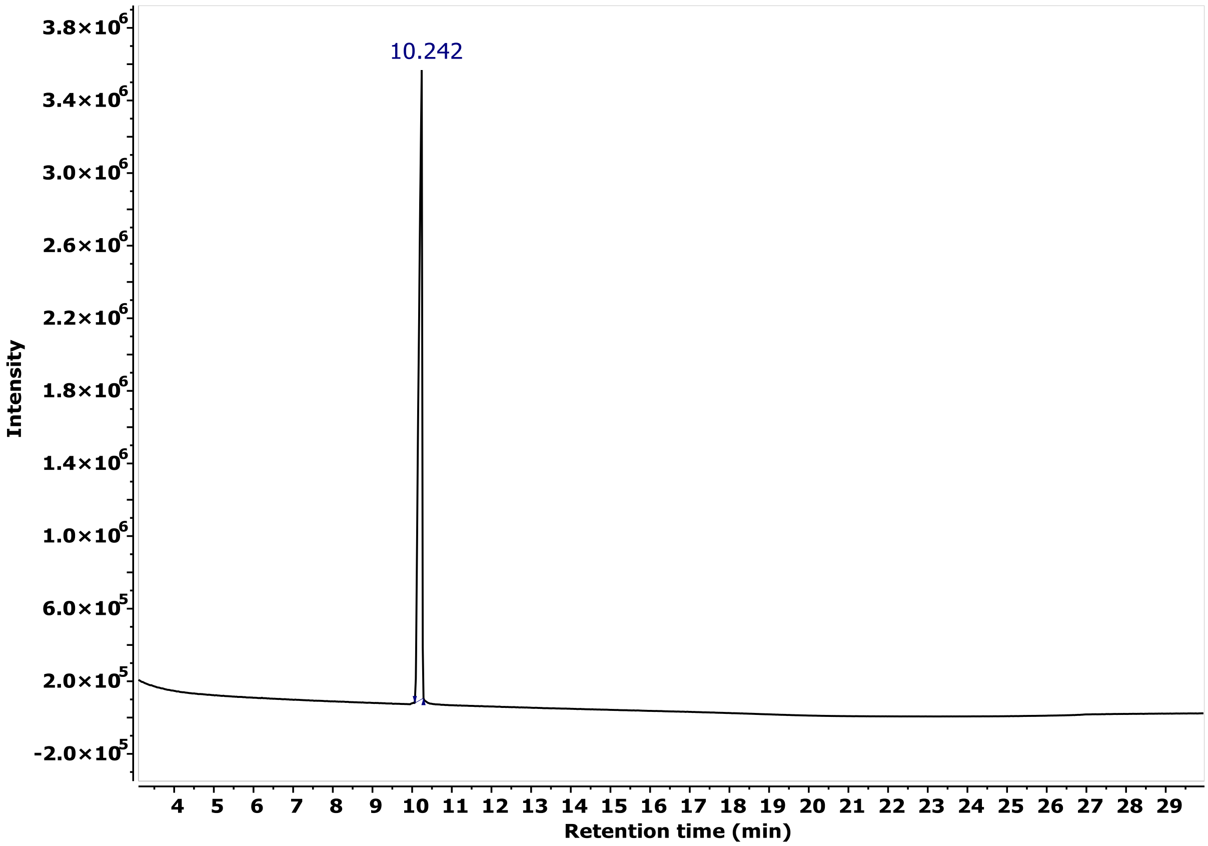

Figure S65. GC of 4MeCL isolated from recycling of P4MeCL ($[Zn(Oct)_2]_0$:$[4MeCL]_0$ 1:100, 160 °C). Reproduced from,[5] available under CC-BY 4.0 licence. Copyright McGuire et al 2025.

6. References

(1) Schneiderman, D. K.; Hillmyer, M. A. Aliphatic polyester block polymer design. *Macromolecules* **2016**, *49* (7), 2419–2428. DOI: 10.1021/acs.macromol.6b00211.

(2) Watts, A.; Kurokawa, N.; Hillmyer, M. A. Strong, resilient, and sustainable aliphatic polyester thermoplastic elastomers. *Biomacromolecules* **2017**, *18* (6), 1845–1854. DOI: 10.1021/acs.biomac.7b00283.

(3) Olsson, J. V.; Hult, D.; Cai, Y.; García-Gallego, S.; Malkoch, M. Reactive imidazole intermediates: simplified synthetic approach to functional aliphatic cyclic carbonates. *Polym. Chem.* **2014**, *5* (23), 6651–6655. DOI: https://doi.org/10.1039/C4PY00911H.

(4) Brown, P. L.; Ekberg, C. *Hydrolysis of metal ions*; John Wiley & Sons, 2016.

(5) McGuire, T. M.; Ning, D.; Buchard, A.; Williams, C. K. The Science of Polymer Chemical Recycling Catalysis: Uncovering Kinetic and Thermodynamic Linear Free Energy Relationships. *J. Am. Chem. Soc.* **2025**, *147* (26), 22734–22746. DOI: 10.1021/jacs.5c04603.

(6) Li, C.; Sablong, R. J.; van Benthem, R. A. T. M.; Koning, C. E. Unique Base-Initiated Depolymerization of Limonene-Derived Polycarbonates. *ACS Macro Lett.* **2017**, *6* (7), 684–688. DOI: 10.1021/acsmacrolett.7b00310.

(7) Shannon, R. D. Revised effective ionic radii and systematic studies of interatomic distances in halides and chalcogenides. *Foundations of Crystallography* **1976**, *32* (5), 751–767. DOI: https://doi.org/10.1107/S0567739476001551.

(8) Sidey, V. On the effective ionic radii for the tin(II) cation. *J. Phys. Chem. Solids* **2022**, *171*, 110992. DOI: https://doi.org/10.1016/j.jpcs.2022.110992.
(9) Kepp, K. P. A Quantitative Scale of Oxophilicity and Thiophilicity. *Inorg. Chem.* **2016**, *55* (18), 9461–9470. DOI: 10.1021/acs.inorgchem.6b01702.
(10) Yevstropov, A. A.; Lebedev, B. V.; Kulagina, T. G.; Lebedev, N. K. The calorimetric study in the 13·8–340 K range of δ-valerolactone, its polymer, and of the δ-valerolactone polymerization. *Polym. Sci. USSR* **1982**, *24* (3), 628–636. DOI: https://doi.org/10.1016/0032-3950(82)90053-3.
(11) Duda, A.; Kowalski, A.; Libiszowski, J.; Penczek, S. Thermodynamic and Kinetic Polymerizability of Cyclic Esters. *Macromol. Symp.* **2005**, *224* (1), 71–84. DOI: https://doi.org/10.1002/masy.200550607.
(12) McGuire, T. M.; Ning, D.; Williams, C. K. Using Differential Scanning Calorimetry to Accelerate Polymerization Catalysis: A Toolkit for Miniaturized and Automated Kinetics Measurements. *ACS Catal.* **2025**, *15* (9), 6760–6771. DOI: 10.1021/acscatal.5c01758.
(13) Olsén, P.; Odelius, K.; Albertsson, A.-C. Thermodynamic presynthetic considerations for ring-opening polymerization. *Biomacromolecules* **2016**, *17* (3), 699–709. DOI: 10.1021/acs.biomac.5b01698.
(14) Gaussian 16 Rev. C.01; Frisch, M. J.; Trucks, G. W.; Schlegel, H. B.; Scuseria, G. E.; Robb, M. A.; Cheeseman, J. R.; Scalmani, G.; Barone, V.; Petersson, G. A.; Nakatsuji, H.; Li, X.; Caricato, M.; Marenich, A. V.; Bloino, J.; Janesko, B. G.; Gomperts, R.; Mennucci, B.; Hratchian, H. P.; Ortiz, J. V.; Izmaylov, A. F.; Sonnenberg, J. L.; Williams-Young, D.; Ding, F.; Lipparini, F.; Egidi, F.; Goings, J.; Peng, B.; Petrone, A.; Henderson, T.; Ranasinghe, D.; Zakrzewski, V. G.; Gao, J.; Rega, N.; Zheng, G.; Liang, W.; Hada, M.; Ehara, M.; Toyota, K.; Fukuda, R.; Hasegawa, J.; Ishida, M.; Nakajima, T.; Honda, Y.; Kitao, O.; Nakai, H.; Vreven, T.; Throssell, K.; Montgomery, J. A., Jr.; Peralta, J. E.; Ogliaro, F.; Bearpark, M. J.; Heyd, J. J.; Brothers, E. N.; Kudin, K. N.; Staroverov, V. N.; Keith, T. A.; Kobayashi, R.; Normand, J.; Raghavachari, K.; Rendell, A. P.; Burant, J. C.; Iyengar, S. S.; Tomasi, J.; Cossi, M.; Millam, J. M.; Klene, M.; Adamo, C.; Cammi, R.; Ochterski, J. W.; Martin, R. L.; Morokuma, K.; Farkas, O.; Foresman, J. B.; Fox, D. J, Gaussian Inc, Wallingford, CT, 2016.
(15) Zhao, Y.; Truhlar, D. G. A new local density functional for main-group thermochemistry, transition metal bonding, thermochemical kinetics, and noncovalent interactions. *J. Chem. Phys.* **2006**, *125* (19). DOI: https://doi.org/10.1063/1.2370993.
(16) Hehre, W. J.; Ditchfield, R.; Pople, J. A. Self—consistent molecular orbital methods. XII. Further extensions of Gaussian—type basis sets for use in molecular orbital studies of organic molecules. *J. Chem. Phys.* **1972**, *56* (5), 2257–2261. DOI: https://doi.org/10.1063/1.1677527.
(17) Marenich, A. V.; Cramer, C. J.; Truhlar, D. G. Universal solvation model based on solute electron density and on a continuum model of the solvent defined by the bulk dielectric constant and atomic surface tensions. *J. Phys. Chem. B* **2009**, *113* (18), 6378–6396. DOI: 10.1021/jp810292n.
(18) Luchini, G.; Alegre-Requena, J. V.; Funes-Ardoiz, I.; Paton, R. S. GoodVibes: automated thermochemistry for heterogeneous computational chemistry data. *F1000Research* **2020**, *9* (291), 291. DOI: https://doi.org/10.12688/f1000research.22758.1.
